# Dihydroartemisinin Modulates Prostate Cancer Progression by Regulating Multiple Genes *via* the Transcription Factor NR2F2

**DOI:** 10.2174/0113892010311317240919061821

**Published:** 2024-10-03

**Authors:** Yong Shao, Yunhui Chan, Chuan Zhang, Rong Zhao, Yuxin Zu

**Affiliations:** 1 Department of Urology, The Second Affiliated Hospital of Harbin Medical University, Harbin, HeiLongJiang, 150001, China;; 2 Department of Urology, The Fifth Hospital of Cheng Du, Chengdu, 611130, China;; 3 Department of Cardiology, The Second Affiliated Hospital of Harbin Medical University, Harbin, HeiLongJiang, 150001, China;; 4 Department of Surgery, Suihua Hospital of Traditional Chinese Medicine, Suihua, HeiLongJiang, 152000, China

**Keywords:** DHA, NR2F2, transcription factor, DU145, PC-3 cells, prostate cancer

## Abstract

**Objective:**

This study aimed to investigate the effect of dihydroartemisinin (DHA) on DU145 cells and the role of NR2F2 (COUP-TFII) and its potential target genes in this process.

**Methods:**

GSE122625 was used to identify differentially expressed genes (DEGs) between the DHA-treated and control groups. Protein-protein interaction (PPI) network analysis was performed to identify hub genes, and the ChEA3 database was used to identify potential transcription factors. qRT-PCR and Western blot were used to validate the expression of genes of interest and functional assays were performed to evaluate the effect of DHA on DU145 and PC-3 cells. To solidify the regulatory relationship of NR2F2 with EFNB2, EBF1, ETS1, and VEGFA, a Chromatin Immunoprecipitation (ChIP) experiment was performed.

**Results:**

We identified 85 DEGs in DU145 cells treated with DHA, and PPI network analysis identified NR2F2 as a hub gene and potential transcription factor. The regulatory network of NR2F2 and its potential target genes (EFNB2, EBF1, ETS1, and VEGFA) was constructed, and the expression of these genes was upregulated in DHA-treated cells compared to control cells. Functional assays showed that DHA treatment inhibited epithelial-mesenchymal transition, reduced inflammation, and promoted apoptosis in DU145 and PC-3 cells. Furthermore, NR2F2 knockdown receded the DHA-induced upregulation of target genes and functional changes of DU145 and PC-3 cells. The outcomes of ChIP unequivocally pointed to a positive regulatory role of NR2F2 in these gene expressions.

**Conclusion:**

Our study suggests that DHA treatment affects the functions of DU145 and PC-3 cells by regulating the expression of NR2F2 and its potential target genes, and NR2F2 may serve as a potential therapeutic target for prostate cancer.

## 1 INTRODUCTION

Prostate cancer is a prevalent malignancy that accounts for approximately 20% of all newly diagnosed cancers in men worldwide [1]. Despite significant progress in diagnosis and treatment, advanced prostate cancer still has a high mortality rate [2, 3]. Therefore, there is an urgent need to identify novel therapeutic targets for this disease.

Artemisinin, which is derived from the annual Compositae family member *Artemisia annua* L., has been used as a traditional Chinese medicine for more than 2,000 years, and dihydroartemisinin (DHA) is the first generation derivative of this compound [4]. DHA, with a molecular formula of C_15_H_24_O_5_ and a molecular weight of 284.35, has displayed anticancer effects on many types of tumors, such as lung cancer, breast cancer, prostate cancer, ovarian cancer, and digestive system tumors. In general, DHA has been proven to have many anticancer effects, including inhibiting proliferation, inducing apoptosis, inhibiting tumor metastasis and angiogenesis, promoting immune function, and inducing autophagy and endoplasmic reticulum (ER) stress [4-8]. However, the molecular mechanism underlying the anti-cancer effects of DHA in prostate cancer remains poorly understood.

Transcription factors are critical regulators of gene expression that play a crucial role in various cellular processes, including proliferation, differentiation, and apoptosis [9-11]. Dysregulation of transcription factor activity is frequently observed in cancer, making them attractive targets for cancer therapy [9, 12]. In recent years, bioinformatics approaches have been widely used to identify potential transcription factors that play essential roles in cancer development and progression [9].

This study delves into the GSE122625 dataset from the GEO database to explore the impact of DHA on prostate cancer cell lines, specifically DU145 cells. By identifying differentially expressed genes (DEGs) and constructing protein-protein interaction (PPI) networks, we aim to elucidate the underlying molecular mechanisms of the anticancer effects of DHA. A key focus of our investigation is NR2F2 (nuclear receptor subfamily 2 group F member 2, also known as COUP-TFII, Chicken Ovalbumin Upstream Promoter Transcription Factor II), a hub gene and transcription factor identified as central to the regulatory network influenced by DHA treatment. Through a combination of bioinformatics analysis, qRT-PCR, Western blotting, and functional assays, we seek to provide a comprehensive understanding of how DHA modulates gene expression and cellular behavior in prostate cancer cells.

In summary, our research aims to build on the promising anticancer potential of DHA, investigating its effects on gene expression and cellular functions in prostate cancer cells. By understanding these mechanisms, we hope to contribute to developing DHA as a potential therapeutic agent for cancer treatment.

## 2 MATERIALS AND METHODS

### 2.1 Analyzing GEO Datasets

We utilized the GSE122625 dataset from the GEO database to investigate gene expression differences between two groups of DU145 cells: an experimental group treated with DHA and a control group treated with dimethyl sulfoxide (DMSO). Each group consisted of two biological replicates. Differential gene expression analysis was performed using the Limma package. We constructed a protein-protein interaction (PPI) network using the STRING database (https://string-db.org/) to elucidate the molecular mechanisms underlying the observed gene expression changes and identified hub genes using Cytoscape software. We further investigated potential transcription factors regulating the identified genes by querying the ChEA3 database (https://maayanlab.cloud/chea3/#top). The gene set GSVA, GSEA, and immune analyses were conducted using the Gene Set Cancer Analysis (GSCA) website at http://bioinfo.life.hust.edu.cn/GSCA/#/.

### 2.2 Cell Culture and Stimulation

DU145 and PC-3 cells (purchased from the agents of Cellverse (Shanghai) Cell Technology Co., LTD) were cultured in RPMI 1640 medium supplemented with 10% fetal bovine serum (FBS) and 1% penicillin-streptomycin at 37°C in a humidified incubator with 5% CO_2_. To investigate the effect of DHA on cell viability, DU145 cells were treated with different concentrations of DHA (5nM, 50nM, 0.5μM, 5μM, 50μM, and 500μM) for 24 h, and untreated cells were used as a control group. Cell viability was determined using a CCK-8 assay according to the manufacturer's instructions. Based on the results (Supplementary Fig. **1**), we selected three concentrations of DHA (0.5 μM, 5 μM, and 50 μM) for subsequent experiments. The cells were treated with DHA for the indicated time periods and then harvested for further analysis.

### 2.3 qRT-PCR Analysis

Total RNA was extracted from cells using TRIzol reagent (BBI, Shanghai, China). For mRNA analysis, cDNA was synthesized using a BeyoRT™ II cDNA synthesis kit (Beyotime, Shanghai, China). Real-time PCR was performed using SYBR Green Real-Time PCR Master Mixes (Beyotime, Shanghai, China) on an ABI StepOne (Plus) Real-Time PCR machine (Applied Biosystems). Three replicates were carried out in each group, and the relative quantitative method was used to analyze mRNA expression levels with internal reference GADPH, respectively (data were analyzed using the 2^(-△△CT) method).

### 2.4 Western Blot

We performed Western blot experiments to detect the expression levels of NR2F2, EFNB2, EBF1, ETS1, VEGFA, E-cadherin, and N-cadherin proteins in human DU145 DU145 and PC-3 cells. Proteins were extracted from cells using RIPA lysis buffer (Beyotime, Shanghai, China) containing protease inhibitors (Beyotime, Shanghai, China). Protein concentration was measured using a BCA protein assay kit (Beyotime, Shanghai, China). Equal amounts of protein were separated using 10% SDS-PAGE and then transferred onto a PVDF membrane (Millipore, Bedford, MA, USA). The membrane was blocked with 5% non-fat milk in TBST buffer (Beyotime, Shanghai, China) and then incubated with primary antibodies against NR2F2, EFNB2, EBF1, ETS1, VEGFA, E-cadherin, N-cadherin, and GAPDH (all purchased from ABclonal, Wuhan, China) at 4°C overnight. After washing with TBST buffer, the membrane was incubated with horseradish peroxidase-conjugated goat anti-rabbit IgG (ABclonal, Wuhan, China) secondary antibody at room temperature for 1 hour. The protein bands were visualized using an ECL detection system (Beyotime, Shanghai, China).

### 2.5 Scratch Assay

Scratch assay was performed to evaluate the cell migration ability of DU145 and PC-3 cells. Cells were cultured in 6-well plates until they reached 90% confluence. Scratches were made using a sterile 200 μL pipette tip, and the culture medium was replaced with a serum-free medium. Images of the scratches were captured at 0h, 12h, 24h, and 48h using an inverted microscope. The width of the scratch area was analyzed using Image J software to determine the extent of cell migration.

### 2.6 ELISA

We performed an enzyme-linked immunosorbent assay (ELISA) to measure the levels of interleukin-1β (IL-1β), interleukin-6 (IL-6), and tumor necrosis factor-α (TNF-α) in DU145 and PC-3 cells. After treatment with the desired compounds, the supernatants were collected and centrifuged at 3000 rpm for 10 minutes. The levels of cytokines were then quantified using commercially available ELISA kits (Beyotime, Shanghai, China) according to the manufacturer's instructions. Briefly, samples were collected and added to a 96-well ELISA plate, each containing the appropriate standards, negative controls, and samples as provided by the assay kit. The ELISA plate was incubated to ensure complete reactions in each well. After incubation, the ELISA plate was washed with a washing buffer to remove unbound components. Substrate solution was added, and the color reaction developed in the ELISA plate was observed. The reaction was stopped at the appropriate time. The OD of each well was measured using a microplate reader at the specified wavelength.

### 2.7 Hoechst Staining

The Hoechst staining assay was performed to detect apoptotic cells. DU145 and PC-3 cells were seeded onto coverslips and treated with DHA for 24 hours. After incubation, the cells were washed with PBS and fixed with 4% paraformaldehyde for 20 minutes. Then, the cells were stained with 5 μg/mL Hoechst 33258 solution (Sigma-Aldrich, St. Louis, MO, USA) for 10 minutes in the dark. The stained cells were washed twice with PBS and observed under a fluorescence microscope (Olympus, Tokyo, Japan) with excitation at 350 nm and emission at 460 nm. The apoptotic cells were identified by nuclear fragmentation and condensation. The percentage of apoptotic cells was calculated by counting the number relative to the total number of cells in five randomly selected fields. Three independent experiments were conducted for each treatment.

### 2.8 Cell Transfection

Cell transfection was performed to introduce NR2F2-siRNA into DU145 and PC-3 cells. The siRNA sequence was designed as sense (5'-3') GUGGAAUUUAUUGGCAGCCAAdAdc and anti-sense (5'-3') UUGGCUGCCAAUAAAUUCCACdAdc. The siRNA sequence was designed as sense (5'-3') UUCUCCGAACGUGUCACGUdTdT and anti-sense (5'-3') ACGUGACACGUUCGGAGAAdTdT. Both were designed and synthesized by Suzhou Hongxun Biotechnology Co., Ltd. Briefly, cells were seeded into a 6-well plate and allowed to grow overnight. The transfection mixture was prepared by mixing the NR2F2-siRNA and Lipo8000 (Beyotime, Shanghai, China) in OPTI-MEM (BBI, Shanghai, China) and incubated for 20 min at room temperature. The transfection mixture was then added to the cells and incubated for 6 h, after which the medium was replaced with a fresh, complete medium. The cells were harvested 48 h after transfection for further experiments.

### 2.9 Chromatin Immunoprecipitation

DU145 and PC-3 cells were cross-linked with 1% formaldehyde for 10 minutes at room temperature. Cross-linked cells were sonicated to obtain 200-500 base pairs of DNA fragments. Sheared chromatin was incubated with antibodies against NR2F2 or IgG (negative control) overnight at 4°C. Protein A/G magnetic beads were used for immunoprecipitation, followed by sequential washes and elution of chromatin complexes. Eluted chromatin was subjected to DNA purification using phenol-chloroform extraction and ethanol precipitation. qPCR was performed using primers targeting EFNB2, EBF1, ETS1, and VEGFA promoters. Enrichment was calculated as percent input. The enrichment of NR2F2-bound DNA fragments over IgG control was analyzed statistically.

### 2.10 Statistical Analysis

All the data were analyzed using SPSS 22.0 (IBM Corp., Armonk, NY, USA). The statistical significance was set at p < 0.05. Graphs and Figs. were generated using GraphPad Prism software. We conducted all experiments in triplicate. Specifically, we had three biological replicates per treatment group for the experiments involving triplicate cultures. The data in the Figs. reflect the mean results from three independent experiments.

## 3 RESULTS

### 3.1 Analyzation of GSE122625

**Fig. (1):**
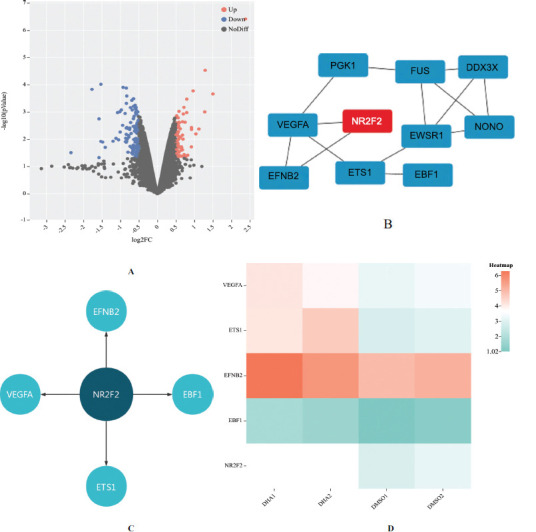
Analyzation of GSE122625. (**A**) Volcano plot of differentially expressed genes (DEGs) in DU145 cells treated with DHA compared to the control group treated with DMSO. Red dots indicate up-regulated genes, blue dots indicate down-regulated genes, and gray dots indicate non-DEGs. (**B**) For the top 10 hub genes identified by the cytoHubba plugin in the PPI network, the red label indicates trans factor NR2F2. (**C**) Regulatory network of NR2F2 and its potential target genes. (**D**) Heatmap of NR2F2 and its potential target genes in DU145 cells treated with DHA or DMSO. Red indicates up-regulated expression, and green indicates down-regulated expression.

This study analyzed the GSE122625 dataset sourced from the GEO database. Our analysis revealed 85 differentially expressed genes (DEGs) between DU145 cells treated with DHA and the control group treated with DMSO. This contrast in gene expression was visualized through the volcano plot presented in Fig. (**1A**). Furthermore, we constructed a protein-protein interaction (PPI) network utilizing the STRING website to delve deeper into the functional relationships among these DEGs. By employing the cytoHubba plugin, we pinpointed the top 10 hub genes within this network. The resulting PPI network of hub genes is depicted in Fig. (**1B**). Moreover, we employed the ChEA3 database to identify potential transcription factors that might regulate these hub genes (Supplementary Table **1**). Intriguingly, we uncovered that NR2F2 functions both as a hub gene and a transcription factor. Subsequently, we established a comprehensive regulatory network centered around NR2F2 (also known as COUP-TFII) and its potential target genes, encompassing four hub genes (EFNB2, EBF1, ETS1, and VEGFA) known as potential NR2F2 targets (Fig. **1C**). In addition, we explored the expression profiles of NR2F2 and its potential target genes in DU145 cells treated with either DHA or DMSO using a heatmap analysis. Notably, Fig. (**1D**) illustrates a significant up-regulation of NR2F2 expression in DU145 cells treated with DHA. The heatmap demonstrates the up-regulation of four potential target genes, EFNB2, EBF1, VEGFA, and ETS1, in DU145 cells treated with DHA compared to the control group. In summary, our analysis of the GSE122625 dataset uncovers 85 DEGs in response to DHA treatment in DU145 cells. Furthermore, we reveal the complex interactions among these genes through a PPI network, identify NR2F2 as a dual-role hub gene and transcription factor, and construct a regulatory network highlighting its potential target genes. The heatmap analysis then solidifies the upregulation of NR2F2 and its target genes upon DHA treatment.

### 3.2 Expression of NR2F2 and its Potential Target Genes are Affected by DHA

In this study, we conducted cell experiments using different concentrations of DHA (0.5μM, 5μM, and 50μM) to treat DU145 and PC-3 cells, with DMSO treatment and blank control as the control groups. qRT-PCR was used to detect the mRNA expression of NR2F2, EFNB2, EBF1, ETS1, and VEGFA, while Western blot was used to detect the protein expression of these genes. As shown in Fig. (**2A** and **B**), the qRT-PCR results indicated that the mRNA expression of NR2F2, EFNB2, EBF1, ETS1, and VEGFA exhibited a proportional increase with ascending concentrations of DHA. This observation underscores the influence of DHA on the transcriptional regulation of these genes. Similarly, as shown in Fig. (**2C** and **2D**), the Western blot results demonstrated that the protein expression of these genes also increased with increasing DHA concentration, indicating that the protein expression of NR2F2, EFNB2, EBF1, ETS1, and VEGFA follows a similar pattern. The Western blot analysis further supports the notion that DHA concentration influences the expression of these genes at the protein level. The results showed no significant change in the protein and mRNA expression of the DMSO group. This observation is an essential control in validating the specificity of the effects observed under DHA treatment. To sum up, the results from this segment underscore the concentration-dependent influence of DHA on both mRNA and protein expression levels of NR2F2, EFNB2, EBF1, ETS1, and VEGFA in DU145 and PC-3 cells. This aligns with our overarching investigation into the regulatory mechanisms impacted by DHA treatment.

### 3.3 DHA Treatment Affects DU145 Cell Function

**Fig. (3):**
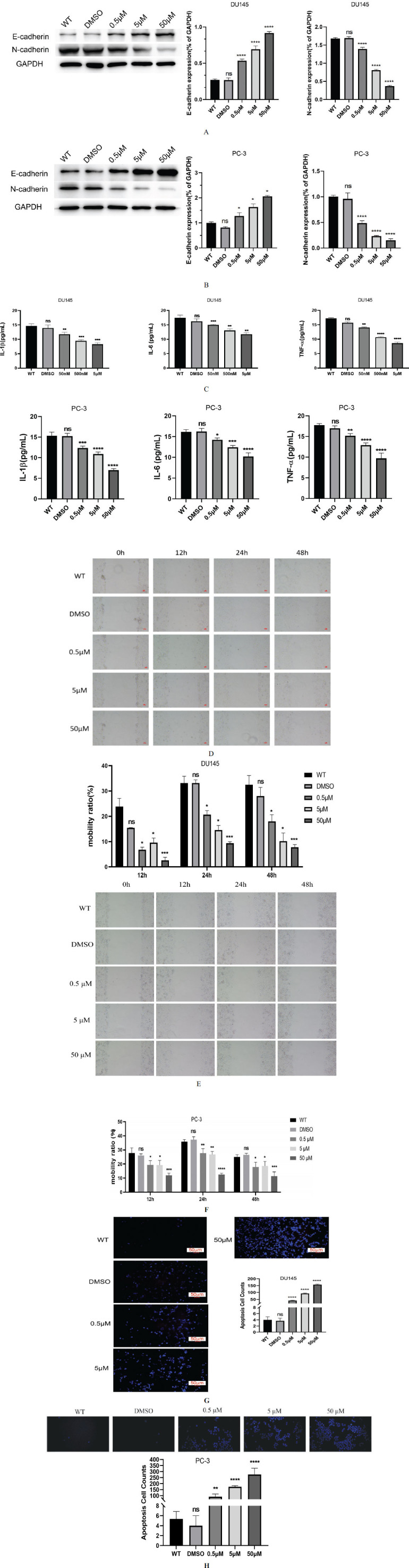
DHA treatment affects cell function. (**A**) Western blot analysis of DU145 cells illustrating the dose-dependent effect of DHA treatment on the expression of E-cadherin and N-cadherin, markers of epithelial-mesenchymal transition (EMT). (**B**) Western blot analysis of PC-3 cells illustrating the dose-dependent effect of DHA treatment on the expression of E-cadherin and N-cadherin, markers of EMT. (**C**) ELISA results indicate the dose-dependent reduction of pro-inflammatory cytokines IL-1β, IL-6, and TNF-α levels upon DHA treatment in the DU145 cells. (**D**) ELISA results of PC-3 cells indicate the dose-dependent reduction of pro-inflammatory cytokines IL-1β, IL-6, and TNF-α levels upon DHA treatment. (**E**) Scratch assay of DU145 cells demonstrating the impaired wound healing ability with increasing DHA concentrations at 12, 24, and 48-hour time points. (**F**) Scratch assay of PC-3 cells demonstrating the impaired wound healing ability with increasing DHA concentrations at 12, 24, and 48-hour time points. (**G**) Hoechst staining of DU145 cells revealed the dose-dependent increase in apoptosis following DHA treatment. WT group represents the blank control. (**H**) Hoechst staining of PC-3 cells revealed the dose-dependent increase in apoptosis following DHA treatment. WT group represents the blank control. ns *p* > 0.05, * *p* < 0.05, ** *p* < 0.01, *** *p* < 0.001, **** *p* < 0.0001.

The effects of DHA treatment on DU145 and PC-3 cells were evaluated through various experiments. Western Blot analysis showed a dose-dependent response to DHA treatment (Fig. **3A** and **B**). Notably, the expression of E-cadherin, a crucial marker for epithelial phenotype, increased, while N-cadherin, linked to the mesenchymal phenotype, demonstrated a decrease. This observation suggested that DHA treatment exerted regulatory control over the epithelial-mesenchymal transition (EMT) process in DU145 and PC-3 cells. ELISA results (Fig. **3C** and **D**) unveiled a consistent dose-dependent trend. Specifically, DHA treatment reduced the levels of pro-inflammatory cytokines, IL-1β, IL-6, and TNF-α. This signifies that DHA treatment suppressed inflammation within DU145 and PC-3 cells. Our scratch assay findings (Fig. **3E** and **F**) underscored a notable impairment in the wound-healing ability of DU145 and PC-3 cells. As DHA concentration increased, wound closure was significantly delayed at 12, 24, and 48-hour time points. This suggests that DHA treatment hindered cell migration, which is vital for metastasis processes. Hoechst staining demonstrated that apoptosis increased dose-dependent with DHA treatment (Fig. **3G** and **H**). The dose-dependent elevation in apoptosis observed upon DHA treatment indicated that DHA prompted programmed cell death in DU145 and PC-3 cells. These diverse experiments collectively revealed the multifaceted effects of DHA treatment on DU145 and PC-3 cells. The upregulation of E-cadherin coupled with the downregulation of N-cadherin signified EMT inhibition. The reduction in pro-inflammatory cytokine levels points towards anti-inflammatory effects. The diminished cell migration capacity signifies impaired metastatic potential. Lastly, promoting apoptosis underscores the role of DHA in facilitating programmed cell death. Thus, our findings collectively suggest that DHA treatment holds promise for inhibiting EMT, curbing inflammation, mitigating cell migration, and promoting apoptosis in DU145 and PC-3 cells.

### 3.4 NR2F2 Knockdown Recedes DHA-induced Up-regulation of Target Genes

**Fig. (4):**
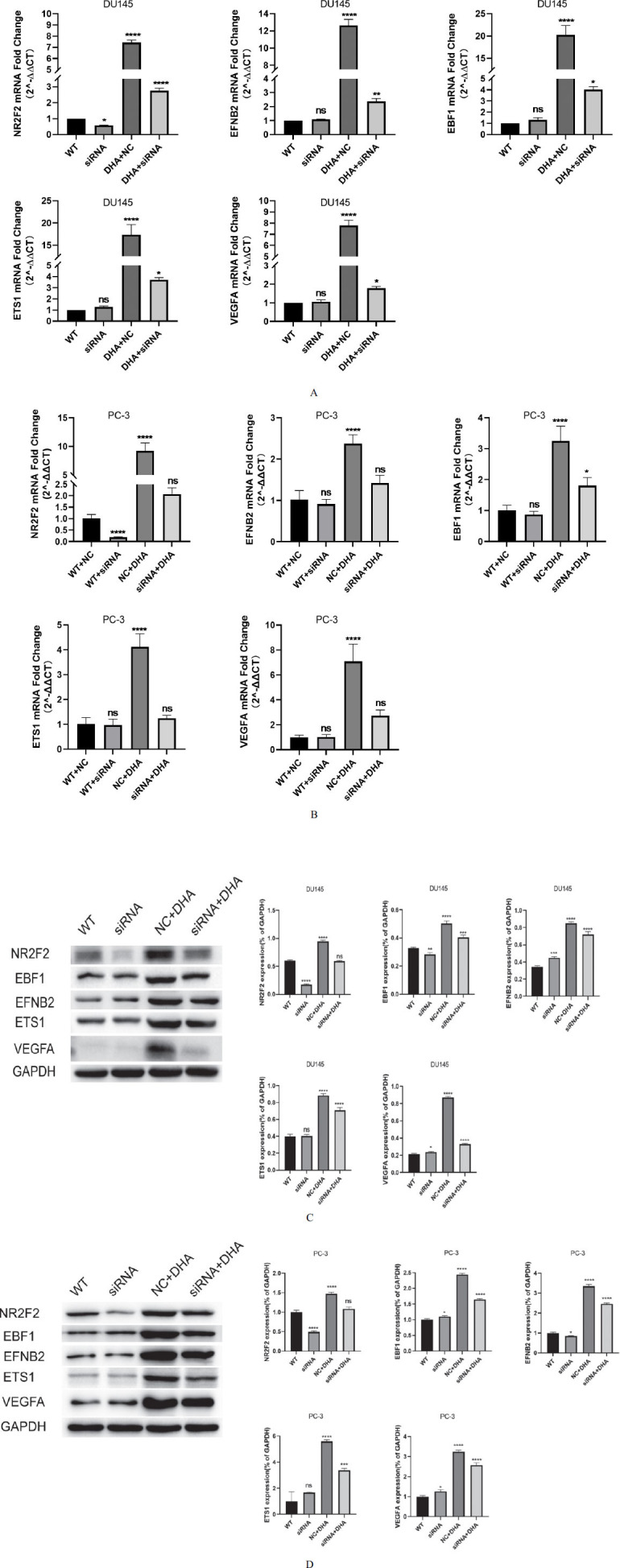
NR2F2 knockdown recedes DHA-induced up-regulation of target genes. (**A**) qRT-PCR results of DU145 cells showing the mRNA expression changes of NR2F2, EFNB2, EBF1, ETS1, and VEGFA in different experimental groups: siRNA group (transfected with si-NR2F2), DHA+siRNA group, and DHA+NC group. (**B**) qRT-PCR results of PC-3 cells showing the mRNA expression changes of NR2F2, EFNB2, EBF1, ETS1, and VEGFA in different experimental groups. (**C**) Western blot analysis of DU145 cells depicting the protein expression changes of the mentioned genes in the same experimental groups. WT group represents the blank control. (**D**) Western blot analysis of PC-3 cells depicting the protein expression changes of the mentioned genes in the same experimental groups. WT group represents the blank control. ns *p* > 0.05, * *p* < 0.05, ** *p* < 0.01, *** *p* < 0.001, **** *p* < 0.0001.

We aimed to investigate the effect of si-NR2F2 transfection on DU-145 and PC-3 cell expression and the influence of DHA treatment on these changes. The cells were divided into the WT group (blank control), siRNA group (transfected with si-NR2F2), DHA+siRNA group, and DHA+NC group. qRT-PCR was used to detect the mRNA expression of NR2F2, EFNB2, EBF1, ETS1, and VEGFA (Fig. **4A** and **B**), while Western blot was used to analyze the protein expression of NR2F2, EFNB2, EBF1, ETS1, and VEGFA (Fig. **4C** and **D**). The results showed that NR2F2 expression in the siRNA group was significantly reduced compared to the WT group, showcasing the effective knockdown of the target gene compared to the WT group. In contrast, EFNB2, EBF1, ETS1, and VEGFA expression remained unchanged. This observation suggests that the gene expression of these factors might not be exclusively regulated by NR2F2 alone. Other transcription factors or regulatory mechanisms within the cellular context contribute to maintaining the expression of these genes. Within the DHA+siRNA group, a compelling shift was observed. The expression levels of NR2F2, EFNB2, EBF1, ETS1, and VEGFA showed significant increases relative to the siRNA group. This suggests that DHA treatment bolsters the expression of these genes, even in the context of si-NR2F2 transfection. In the DHA+NC group, the expression of NR2F2, EFNB2, EBF1, ETS1, and VEGFA was the highest among all groups. This underscores the potent synergistic impact of DHA and si-NR2F2 transfection on gene expression modulation. In sum, our findings underscore the pivotal role of NR2F2 in regulating EFNB2, EBF1, ETS1, and VEGFA expression within DU-145 and PC-3 cells. Furthermore, the significant enhancement of these effects upon DHA treatment underscores the interplay between DHA and NR2F2 in orchestrating gene expression changes.

### 3.5 NR2F2 Knockdown Recedes DHA-induced Functional Change of DU-145 and PC-3 Cells

**Fig. (5):**
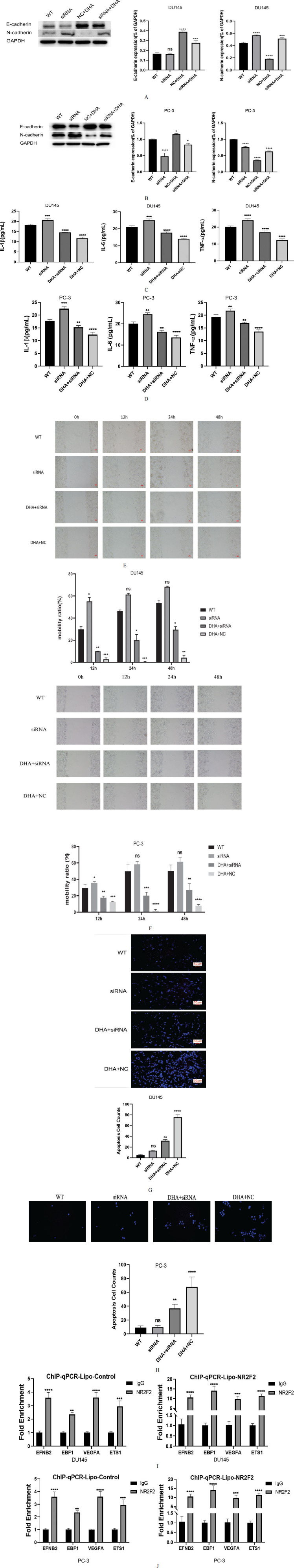
NR2F2 knockdown recedes DHA-induced functional change of DU-145 and PC-3 cells. (**A**) Western blot analysis highlighting the impact of NR2F2 knockdown and DHA treatment on EMT markers, E-cadherin and N-cadherin expression, in DU145 cells. (**B**) Western blot analysis highlighting the impact of NR2F2 knockdown and DHA treatment on EMT markers, E-cadherin and N-cadherin expression in PC-3 cells. (**C**) ELISA results display the modulation of pro-inflammatory cytokine levels (IL-1β, IL-6, and TNF-α) due to NR2F2 knockdown and DHA treatment in DU145 cells. (**D**) ELISA results display the modulation of pro-inflammatory cytokine levels (IL-1β, IL-6, and TNF-α) due to NR2F2 knockdown and DHA treatment in PC-3 cells. (**E**) Scratch assay outcomes illustrating the altered wound healing ability with NR2F2 knockdown and DHA treatment in DU145 cells. (**F**) Scratch assay outcomes illustrating the altered wound healing ability with NR2F2 knockdown and DHA treatment in PC-3 cells. (**G**) Hoechst staining indicates the changes in apoptosis rates in response to NR2F2 knockdown and DHA treatment in DU145 cells. (**H**) Hoechst staining shows the changes in apoptosis rates in response to NR2F2 knockdown and DHA treatment in PC-3 cells. (**I**) Chromatin Immunoprecipitation (ChIP) experiment validating the role of NR2F2 in positively regulating EFNB2, EBF1, ETS1, and VEGFA expression in DU145 cells. WT group represents the blank control. (**J**) Chromatin Immunoprecipitation (ChIP) experiment validating the role of NR2F2 in positively regulating EFNB2, EBF1, ETS1, and VEGFA expression in PC-3 cells. WT group represents the blank control. ns *p* > 0.05, * *p* < 0.05, ** *p* < 0.01, *** *p* < 0.001, **** *p* < 0.0001.

In line with the previous observations, Western blot analysis (Fig. **5A** and **B**) illuminated the intricacies of EMT regulation. E-cadherin expression exhibited no significant changes in the siRNA group, experienced an increase in the DHA+siRNA group, and attained its highest levels in the DHA+NC group. Conversely, N-cadherin expression was elevated in the siRNA group, decreased in the DHA+siRNA group, and the lowest levels were observed in the DHA+NC group, underscoring the dynamic role of NR2F2 and DHA in EMT modulation. ELISA results showed that IL-1β, IL-6, and TNF-α levels were elevated in the siRNA group, decreased in the DHA+siRNA group, and lowest in the DHA+NC group (Fig. **5C** and **D**), attesting to the combined effect of DHA and NR2F2 modulation. The scratch assay showed that scratch closure was enhanced in the siRNA group, reduced in the DHA+siRNA group, and lowest in the DHA+NC group (Fig. **5E** and **F**). Hoechst staining revealed that apoptosis was unchanged in the siRNA group, increased in the DHA+siRNA group, and highest in the DHA+NC group (Fig. **5G** and **H**). To solidify the regulatory relationship of NR2F2 with EFNB2, EBF1, ETS1, and VEGFA, a Chromatin Immunoprecipitation (ChIP) experiment was performed (Fig. **5I** and **J**). The outcomes unequivocally pointed to a positive regulatory role of NR2F2 in these gene expressions, affirming its significance in orchestrating their modulation. In sum, the findings presented in this section collectively underscore the intricate interplay between NR2F2, DHA, and the regulatory mechanisms governing EMT, inflammation, cell migration, and apoptosis in DU145 and PC-3 cells. The supplementary ChIP experiment highlighted the role of NR2F2 in directly influencing the expression of EFNB2, EBF1, ETS1, and VEGFA.

### 3.6 Comprehensive Analysis of Gene Expression Patterns

**Fig. (6):**
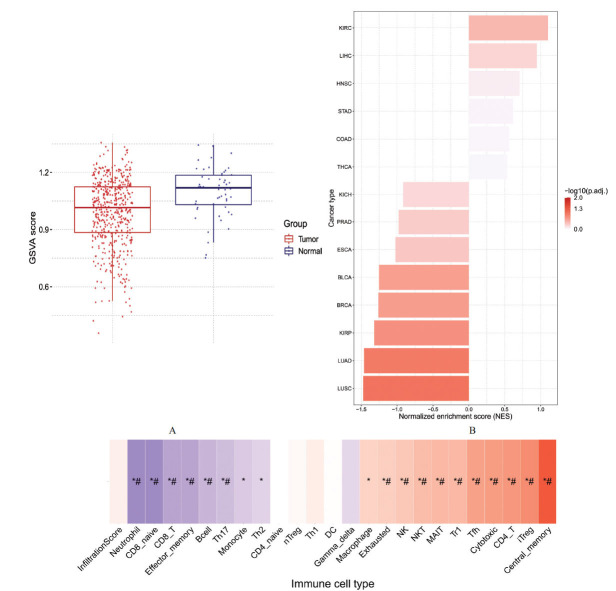
Bioinformatics analysis of the gene set. (**A**) Prostate cancer exhibits a lower GSVA score than normal tissues, indicating altered gene set enrichment in tumor samples. (**B**) GSEA scores demonstrate significant variations across 33 cancer types, highlighting the diverse impact of the gene set in various tumor contexts. (**C**) Immune-related analysis reveals a complex correlation pattern of the gene set with different immune cell subgroups. *: *P* value ≤ 0.05; #: FDR ≤ 0.05.

We observed distinct expression patterns of five genes (EBF1, EFNB2, ETS1, NR2F2, and VEGFA) following DHA treatment, deviating from prior research impressions. To comprehensively explore the collective behavior of these genes, we conducted further bioinformatics analysis. In prostate cancer, the tumor GSVA score was lower than the normal tissue score (Fig. **6A**). However, when analyzing a cohort of 33 tumors, we identified substantial variations in GSEA scores across diverse cancer types (Fig. **6B**). Upon conducting immune-related analyses, we found that this gene set negatively correlated with immune cell subgroups, including neutrophil, CD8 naive, CD8 T, effector memory, Bcell, Th17, monocyte, and Th2. Simultaneously, a positive correlation was observed with monocyte, Th2, CD4 naive, nTreg, Th1, DC, Gamma delta, macrophage, exhausted, NK, NKT, MAIT, Tr1, Tfh, cytotoxic, CD4 T, and iTreg (Fig. **6C**). These findings suggest that the gene set may play a role in a more intricate and diverse network of functions and regulatory pathways in tumors, challenging the previous notion of a simplistic portrayal as merely oncogenic.

## 4 DISCUSSION

The anti-tumor effect of DHA has been reported in various types of cancer, including prostate cancer [4, 5, 8, 13]. However, the intricate molecular mechanisms governing its anti-tumor effects remain an area of ongoing investigation. This study aimed to shed light on the impact of DHA on prostate cancer cells and unravel the underlying molecular pathways at play.

Our analysis of the GSE122625 dataset revealed 85 differentially expressed genes (DEGs) in DU145 cells treated with DHA compared to controls. Among these, NR2F2 (COUP-TFII) emerged as a central hub gene and a transcription factor, suggesting its critical role in regulating downstream genes involved in cancer progression. NR2F2 influences various cellular processes, including proliferation, differentiation, apoptosis, and metabolism, and plays a role in striated muscle development and various cancers. [14-22]. Dysregulation of the miRNAs-COUP-TFII-FOXM1-CENPF axis contributes to prostate cancer metastasis, with microRNA-382 inhibiting prostate cancer cell proliferation and metastasis through targeting COUP-TFII [23-25]. COUP-TFII (NR2F2) promotes prostate tumorigenesis by inhibiting TGF-β-induced growth barriers and correlates with increased lymphangiogenesis and lymph node metastasis in prostate adenocarcinoma [26, 27]. It also regulates MPC1, a key gene in cancer metabolism, highlighting its importance in prostate cancer's metabolic pathways [28]. NR2F2 serves as both a prognostic marker and a therapeutic target for prostate cancer, with alterations in its expression influencing therapeutic resistance mechanisms [29, 30]. Targeting NR2F2 with small-molecule drugs shows promise for therapeutic effects in prostate cancer treatment [31, 32].

Our study, through bioinformatics analysis and experimental methods, confirmed that NR2F2, along with its target genes EFNB2, EBF1, ETS1, and VEGFA, forms a regulatory network central to tumorigenesis. These genes have been linked to critical aspects of cancer progression. EFNB2, also known as ephrin-B2, is a transmembrane ligand that can activate Eph receptor tyrosine kinases and has been shown to promote cancer cell proliferation, invasion, and angiogenesis [33, 34]. EBF1, or early B cell factor 1, has been implicated in the development of prostate cancer through its regulation of androgen receptor (AR) signaling and the androgen response [35-37]. ETS1 is up-regulated in prostate cancer and can promote tumor growth and metastasis by regulating genes involved in cell migration, invasion, and angiogenesis [38-40]. VEGFA, or vascular endothelial growth factor A, is a crucial mediator of angiogenesis and is over-expressed in prostate cancer, promoting tumor growth and metastasis by enhancing vascularization [41-44]. Identifying NR2F2 as a regulator of these genes positions it as a pivotal player in the anti-tumor activity of DHA.

Further experimental validation demonstrated that DHA treatment up-regulated NR2F2 and its target genes, suggesting that the anti-tumor effects of DHA are mediated through this regulatory axis. The modulation of EMT markers and the reduction of pro-inflammatory cytokines further support the role of DHA in inhibiting EMT and inflammation. Additionally, the ability of DHA to impede cell migration and promote apoptosis underscores its multifaceted anti-tumor mechanisms.

To delve deeper into the underlying mechanisms, we executed si-NR2F2 transfection experiments. The knockdown of NR2F2 attenuated the DHA-induced up-regulation of EFNB2, EBF1, ETS1, and VEGFA, confirming the regulatory influence of NR2F2. Moreover, the functional effects of DHA, such as inhibition of EMT, reduction of inflammation, and induction of apoptosis, were mitigated by NR2F2 knockdown, highlighting the centrality of NR2F2 in mediating these responses.

Our bioinformatics analysis revealed distinct expression patterns of the NR2F2 gene set across various cancers, suggesting its involvement in diverse molecular pathways. The immune-related correlations indicated complex interactions between the gene set and immune cell subgroups, hinting at potential immunomodulatory roles. These findings suggest that the gene set's role in cancer biology is more intricate and context-dependent than previously thought.

The potential of DHA as an anticancer agent has been highlighted in studies by Xia *et al*. and Lee *et al*. [13, 30], which align closely with our findings. These investigations, like ours, observed the remarkable ability of DHA to inhibit cancer growth and induce apoptosis, reinforcing its therapeutic significance. Our investigation extends this recognition by contextualizing the effects of DHA within a molecular network involving NR2F2 and its target genes. Additionally, Paccez *et al*. elucidated the inhibitory effect of DHA on prostate cancer through the JARID2/miR-7/miR-34a pathway, which aligns with our understanding and underscores the multifaceted regulatory mechanisms of DHA in prostate cancer [45].

Our study sheds light on the molecular mechanisms underlying the anti-tumor effects of DHA in prostate cancer. By identifying NR2F2 as a central regulator, we provide a deeper understanding of how DHA modulates key genes and pathways involved in tumor progression. These insights contribute to the evolving narrative of the potential of DHA as a therapeutic agent in prostate cancer, offering a foundation for future research and therapeutic strategies.

## CONCLUSION

In conclusion, the present study proves that DHA treatment can inhibit EMT and reduce inflammation and cell migration while promoting apoptosis in prostate cancer cells. NR2F2 (COUP-TFII) plays a critical role in mediating the effects of DHA treatment on EFNB2, EBF1, ETS1, and VEGFA expression, and the knockdown of NR2F2 can recede the functional changes induced by DHA treatment in prostate cancer cells. These findings provide new insights into the molecular mechanisms underlying the anti-tumor effects of DHA and may have implications for the development of new therapies for prostate cancer.

## Figures and Tables

**Fig. (2):**
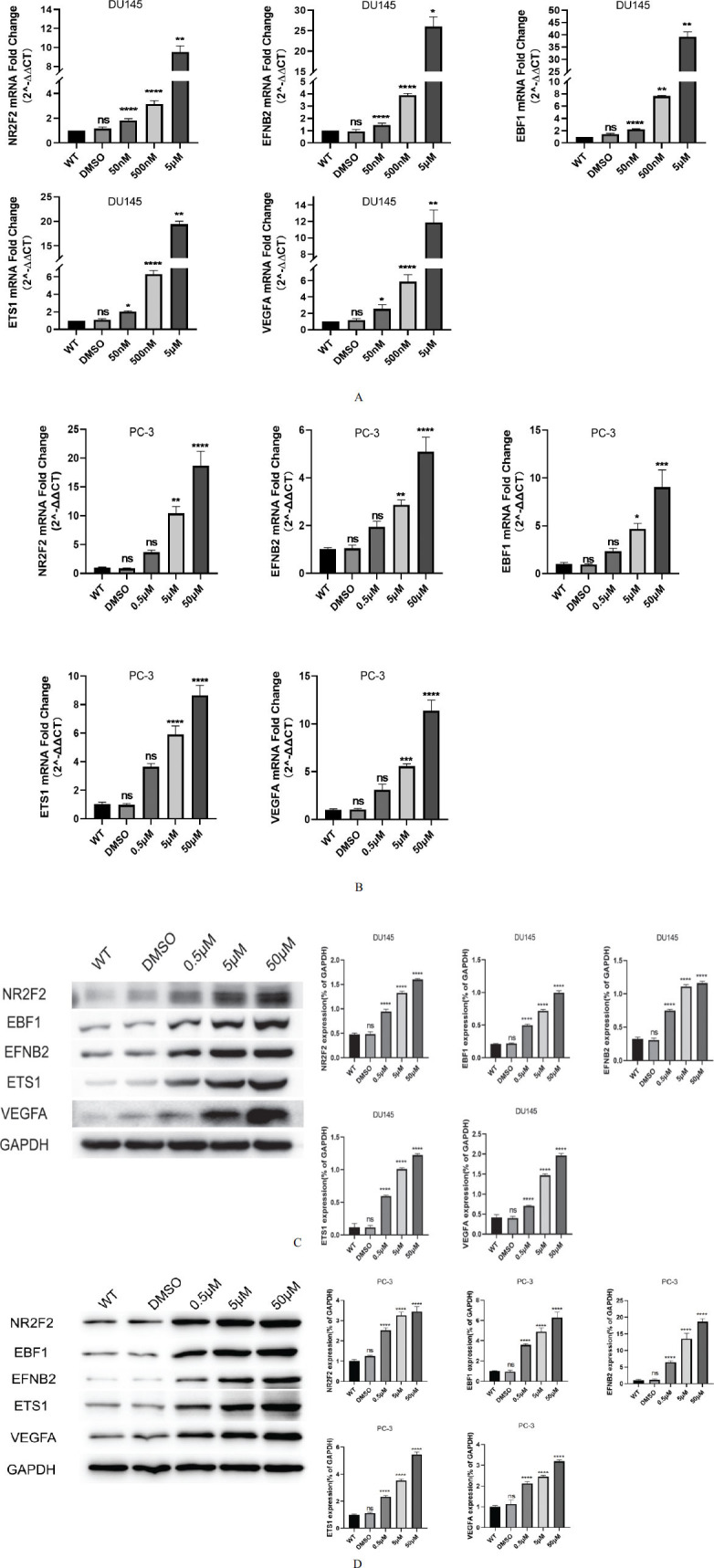
Expression of NR2F2 and its potential target genes are affected by DHA. (**A**) qRT-PCR results showing the dose-dependent increase in mRNA expression of NR2F2, EFNB2, EBF1, ETS1, and VEGFA in DU145 cells treated with different concentrations of DHA (0.5 μM, 5 μM, and 50 μM) compared to the blank control group (WT) and the DMSO-treated control group. (**B**) qRT-PCR results showing the dose-dependent increase in mRNA expression of NR2F2, EFNB2, EBF1, ETS1, and VEGFA in PC-3 cells treated with different concentrations of DHA (0.5 μM, 5 μM, and 50 μM) compared to the blank control group (WT) and the DMSO-treated control group. (**C**) Western blot analysis demonstrates the corresponding dose-dependent increase in protein expression of the mentioned genes upon DHA treatment in DU145 cells. WT group represents the blank control. (**D**) Western blot analysis demonstrates the corresponding dose-dependent increase in protein expression of the mentioned genes upon DHA treatment. The WT group represents the blank control in PC-3 cells. ns *p* > 0.05, * *p* < 0.05, ** *p* < 0.01, *** *p* < 0.001, **** *p* < 0.0001.

## Data Availability

The data and supportive information are available within the article.

## LIST OF ABBREVIATIONS

DHA: Dihydroartemisinin
NR2F2: Nuclear receptor subfamily 2 group F member 2
COUP-TFII: Chicken ovalbumin upstream promoter-transcriptional factor II
DEGs: Differentially Expressed Genes
PPI: Protein-Protein Interaction
qRT-PCR: Quantitative Real-Time Polymerase Chain Reaction
EFNB2: Ephrin B2
EBF1: EBF transcription factor 1
ETS1: ETS proto-oncogene 1, transcription factor
VEGFA: Vascular endothelial growth factor A
ChIP: Chromatin Immunoprecipitation
ER: Endoplasmic reticulum
GEO: Gene Expression Omnibus
DMSO: Dimethyl Sulfoxide
FBS: Fetal Bovine Serum
SDS-PAGE: Sodium Dodecyl Sulfate Polyacrylamide Gel Electrophoresis
PVDF: Polyvinylidene Fluoride
BCA: Bicinchoninic Acid
CCK-8: Cell Counting Kit-8
ELISA: Enzyme-Linked Immunosorbent Assay
IL: Interleukin
TNF-α: Tumor Necrosis Factor Alpha
EMT: Epithelial-Mesenchymal Transition
GSVA: Gene Set Variation Analysis
GSEA: Gene Set Enrichment Analysis
RNA: Ribonucleic Acid
mRNA: Messenger RNA
cDNA: Complementary DNA
GADPH: Glyceraldehyde-3-Phosphate Dehydrogenase
siRNA: Small Interfering RNA
GEO: Gene Expression Omnibus
SPSS: Statistical Package for the Social Sciences

## References

[1] Wang L., Lu B., He M., Wang Y., Wang Z., Du L. (2022). Prostate Cancer Incidence and Mortality: Global Status and Temporal Trends in 89 Countries From 2000 to 2019.. Front. Public Health.

[2] Culp M.B., Soerjomataram I., Efstathiou J.A., Bray F., Jemal A. (2020). Recent Global Patterns in Prostate Cancer Incidence and Mortality Rates.. Eur. Urol..

[3] Taitt H.E. (2018). Global Trends and Prostate Cancer: A Review of Incidence, Detection, and Mortality as Influenced by Race, Ethnicity, and Geographic Location.. Am. J. Men Health.

[4] Dai X., Zhang X., Chen W., Chen Y., Zhang Q., Mo S., Lu J. (2021). Dihydroartemisinin: A Potential Natural Anticancer Drug.. Int. J. Biol. Sci..

[5] Yong J., Lu C., Olatunde O.Z. (2023). An Overview of Dihydroartemisinin as a Promising Lead Compound for Development of Anticancer Agents.. Mini Rev. Med. Chem..

[6] Gour R., Ahmad F., Prajapati S.K., Giri S.K., Lal Karna S.K., Kartha K.P.R., Pokharel Y.R. (2019). Synthesis of novel S-linked dihydroartemisinin derivatives and evaluation of their anticancer activity.. Eur. J. Med. Chem..

[7] He Q., Shi J., Shen X.L., An J., Sun H., Wang L., Hu Y.J., Sun Q., Fu L.C., Sheikh M.S., Huang Y. (2010). Dihydroartemisinin upregulates death receptor 5 expression and cooperates with TRAIL to induce apoptosis in human prostate cancer cells.. Cancer Biol. Ther..

[8] Du S., Xu G., Zou W., Xiang T., Luo Z. (2017). Effect of dihydroartemisinin on UHRF1 gene expression in human prostate cancer PC-3 cells.. Anticancer Drugs.

[9] Dey A., Sen S., Maulik U. (2022). Study of transcription factor druggabilty for prostate cancer using structure information, gene regulatory networks and protein moonlighting.. Brief. Bioinform..

[10] Singh A., Happel C., Manna S.K., Acquaah-Mensah G., Carrerero J., Kumar S., Nasipuri P., Krausz K.W., Wakabayashi N., Dewi R., Boros L.G., Gonzalez F.J., Gabrielson E., Wong K.K., Girnun G., Biswal S. (2013). Transcription factor NRF2 regulates miR-1 and miR-206 to drive tumorigenesis.. J. Clin. Invest..

[11] Kumar V., Cheng P., Condamine T., Mony S., Languino L.R., McCaffrey J.C., Hockstein N., Guarino M., Masters G., Penman E., Denstman F., Xu X., Altieri D.C., Du H., Yan C., Gabrilovich D.I. (2016). CD45 Phosphatase Inhibits STAT3 Transcription Factor Activity in Myeloid Cells and Promotes Tumor-Associated Macrophage Differentiation.. Immunity.

[12] Bushweller J.H. (2019). Targeting transcription factors in cancer — from undruggable to reality.. Nat. Rev. Cancer.

[13] Xia T., Liu S., Xu G., Zhou S., Luo Z. (2022). Dihydroartemisinin induces cell apoptosis through repression of UHRF1 in prostate cancer cells.. Anticancer Drugs.

[14] Liang Y., Wu X., Lee J., Yu D., Su J., Guo M., Meng N., Qin J., Fan X. (2022). lncRNA NR2F2-AS1 inhibits the methylation of miR-494 to regulate oral squamous cell carcinoma cell proliferation.. Arch. Oral Biol..

[15] Ma L., Huang M., Liao X., Cai X., Wu Q. (2022). NR2F2 Regulates Cell Proliferation and Immunomodulation in Whartons’ Jelly Stem Cells.. Genes (Basel).

[16] Mauri F., Schepkens C., Lapouge G., Drogat B., Song Y., Pastushenko I., Rorive S., Blondeau J., Golstein S., Bareche Y., Miglianico M., Nkusi E., Rozzi M., Moers V., Brisebarre A., Raphaël M., Dubois C., Allard J., Durdu B., Ribeiro F., Sotiriou C., Salmon I., Vakili J., Blanpain C. (2021). NR2F2 controls malignant squamous cell carcinoma state by promoting stemness and invasion and repressing differentiation.. Nat. Cancer.

[17] Cai Y.Y. (2022). NR2F2 mediates cell growth and endocrine resistance in NF1 loss, ER+ breast cancer.. Cancer Res..

[18] Cristina Fugazza, Gloria Barbarani, Sudharshan Elangovan, Serena Giolitto, Isaura Font-Monclus, Laura Manunza, John Strouboulis, Claudio Cantù, Fabio Gasparri, Yukio Nakamura, Sergio Ottolenghi, Paolo Moi, Nakamura Y., Ottolenghi S., Moi P., Ronchi A.E. (2020). The Coup-TFII orphan nuclear receptor is an activator of the γ-globin gene.. Haematologica.

[19] Polvani S., Pepe S., Milani S., Galli A. (2019). COUP-TFII in Health and Disease.. Cells.

[20] Bao Y., Lu Y., Feng W., Yu H., Guo H., Tao Y., Shi Q., Chen W., Wang X. (2019). COUP‑TFII promotes epithelial‑mesenchymal transition by inhibiting miR‑34a expression in colorectal cancer.. Int. J. Oncol..

[21] Erdős E., Bálint B.L. (2019). COUP-TFII is a modulator of cell-type-specific genetic programs based on genomic localization maps.. J. Biotechnol..

[22] Ashraf U.M., Sanchez E.R., Kumarasamy S. (2019). COUP-TFII revisited: Its role in metabolic gene regulation.. Steroids.

[23] Lin S.C., Kao C.Y., Lee H.J., Creighton C.J., Ittmann M.M., Tsai S.J., Tsai S.Y., Tsai M.J. (2016). Dysregulation of miRNAs-COUP-TFII-FOXM1-CENPF axis contributes to the metastasis of prostate cancer.. Nat. Commun..

[24] Zhang W., Liu J., Qiu J., Fu X., Tang Q., Yang F., Zhao Z., Wang H. (2016). MicroRNA-382 inhibits prostate cancer cell proliferation and metastasis through targeting COUP-TFII.. Oncol. Rep..

[25] Yun S.H., Park J.I. (2020). Recent progress on the role and molecular mechanism of chicken ovalbumin upstream promoter-transcription factor II in cancer.. J. Int. Med. Res..

[26] Qin J., Wu S.P., Creighton C.J., Dai F., Xie X., Cheng C.M., Frolov A., Ayala G., Lin X., Feng X.H., Ittmann M.M., Tsai S.J., Tsai M.J., Tsai S.Y. (2013). COUP-TFII inhibits TGF-β-induced growth barrier to promote prostate tumorigenesis.. Nature.

[27] Lilis I. (2018). The expression of p-mTOR and COUP-TFII correlates with increased lymphangiogenesis and lymph node metastasis in prostate adenocarcinoma.. Urol Oncol..

[28] Wang L., Xu M., Qin J., Lin S.C., Lee H.J., Tsai S.Y., Tsai M.J. (2016). MPC1, a key gene in cancer metabolism, is regulated by COUPTFII in human prostate cancer.. Oncotarget.

[29] Qin J., Tsai S., Tsai M.J. (2013). COUP-TFII, a prognostic marker and therapeutic target for prostate cancer.. Asian J. Androl..

[30] Lee H.C., Ou C.H., Huang Y.C., Hou P.C., Creighton C.J., Lin Y.S., Hu C.Y., Lin S.C. (2021). YAP1 overexpression contributes to the development of enzalutamide resistance by induction of cancer stemness and lipid metabolism in prostate cancer.. Oncogene.

[31] Safe S., Jin U.H., Hedrick E., Reeder A., Lee S.O. (2014). Minireview: role of orphan nuclear receptors in cancer and potential as drug targets.. Mol. Endocrinol..

[32] Wang L., Cheng C.M., Qin J., Xu M., Kao C.Y., Shi J., You E., Gong W., Rosa L.P., Chase P., Scampavia L., Madoux F., Spicer T., Hodder P., Xu H.E., Tsai S.Y., Tsai M.J. (2020). Small-molecule inhibitor targeting orphan nuclear receptor COUP-TFII for prostate cancer treatment.. Sci. Adv..

[33] Xu C., Gu L., Kuerbanjiang M., Jiang C., Hu L., Liu Y., Xue H., Li J., Zhang Z., Xu Q. (2023). Adaptive activation of EFNB2/EPHB4 axis promotes post-metastatic growth of colorectal cancer liver metastases by LDLR-mediated cholesterol uptake.. Oncogene.

[34] Inoue C., Miki Y., Saito-Koyama R., Kobayashi K., Seyama K., Okada Y., Sasano H. (2022). Vasohibin-1 and -2 in pulmonary lymphangioleiomyomatosis (LAM) cells associated with angiogenic and prognostic factors.. Pathol. Res. Pract..

[35] Shuang O., Zhou J., Cai Z., Liao L., Wang Y., Wang W., Xu M. (2022). EBF1-mediated up-regulation of lncRNA FGD5-AS1 facilitates osteosarcoma progression by regulating miR-124-3p/G3BP2 axis as a ceRNA.. J. Orthop. Surg. Res..

[36] Luo H., Yang L., Liu C., Wang X., Dong Q., Liu L., Wei Q. (2020). TMPO-AS1/miR-98-5p/EBF1 feedback loop contributes to the progression of bladder cancer.. Int. J. Biochem. Cell Biol..

[37] Qiu K., Zheng Z., Huang Y. (2020). Long intergenic noncoding RNA 00844 promotes apoptosis and represses proliferation of prostate cancer cells through upregulating GSTP1 by recruiting EBF1.. J. Cell. Physiol..

[38] Xu S., Ge J., Zhang Z., Zhou W. (2017). MiR-129 inhibits cell proliferation and metastasis by targeting ETS1 *via* PI3K/AKT/mTOR pathway in prostate cancer.. Biomed. Pharmacother..

[39] Gu Y., Wu S., Chong Y., Guan B., Li L., He D., Wang X., Wang B., Wu K. (2022). DAB2IP regulates intratumoral testosterone synthesis and CRPC tumor growth by ETS1/AKR1C3 signaling.. Cell. Signal..

[40] Lu G., Zhang Q., Huang Y., Song J., Tomaino R., Ehrenberger T., Lim E., Liu W., Bronson R.T., Bowden M., Brock J., Krop I.E., Dillon D.A., Gygi S.P., Mills G.B., Richardson A.L., Signoretti S., Yaffe M.B., Kaelin W.G. (2014). Phosphorylation of ETS1 by Src family kinases prevents its recognition by the COP1 tumor suppressor.. Cancer Cell.

[41] Ma J., Chen X., Chen Y., Tao N., Qin Z. (2022). Ligustilide Inhibits Tumor Angiogenesis by Downregulating VEGFA Secretion from Cancer-Associated Fibroblasts in Prostate Cancer *via* TLR4.. Cancers (Basel).

[42] Ganapathy K., Staklinski S., Hasan M.F., Ottman R., Andl T., Berglund A.E., Park J.Y., Chakrabarti R. (2020). Multifaceted Function of MicroRNA-299-3p Fosters an Antitumor Environment Through Modulation of Androgen Receptor and VEGFA Signaling Pathways in Prostate Cancer.. Sci. Rep..

[43] Mushimiyimana I., Tomas Bosch V., Niskanen H., Downes N.L., Moreau P.R., Hartigan K., Ylä-Herttuala S., Laham-Karam N., Kaikkonen M.U. (2021). Genomic Landscapes of Noncoding RNAs Regulating *VEGFA* and *VEGFC* Expression in Endothelial Cells.. Mol. Cell. Biol..

[44] Mu H.Q., He Y.H., Wang S.B., Yang S., Wang Y.J., Nan C.J., Bao Y.F., Xie Q.P., Chen Y.H. (2020). MiR-130b/TNF-α/NF-κB/VEGFA loop inhibits prostate cancer angiogenesis.. Clin. Transl. Oncol..

[45] Paccez J.D., Duncan K., Sekar D., Correa R.G., Wang Y., Gu X., Bashin M., Chibale K., Libermann T.A., Zerbini L.F. (2019). Dihydroartemisinin inhibits prostate cancer *via* JARID2/miR-7/miR-34a-dependent downregulation of Axl.. Oncogenesis.

