# Supplementary material for: Dihydroartemisinin Modulates Prostate Cancer Progression by Regulating Multiple Genes via the Transcription Factor NR2F2
Source: Curr Pharm Biotechnol. 2024 Oct 3;26(6):935–55. doi: 10.2174/0113892010311317240919061821 (PMC12281655; doi:10.2174/0113892010311317240919061821)
Supplement: Supplementary file 1 [file CPB-26-6-935_SD1.pdf]

Supplementary Material

Dihydroartemisinin Modulates Prostate Cancer Progression by Regulating Multiple Genes via the Transcription Factor NR2F2

Yong Shao[1,#,*], Yunhui Chan[1,#], Chuan Zhang[2], Rong Zhao[3] and Yuxin Zu[4]

[1]Department of Urology, The Second Affiliated Hospital of Harbin Medical University, Harbin, HeiLongJiang, 150001, China; [2]Department of Urology, The Fifth Hospital of Cheng Du, Chengdu, 611130, China; [3]Department of Cardiology, The Second Affiliated Hospital of Harbin Medical University, Harbin, HeiLongJiang, 150001, China; [4]Department of Surgery, Suihua Hospital of Traditional Chinese Medicine, Suihua, HeiLongJiang, 152000, China

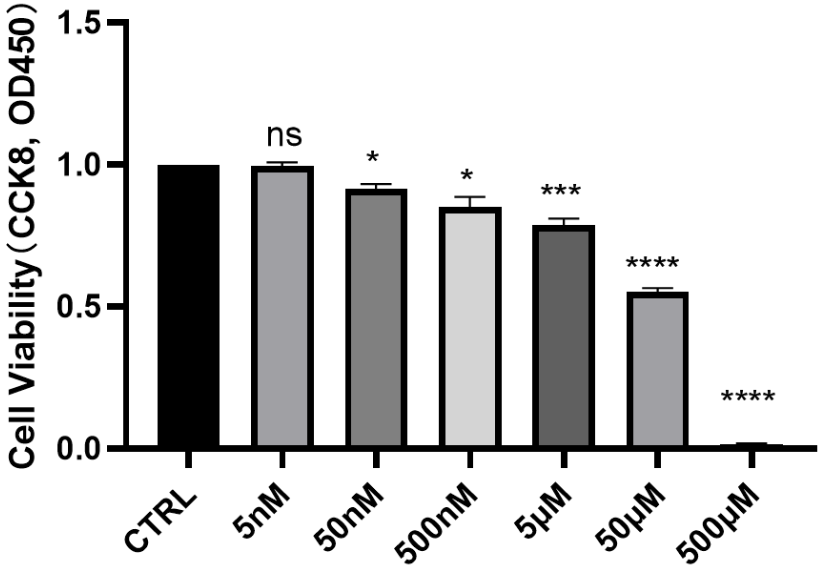

Figure S1. Results of cell culture and stimulation.

Table S1. Hub genes rank.

Rank	TF	Library	Overlapping_Genes
1	GTF2I	ARCHS4 Coexpression,50	FUS,NONO
2	HOXD8	ARCHS4 Coexpression,100;Enrichr Queries,78;GTEx Coexpression,17	EFNB2,EBF1,NR2F2,ETS1
3	DACH1	Literature ChIP-seq,124;ARCHS4 Coexpression,65;Enrichr Queries,10	EFNB2,EBF1,NR2F2,ETS1,VEGFA
4	SALL1	Literature ChIP-seq,24;ARCHS4 Coexpression,56;Enrichr Queries,18;GTEx Coexpression,169	EFNB2,DDX3X,NONO,EBF1,NR2F2,ETS1,VEGFA
5	BCL6B	ARCHS4 Coexpression,84;Enrichr Queries,115;GTEx Coexpression,45	EFNB2,EBF1,ETS1,VEGFA
6	KDM2A	ARCHS4 Coexpression,15;GTEx Coexpression,150	EWSR1,FUS,ETS1
7	HOXD4	ARCHS4 Coexpression,161;Enrichr Queries,75;GTEx Coexpression,23	EFNB2,EBF1,NR2F2,ETS1
8	NR2F2	ARCHS4 Coexpression,107;ENCODE ChIP-seq,50;Enrichr Queries,99;ReMap ChIP-seq,37;GTEx Coexpres-sion,139	EFNB2,EBF1,NR2F2,ETS1,VEGFA
9	NFIB	Literature ChIP-seq,143;ARCHS4 Coexpression,175;Enrichr Queries,20;GTEx Coexpression,36	EFNB2,EBF1,NR2F2,ETS1,VEGFA
10	TCF21	Literature ChIP-seq,44;ARCHS4 Coexpression,123;Enrichr Queries,13;GTEx Coexpression,199	EFNB2,EBF1,NR2F2,ETS1,VEGFA
11	KMT2B	ARCHS4 Coexpression,162;ReMap ChIP-seq,54	FUS,ETS1
12	ZNF207	ARCHS4 Coexpression,31;Enrichr Queries,57;ReMap ChIP-seq,255;GTEx Coexpression,99	DDX3X,FUS,EWSR1,NONO,PGK1
13	PGR	ARCHS4 Coexpression,131;Enrichr Queries,112;ReMap ChIP-seq,123;GTEx Coexpression,90	EBF1,NR2F2,ETS1,VEGFA
14	WT1	Literature ChIP-seq,13;ARCHS4 Coexpression,178;Enrichr Queries,145;GTEx Coexpression,130	FUS,EBF1,NR2F2,ETS1,VEGFA
15	MEOX2	ARCHS4 Coexpression,80;Enrichr Queries,246;GTEx Coexpression,33	EBF1,NR2F2,ETS1
16	ZBTB21	ARCHS4 Coexpression,147;GTEx Coexpression,105	DDX3X
17	C11ORF95	GTEx Coexpression,127	NR2F2
18	PCGF2	ARCHS4 Coexpression,47;ReMap ChIP-seq,215	FUS,NR2F2
19	YY1	Literature ChIP-seq,59;ARCHS4 Coexpression,350;ENCODE ChIP-seq,62;Enrichr Queries,55;ReMap ChIP-seq,153;GTEx Coexpression,124	DDX3X,FUS,EWSR1,NONO,PGK1,NR2F2,ETS1,VEGFA

20	SCX	ARCHS4 Coexpression,134	NR2F2
21	SOX7	ARCHS4 Coexpression,135;Enrichr Queries,132;GTEx Coexpression,155	EFNB2,EBF1,NR2F2,ETS1
22	CREBZF	ARCHS4 Coexpression,45;Enrichr Queries,201;GTEx Coexpression,178	DDX3X,FUS,NONO,ETS1,VEGFA
23	TET1	Literature ChIP-seq,122;ARCHS4 Coexpression,179;GTEx Coexpression,144	NONO,NR2F2
24	HOXD3	ARCHS4 Coexpression,152;Enrichr Queries,268;GTEx Coexpression,25	EBF1,NR2F2,ETS1
25	SON	ARCHS4 Coexpression,284;GTEx Coexpression,14	DDX3X,FUS,NONO
26	OSR2	ARCHS4 Coexpression,206;Enrichr Queries,233;GTEx Coexpression,8	EFNB2,EBF1,NR2F2
27	ZFP69B	ARCHS4 Coexpression,136;GTEx Coexpression,167	NONO,PGK1
28	PAX5	ARCHS4 Coexpression,8;ENCODE ChIP-seq,21;Enrichr Queries,377;ReMap ChIP-seq,162;GTEx Coexpression,196	DDX3X,FUS,PGK1,EBF1,NR2F2,ETS1
29	NR3C1	Literature ChIP-seq,37;ARCHS4 Coexpression,242;ENCODE ChIP-seq,71;Enrichr Queries,291;ReMap ChIP-seq,182;GTEx Coexpression,98	EWSR1,EBF1,NR2F2,ETS1,VEGFA
30	NFATC4	ARCHS4 Coexpression,145;Enrichr Queries,176;GTEx Coexpression,142	EBF1,NR2F2,ETS1,VEGFA
31	KDM5B	Literature ChIP-seq,9;ARCHS4 Coexpression,333;ENCODE ChIP-seq,80;ReMap ChIP-seq,138;GTEx Coexpression,220	EFNB2,DDX3X,EWSR1,FUS,NONO,EBF1,NR2F2
32	GABPA	Literature ChIP-seq,6;ARCHS4 Coexpression,13;ENCODE ChIP-seq,68;Enrichr Queries,709;ReMap ChIP-seq,170;GTEx Coexpression,4	EFNB2,DDX3X,NONO,EBF1,PGK1,NR2F2,ETS1,VEGFA
33	ZBED3	ARCHS4 Coexpression,88;Enrichr Queries,331;GTEx Coexpression,70	EFNB2,FUS,EBF1,ETS1,VEGFA
34	ZNF395	ARCHS4 Coexpression,17;Enrichr Queries,398;GTEx Coexpression,78	NONO,PGK1,ETS1,VEGFA
35	TFAP2A	Literature ChIP-seq,78;ARCHS4 Coexpression,486;ENCODE ChIP-seq,98;Enrichr Queries,32;ReMap ChIP-seq,16;GTEx Coexpression,289	EFNB2,FUS,EBF1,NR2F2,ETS1,VEGFA
36	NFYA	ARCHS4 Coexpression,7;ENCODE ChIP-seq,83;Enrichr Queries,1;ReMap ChIP-seq,258;GTEx Coexpression,491	EFNB2,DDX3X,FUS,NONO,PGK1,NR2F2,ETS1,VEGFA
37	HOXC6	ARCHS4 Coexpression,46;Enrichr Queries,393;GTEx Coexpression,88	EFNB2,FUS,EBF1,NR2F2
38	EBF3	ARCHS4 Coexpression,268;Enrichr Queries,138;GTEx Coexpression,122	EFNB2,EBF1,NR2F2,ETS1
39	FOXO1	Literature ChIP-seq,57;ARCHS4 Coexpression,151;Enrichr Queries,142;ReMap ChIP-seq,80;GTEx Coexpression,454	EFNB2,EBF1,ETS1,VEGFA
40	JUN	Literature ChIP-seq,67;ARCHS4 Coexpression,112;ENCODE ChIP-seq,55;Enrichr Queries,628;ReMap ChIP-seq,187;GTEx Coexpression,16	EFNB2,EWSR1,EBF1,PGK1,NR2F2,ETS1,VEGFA
41	NFATC1	ARCHS4 Coexpression,345;ENCODE ChIP-seq,41;Enrichr Queries,139;ReMap ChIP-seq,38;GTEx Coexpression,332	FUS,EWSR1,EBF1,NR2F2,ETS1,VEGFA
42	ZNF384	ARCHS4 Coexpression,199;ENCODE ChIP-seq,8;Enrichr Queries,445;ReMap ChIP-seq,112;GTEx Coexpression,137	FUS,EWSR1,NONO,EBF1,ETS1,VEGFA
43	SP3	ARCHS4 Coexpression,150;Enrichr Queries,368;GTEx Coexpression,30	DDX3X,EBF1,NR2F2,ETS1
44	PAX8	ARCHS4 Coexpression,133;Enrichr Queries,199;ReMap ChIP-seq,207;GTEx Coexpression,195	EBF1,NR2F2,ETS1,VEGFA
45	EBF2	ARCHS4 Coexpression,327;GTEx Coexpression,40	EBF1,NR2F2
46	MSX1	ARCHS4 Coexpression,347;Enrichr Queries,153;GTEx Coexpression,54	EFNB2,EBF1,NR2F2,ETS1
47	ZNF888	GTEx Coexpression,189	NONO
48	SETBP1	ARCHS4 Coexpression,291;Enrichr Queries,196;GTEx Coexpression,87	EFNB2,EBF1,NR2F2,ETS1
49	AKAP8	ARCHS4 Coexpression,57;Enrichr Queries,56;GTEx Coexpression,468	DDX3X,FUS,EWSR1,NONO,ETS1
50	HOXC5	ARCHS4 Coexpression,318;Enrichr Queries,265;GTEx Coexpression,9	FUS,EBF1,NR2F2,ETS1
51	TFAP2C	Literature ChIP-seq,95;ARCHS4 Coexpression,399;ENCODE ChIP-seq,81;Enrichr Queries,126;ReMap ChIP-seq,104;GTEx Coexpression,386	EFNB2,NR2F2,ETS1,VEGFA
52	CTCF	Literature ChIP-seq,127;ARCHS4 Coexpression,63;ENCODE ChIP-seq,23;Enrichr Queries,45;ReMap ChIP-seq,139;GTEx Coexpression,805	DDX3X,FUS,EWSR1,NONO,ETS1,VEGFA
53	AHR	Literature ChIP-seq,29;ARCHS4 Coexpression,293;Enrichr Queries,363;ReMap ChIP-seq,111;GTEx Coexpression,206	EFNB2,DDX3X,EBF1,NR2F2,ETS1,VEGFA
54	GLI1	Literature ChIP-seq,160;ARCHS4 Coexpression,264;Enrichr Queries,283;GTEx Coexpression,95	FUS,EBF1,NR2F2,ETS1,VEGFA
55	ZBTB14	ARCHS4 Coexpression,81;GTEx Coexpression,324	ETS1
56	SHOX2	ARCHS4 Coexpression,364;Enrichr Queries,231;GTEx Coexpression,18	EFNB2,EBF1,NR2F2
57	ZNF655	ARCHS4 Coexpression,185;Enrichr Queries,313;GTEx Coexpression,117	DDX3X,NONO,ETS1,VEGFA
58	TCF3	Literature ChIP-seq,20;ARCHS4 Coexpression,55;ENCODE ChIP-seq,44;Enrichr Queries,663;ReMap ChIP-seq,264;GTEx Coexpression,203	DDX3X,EWSR1,FUS,NONO,NR2F2,ETS1,VEGFA
59	ZFX	Literature ChIP-seq,94;ARCHS4 Coexpression,82;Enrichr Queries,462;ReMap ChIP-seq,231;GTEx Coexpression,191	DDX3X,EWSR1,NONO,NR2F2,ETS1
60	EN2	ARCHS4 Coexpression,60;Enrichr Queries,65;GTEx Coexpression,512	EFNB2,EBF1,NR2F2,VEGFA
61	ZBTB2	ARCHS4 Coexpression,14;Enrichr Queries,403;GTEx Coexpression,223	EFNB2,DDX3X,ETS1,VEGFA
62	FLI1	Literature ChIP-seq,103;ARCHS4 Coexpression,224;ENCODE ChIP-seq,94;Enrichr Queries,581;ReMap ChIP-seq,165;GTEx Coexpression,114	EFNB2,NONO,EBF1,ETS1
63	BCL11B	Literature ChIP-seq,162;ARCHS4 Coexpression,172;Enrichr Queries,154;GTEx Coexpression,383	EFNB2,EBF1,NR2F2,ETS1
64	ELF1	Literature ChIP-seq,91;ARCHS4 Coexpression,254;ENCODE ChIP-seq,45;ReMap ChIP-seq,188;GTEx Coexpression,517	EWSR1,NONO,PGK1,ETS1
65	HES1	ARCHS4 Coexpression,203;Enrichr Queries,114;ReMap ChIP-seq,289;GTEx Coexpression,277	EFNB2,NR2F2,ETS1,VEGFA
66	IRF4	ARCHS4 Coexpression,314;ENCODE ChIP-seq,47;Enrichr Queries,463;ReMap ChIP-seq,137;GTEx Coexpression,151	DDX3X,FUS,PGK1,EBF1,ETS1,VEGFA
67	BHLHE23	ARCHS4 Coexpression,259;GTEx Coexpression,188	FUS,PGK1
68	FOXP2	Literature ChIP-seq,38;ARCHS4 Coexpression,260;ENCODE ChIP-seq,114;Enrichr Queries,326;ReMap ChIP-seq,199;GTEx Coexpression,405	EFNB2,EBF1,PGK1,NR2F2,ETS1
69	NFATC2	ARCHS4 Coexpression,193;Enrichr Queries,327;GTEx Coexpression,156	EBF1,ETS1,VEGFA
70	ZNF75A	ARCHS4 Coexpression,432;Enrichr Queries,136;GTEx Coexpression,109	FUS,NR2F2,ETS1,VEGFA
71	LHX1	ARCHS4 Coexpression,227;Enrichr Queries,228	EFNB2,EBF1,NR2F2
72	HOXA9	ARCHS4 Coexpression,11;Enrichr Queries,81;ReMap ChIP-seq,8;GTEx Coexpression,813	FUS,EWSR1,EBF1,NR2F2,ETS1,VEGFA
73	ZNF503	ARCHS4 Coexpression,286;Enrichr Queries,300;GTEx Coexpression,113	EFNB2,NR2F2,VEGFA
74	ZNF792	ARCHS4 Coexpression,207;Enrichr Queries,367;GTEx Coexpression,129	EFNB2,ETS1,VEGFA
75	HOXC4	ARCHS4 Coexpression,245;Enrichr Queries,411;GTEx Coexpression,52	FUS,EBF1,NR2F2,ETS1
76	KLF7	ARCHS4 Coexpression,294;Enrichr Queries,392;GTEx Coexpression,22	EFNB2,EBF1,ETS1,VEGFA
77	POU2F2	ARCHS4 Coexpression,276;ENCODE ChIP-seq,101;Enrichr Queries,464;ReMap ChIP-seq,108;GTEx Coexpression,237	FUS,EBF1,ETS1,VEGFA

78	HOXB9	ARCHS4 Coexpression,300;Enrichr Queries,111;GTEx Coexpression,301	EFNB2,NONO,NR2F2,ETS1,VEGFA
79	SOX13	ARCHS4 Coexpression,553;Enrichr Queries,163;ReMap ChIP-seq,205;GTEx Coexpression,32	EFNB2,EBF1,NR2F2,ETS1,VEGFA
80	FOXL2	ARCHS4 Coexpression,429;Enrichr Queries,208;GTEx Coexpression,83	EBF1,NR2F2,ETS1
81	HOXB3	ARCHS4 Coexpression,61;Enrichr Queries,92;GTEx Coexpression,567	EFNB2,FUS,EBF1,NR2F2,ETS1
82	GATA3	Literature ChIP-seq,81;ARCHS4 Coexpression,600;ENCODE ChIP-seq,6;Enrichr Queries,17;ReMap ChIP-seq,109;GTEx Coexpression,644	EFNB2,FUS,EWSR1,EBF1,NR2F2,ETS1,VEGFA
83	SP8	ARCHS4 Coexpression,354;Enrichr Queries,85;GTEx Coexpression,291	EFNB2,EBF1,NR2F2,ETS1
84	SP140L	ARCHS4 Coexpression,351;GTEx Coexpression,141	ETS1
85	STAT1	Literature ChIP-seq,139;ARCHS4 Coexpression,339;ENCODE ChIP-seq,26;Enrichr Queries,601;ReMap ChIP-seq,185;GTEx Coexpression,192	EWSR1,PGK1,ETS1,VEGFA
86	MYC	Literature ChIP-seq,26;ARCHS4 Coexpression,341;ENCODE ChIP-seq,2;Enrichr Queries,597;ReMap ChIP-seq,167;GTEx Coexpression,367	DDX3X,EWSR1,FUS,NONO,PGK1,ETS1,VEGFA
87	LHX9	ARCHS4 Coexpression,37;Enrichr Queries,642;GTEx Coexpression,91	EBF1,NR2F2
88	NFAT5	ARCHS4 Coexpression,380;Enrichr Queries,364;GTEx Coexpression,28	DDX3X,NONO,ETS1,VEGFA
89	HOXA6	ARCHS4 Coexpression,138;Enrichr Queries,76;GTEx Coexpression,568	EFNB2,EWSR1,EBF1,NR2F2,ETS1
90	NR5A2	ARCHS4 Coexpression,360;Enrichr Queries,104;ReMap ChIP-seq,57;GTEx Coexpression,522	EFNB2,EBF1,NR2F2,ETS1
91	STAT3	Literature ChIP-seq,7;ARCHS4 Coexpression,325;ENCODE ChIP-seq,70;Enrichr Queries,288;ReMap ChIP-seq,156;GTEx Coexpression,719	EFNB2,DDX3X,FUS,EWSR1,PGK1,EBF1,NR2F2,ETS1,VEGFA
92	GLYR1	ARCHS4 Coexpression,124;ReMap ChIP-seq,15;GTEx Coexpression,652	FUS,EWSR1
93	KLF4	Literature ChIP-seq,23;ARCHS4 Coexpression,468;Enrichr Queries,379;ReMap ChIP-seq,150;GTEx Coexpression,299	EFNB2,FUS,EBF1,NR2F2,ETS1,VEGFA
94	GLI2	ARCHS4 Coexpression,500;Enrichr Queries,242;GTEx Coexpression,72	EBF1,NR2F2,ETS1
95	ZNF217	Literature ChIP-seq,66;ARCHS4 Coexpression,680;ENCODE ChIP-seq,69;Enrichr Queries,256;ReMap ChIP-seq,46;GTEx Coexpression,519	EFNB2,NR2F2,ETS1,VEGFA
96	ZNF468	ARCHS4 Coexpression,95;Enrichr Queries,564;GTEx Coexpression,160	EFNB2,FUS,NONO,ETS1
97	POU4F2	ARCHS4 Coexpression,35;Enrichr Queries,221;ReMap ChIP-seq,22;GTEx Coexpression,820	FUS,EBF1,NR2F2,ETS1
98	HOXB4	Literature ChIP-seq,65;ARCHS4 Coexpression,234;Enrichr Queries,180;GTEx Coexpression,620	EFNB2,FUS,EWSR1,NONO,EBF1,NR2F2,ETS1
99	SREBF2	Literature ChIP-seq,43;ARCHS4 Coexpression,86;ENCODE ChIP-seq,115;Enrichr Queries,784;ReMap ChIP-seq,254;GTEx Coexpression,376	EFNB2,FUS,EWSR1,ETS1,VEGFA
100	ZSCAN25	ARCHS4 Coexpression,292;GTEx Coexpression,261	ETS1
101	ZNF75D	ARCHS4 Coexpression,170;Enrichr Queries,532;GTEx Coexpression,133	NONO,NR2F2,ETS1,VEGFA
102	KLF2	Literature ChIP-seq,158;ARCHS4 Coexpression,189;Enrichr Queries,736;GTEx Coexpression,31	EBF1,NR2F2,ETS1,VEGFA
103	TEAD4	Literature ChIP-seq,82;ARCHS4 Coexpression,558;ENCODE ChIP-seq,14;Enrichr Queries,629;ReMap ChIP-seq,26;GTEx Coexpression,362	DDX3X,FUS,EWSR1,PGK1,EBF1,NR2F2,ETS1,VEGFA
104	KLF11	ARCHS4 Coexpression,501;Enrichr Queries,289;GTEx Coexpression,46	EFNB2,DDX3X,EBF1,ETS1,VEGFA
105	ZKSCAN8	ARCHS4 Coexpression,349;GTEx Coexpression,209	NONO
106	HOXB5	ARCHS4 Coexpression,159;Enrichr Queries,121;GTEx Coexpression,557	EFNB2,NR2F2,ETS1,VEGFA
107	ZBTB33	ARCHS4 Coexpression,22;ENCODE ChIP-seq,33;Enrichr Queries,38;ReMap ChIP-seq,20;GTEx Coexpression,1286	DDX3X,FUS,NONO,PGK1,NR2F2,ETS1
108	PA2G4	ARCHS4 Coexpression,73;GTEx Coexpression,489	FUS,NONO
109	IKZF3	ARCHS4 Coexpression,125;Enrichr Queries,575;GTEx Coexpression,148	PGK1,EBF1,ETS1
110	TCF7	Literature ChIP-seq,101;ARCHS4 Coexpression,140;Enrichr Queries,450;ReMap ChIP-seq,248;GTEx Coexpression,476	EBF1,PGK1,ETS1,VEGFA
111	HNF1B	ARCHS4 Coexpression,512;Enrichr Queries,257;ReMap ChIP-seq,82	NR2F2,ETS1,VEGFA
112	ZNF740	ARCHS4 Coexpression,27;Enrichr Queries,759;ReMap ChIP-seq,269;GTEx Coexpression,85	FUS,NONO,ETS1,VEGFA
113	CDX2	Literature ChIP-seq,4;ARCHS4 Coexpression,703;Enrichr Queries,234;ReMap ChIP-seq,180;GTEx Coexpression,319	EBF1,NR2F2,ETS1,VEGFA
114	PAX9	ARCHS4 Coexpression,431;Enrichr Queries,91;GTEx Coexpression,347	EFNB2,EBF1,NR2F2,ETS1
115	TBX2	ARCHS4 Coexpression,702;Enrichr Queries,46;GTEx Coexpression,123	EFNB2,EBF1,NR2F2,ETS1,VEGFA
116	ONECUT2	ARCHS4 Coexpression,306;Enrichr Queries,116;GTEx Coexpression,449	EFNB2,EBF1,NR2F2,VEGFA
117	TGIF2	ARCHS4 Coexpression,299;Enrichr Queries,537;ReMap ChIP-seq,116;GTEx Coexpression,211	NONO,ETS1,VEGFA
118	BACH2	ARCHS4 Coexpression,255;Enrichr Queries,118;ReMap ChIP-seq,249;GTEx Coexpression,545	EFNB2,EBF1,NR2F2,ETS1
119	HNF4A	Literature ChIP-seq,17;ARCHS4 Coexpression,679;ENCODE ChIP-seq,91;Enrichr Queries,334;ReMap ChIP-seq,133;GTEx Coexpression,501	EWSR1,NONO,NR2F2,ETS1,VEGFA
120	POU3F1	Literature ChIP-seq,109;ARCHS4 Coexpression,319;Enrichr Queries,61;GTEx Coexpression,685	EFNB2,EBF1,NR2F2,ETS1
121	HOXA1	ARCHS4 Coexpression,421;Enrichr Queries,127;GTEx Coexpression,337	EFNB2,EBF1,NR2F2,ETS1
122	HMX3	ARCHS4 Coexpression,244;Enrichr Queries,494;GTEx Coexpression,149	FUS,PGK1,EBF1,NR2F2
123	FOXD3	ARCHS4 Coexpression,538;Enrichr Queries,218;GTEx Coexpression,131	EBF1,NR2F2,ETS1
124	E2F1	Literature ChIP-seq,30;ARCHS4 Coexpression,58;ENCODE ChIP-seq,87;Enrichr Queries,831;ReMap ChIP-seq,140;GTEx Coexpression,635	DDX3X,FUS,EWSR1,NONO,PGK1,NR2F2,ETS1,VEGFA
125	ZNF783	ARCHS4 Coexpression,342;Enrichr Queries,339;GTEx Coexpression,210	EFNB2,FUS,ETS1,VEGFA
126	TBX4	ARCHS4 Coexpression,710;Enrichr Queries,73;GTEx Coexpression,118	EFNB2,EBF1,NR2F2,ETS1
127	TCF7L2	Literature ChIP-seq,142;ARCHS4 Coexpression,1469;ENCODE ChIP-seq,28;Enrichr Queries,24;ReMap ChIP-seq,128;GTEx Coexpression,15	EFNB2,DDX3X,EWSR1,EBF1,NR2F2,ETS1,VEGFA
128	IKZF5	ARCHS4 Coexpression,115;Enrichr Queries,443;GTEx Coexpression,349	DDX3X,ETS1,VEGFA
129	STAT5A	Literature ChIP-seq,132;ARCHS4 Coexpression,712;ENCODE ChIP-seq,66;Enrichr Queries,731;ReMap ChIP-seq,134;GTEx Coexpression,48	FUS,EWSR1,EBF1,ETS1,VEGFA
130	RORA	ARCHS4 Coexpression,120;Enrichr Queries,332;GTEx Coexpression,464	EBF1,ETS1,VEGFA
131	AR	Literature ChIP-seq,27;ARCHS4 Coexpression,101;Enrichr Queries,5;ReMap ChIP-seq,23;GTEx Coexpression,1371	EFNB2,EBF1,PGK1,NR2F2,ETS1,VEGFA
132	PAX6	Literature ChIP-seq,87;ARCHS4 Coexpression,479;Enrichr Queries,9;ReMap ChIP-seq,56;GTEx Coexpression,897	EFNB2,DDX3X,EBF1,NR2F2,ETS1,VEGFA
133	KLF13	ARCHS4 Coexpression,126;Enrichr Queries,435;ReMap ChIP-seq,60;GTEx Coexpression,606	EFNB2,ETS1,VEGFA
134	TCF4	Literature ChIP-seq,104;ARCHS4 Coexpression,1072;Enrichr Queries,34;ReMap ChIP-seq,282;GTEx Coexpression,43	EFNB2,EBF1,NR2F2,ETS1,VEGFA
135	SMAD3	Literature ChIP-seq,96;ARCHS4 Coexpression,956;Enrichr Queries,324;ReMap ChIP-seq,10;GTEx Coexpression,152	EFNB2,NR2F2,ETS1,VEGFA

136	FOXN3	ARCHS4 Coexpression,280;Enrichr Queries,428;GTEx Coexpression,217	EBF1,NR2F2,ETS1
137	TCF7L1	ARCHS4 Coexpression,779;Enrichr Queries,146;GTEx Coexpression,7	EFNB2,EBF1,NR2F2,ETS1,VEGFA
138	SNAI1	ARCHS4 Coexpression,535;Enrichr Queries,295;GTEx Coexpression,102	EFNB2,ETS1,VEGFA
139	IRF1	Literature ChIP-seq,14;ARCHS4 Coexpression,618;ENCODE ChIP-seq,60;Enrichr Queries,520;ReMap ChIP-seq,6;GTEx Coexpression,650	DDX3X,EWSR1,PGK1,NR2F2,ETS1,VEGFA
140	RARG	Literature ChIP-seq,62;ARCHS4 Coexpression,739;Enrichr Queries,164;GTEx Coexpression,281	EFNB2,NR2F2,ETS1,VEGFA
141	GATA2	Literature ChIP-seq,102;ARCHS4 Coexpression,29;ENCODE ChIP-seq,61;Enrichr Queries,44;ReMap ChIP-seq,233;GTEx Coexpression,1408	EFNB2,FUS,EWSR1,EBF1,PGK1,NR2F2,ETS1,VEGFA
142	CEBPD	Literature ChIP-seq,21;ARCHS4 Coexpression,789;ENCODE ChIP-seq,65;ReMap ChIP-seq,151;GTEx Coexpression,544	EFNB2,FUS,NR2F2,VEGFA
143	NRF1	ARCHS4 Coexpression,146;ENCODE ChIP-seq,78;Enrichr Queries,286;ReMap ChIP-seq,161;GTEx Coexpression,901	EWSR1,NONO,ETS1,VEGFA
144	IRX5	ARCHS4 Coexpression,282;Enrichr Queries,47;GTEx Coexpression,616	EFNB2,EBF1,NR2F2,ETS1,VEGFA
145	HOXD13	Literature ChIP-seq,151;ARCHS4 Coexpression,263;Enrichr Queries,210;GTEx Coexpression,639	NONO,EBF1,NR2F2,ETS1
146	NFE2L2	Literature ChIP-seq,5;ARCHS4 Coexpression,527;Enrichr Queries,612;ReMap ChIP-seq,154;GTEx Coexpression,285	EFNB2,NR2F2,ETS1,VEGFA
147	LHX6	ARCHS4 Coexpression,611;Enrichr Queries,279;GTEx Coexpression,60	EBF1,NR2F2,ETS1
148	CREB5	ARCHS4 Coexpression,476;Enrichr Queries,97;GTEx Coexpression,378	EFNB2,EBF1,ETS1,VEGFA
149	FOSL2	ARCHS4 Coexpression,475;ENCODE ChIP-seq,39;Enrichr Queries,739;ReMap ChIP-seq,194;GTEx Coexpression,158	DDX3X,FUS,EWSR1,ETS1,VEGFA
150	KLF5	Literature ChIP-seq,77;ARCHS4 Coexpression,385;Enrichr Queries,365;ReMap ChIP-seq,198;GTEx Coexpression,583	EFNB2,NR2F2,ETS1,VEGFA
151	PAX3	ARCHS4 Coexpression,41;Enrichr Queries,134;GTEx Coexpression,790	EFNB2,NONO,EBF1,NR2F2,ETS1
152	NKX32	ARCHS4 Coexpression,257;GTEx Coexpression,387	NR2F2
153	FOXF2	ARCHS4 Coexpression,272;Enrichr Queries,19;GTEx Coexpression,676	EFNB2,EBF1,NR2F2,ETS1,VEGFA
154	MYB	Literature ChIP-seq,133;ARCHS4 Coexpression,219;ENCODE ChIP-seq,112;Enrichr Queries,684;ReMap ChIP-seq,159;GTEx Coexpression,631	FUS,ETS1,VEGFA
155	FOXP1	Literature ChIP-seq,55;ARCHS4 Coexpression,1098;Enrichr Queries,52;ReMap ChIP-seq,9;GTEx Coexpression,413	EFNB2,EBF1,PGK1,NR2F2,ETS1,VEGFA
156	ZHX2	ARCHS4 Coexpression,346;Enrichr Queries,200;ReMap ChIP-seq,230;GTEx Coexpression,536	EFNB2,NR2F2,ETS1,VEGFA
157	ZNF532	ARCHS4 Coexpression,249;Enrichr Queries,314;GTEx Coexpression,424	EFNB2,NR2F2,ETS1,VEGFA
158	PROX1	ARCHS4 Coexpression,384;Enrichr Queries,123;ReMap ChIP-seq,256;GTEx Coexpression,554	EFNB2,EBF1,NR2F2,ETS1
159	GSC	ARCHS4 Coexpression,374;Enrichr Queries,502;GTEx Coexpression,112	EBF1,NR2F2
160	CUX1	Literature ChIP-seq,119;ARCHS4 Coexpression,574;ENCODE ChIP-seq,86;Enrichr Queries,425;ReMap ChIP-seq,24;GTEx Coexpression,751	FUS,NR2F2,ETS1,VEGFA
161	PDX1	Literature ChIP-seq,8;ARCHS4 Coexpression,458;Enrichr Queries,488;ReMap ChIP-seq,144;GTEx Coexpression,552	DDX3X,FUS,NONO,EBF1,NR2F2
162	CSRNP2	ARCHS4 Coexpression,210;GTEx Coexpression,450	EFNB2
163	ANHX	ARCHS4 Coexpression,412;GTEx Coexpression,250	
164	CBX2	ARCHS4 Coexpression,77;ENCODE ChIP-seq,99;Enrichr Queries,1109;ReMap ChIP-seq,53;GTEx Coexpression,320	EFNB2,FUS,NONO,EBF1
165	ARNT	Literature ChIP-seq,48;ARCHS4 Coexpression,407;Enrichr Queries,643;ReMap ChIP-seq,27;GTEx Coexpression,543	EBF1,NR2F2,ETS1,VEGFA
166	PBX3	ARCHS4 Coexpression,248;ENCODE ChIP-seq,38;Enrichr Queries,14;ReMap ChIP-seq,224;GTEx Coexpression,1159	EFNB2,FUS,EBF1,PGK1,NR2F2,ETS1,VEGFA
167	MEF2A	Literature ChIP-seq,134;ARCHS4 Coexpression,1116;ENCODE ChIP-seq,107;Enrichr Queries,183;ReMap ChIP-seq,251;GTEx Coexpression,233	DDX3X,NR2F2,ETS1,VEGFA
168	TP63	Literature ChIP-seq,53;ARCHS4 Coexpression,828;Enrichr Queries,147;ReMap ChIP-seq,206;GTEx Coexpression,453	EFNB2,DDX3X,EBF1,NR2F2,ETS1,VEGFA
169	ELK3	Literature ChIP-seq,47;ARCHS4 Coexpression,628;Enrichr Queries,672;GTEx Coexpression,6	EFNB2,DDX3X,FUS,NONO,EBF1,ETS1
170	SMAD4	Literature ChIP-seq,56;ARCHS4 Coexpression,379;Enrichr Queries,452;ReMap ChIP-seq,11;GTEx Coexpression,796	EFNB2,DDX3X,EBF1,ETS1,VEGFA
171	NR2F1	ARCHS4 Coexpression,16;Enrichr Queries,122;ReMap ChIP-seq,227;GTEx Coexpression,993	EFNB2,EBF1,NR2F2,ETS1
172	SIX5	ARCHS4 Coexpression,698;ENCODE ChIP-seq,89;Enrichr Queries,620;ReMap ChIP-seq,219;GTEx Coexpression,73	EWSR1,NR2F2,VEGFA
173	GCM1	ARCHS4 Coexpression,440;Enrichr Queries,236;GTEx Coexpression,348	NR2F2,ETS1,VEGFA
174	ZNF618	ARCHS4 Coexpression,38;Enrichr Queries,920;GTEx Coexpression,71	EFNB2,NR2F2,ETS1
175	HAND1	ARCHS4 Coexpression,269;Enrichr Queries,533;GTEx Coexpression,228	EFNB2,NR2F2
176	DNMT1	ARCHS4 Coexpression,638;GTEx Coexpression,49	FUS,EWSR1
177	SP1	ARCHS4 Coexpression,632;ENCODE ChIP-seq,17;Enrichr Queries,422;ReMap ChIP-seq,143;GTEx Coexpression,506	EFNB2,DDX3X,NONO,PGK1,NR2F2,ETS1,VEGFA
178	AKAP8L	ARCHS4 Coexpression,169;Enrichr Queries,807;GTEx Coexpression,63	EWSR1,FUS,VEGFA
179	ZNF878	ARCHS4 Coexpression,478;GTEx Coexpression,216	PGK1
180	ESR1	Literature ChIP-seq,25;ARCHS4 Coexpression,144;ENCODE ChIP-seq,113;Enrichr Queries,141;ReMap ChIP-seq,75;GTEx Coexpression,1594	EBF1,NR2F2,ETS1,VEGFA
181	PAX2	ARCHS4 Coexpression,328;Enrichr Queries,239;GTEx Coexpression,480	EBF1,NR2F2,ETS1
182	FOXA2	Literature ChIP-seq,118;ARCHS4 Coexpression,575;ENCODE ChIP-seq,40;Enrichr Queries,63;ReMap ChIP-seq,34;GTEx Coexpression,1265	EFNB2,NR2F2,ETS1,VEGFA
183	FOXA1	Literature ChIP-seq,113;ARCHS4 Coexpression,313;ENCODE ChIP-seq,11;Enrichr Queries,296;ReMap ChIP-seq,85;GTEx Coexpression,1285	EFNB2,FUS,EWSR1,NR2F2,ETS1
184	HMX2	ARCHS4 Coexpression,343;Enrichr Queries,496;GTEx Coexpression,214	FUS,PGK1,EBF1,NR2F2
185	MYBL2	Literature ChIP-seq,108;ARCHS4 Coexpression,67;ENCODE ChIP-seq,103;Enrichr Queries,1216;ReMap ChIP-seq,202;GTEx Coexpression,416	FUS,NONO
186	BRF2	ARCHS4 Coexpression,781;ReMap ChIP-seq,13;GTEx Coexpression,265	EFNB2,FUS
187	NR1H3	Literature ChIP-seq,141;ARCHS4 Coexpression,589;Enrichr Queries,810;ReMap ChIP-seq,204;GTEx Coexpression,27	EBF1,ETS1,VEGFA
188	PPARG	Literature ChIP-seq,10;ARCHS4 Coexpression,1495;Enrichr Queries,178;ReMap ChIP-seq,86;GTEx Coexpression,10	EFNB2,EBF1,ETS1,VEGFA

189	RARA	Literature ChIP-seq,69;ARCHS4 Coexpression,598;Enrichr Queries,341;ReMap ChIP-seq,209;GTEx Coexpression,565	EFNB2,NR2F2,ETS1,VEGFA
190	IKZF1	Literature ChIP-seq,154;ARCHS4 Coexpression,119;ENCODE ChIP-seq,56;Enrichr Queries,1117;ReMap ChIP-seq,131;GTEx Coexpression,566	DDX3X,EBF1,ETS1,VEGFA
191	ZNF263	Literature ChIP-seq,159;ARCHS4 Coexpression,212;ENCODE ChIP-seq,25;Enrichr Queries,449;ReMap ChIP-seq,210;GTEx Coexpression,1090	FUS,EBF1,ETS1,VEGFA
192	ZNF846	ARCHS4 Coexpression,358;Enrichr Queries,366;GTEx Coexpression,352	NR2F2,ETS1,VEGFA
193	CREB1	Literature ChIP-seq,52;ARCHS4 Coexpression,1427;ENCODE ChIP-seq,73;Enrichr Queries,382;ReMap ChIP-seq,158;GTEx Coexpression,66	EFNB2,DDX3X,NONO,PGK1,ETS1,VEGFA
194	HES3	ARCHS4 Coexpression,42;Enrichr Queries,649;GTEx Coexpression,389	FUS,NONO,EBF1,NR2F2
195	SALL4	Literature ChIP-seq,12;ARCHS4 Coexpression,390;Enrichr Queries,931;GTEx Coexpression,108	EFNB2,DDX3X,PGK1,NR2F2,VEGFA
196	NHLH1	ARCHS4 Coexpression,228;Enrichr Queries,591;GTEx Coexpression,264	EBF1,NR2F2
197	DUX4	ARCHS4 Coexpression,448;ReMap ChIP-seq,276	
198	GATA1	Literature ChIP-seq,58;ARCHS4 Coexpression,542;ENCODE ChIP-seq,19;Enrichr Queries,860;ReMap ChIP-seq,122;GTEx Coexpression,576	EWSR1,NONO,EBF1,PGK1,ETS1,VEGFA
199	ESRRB	Literature ChIP-seq,129;ARCHS4 Coexpression,807;Enrichr Queries,96;GTEx Coexpression,420	EBF1,NR2F2,ETS1,VEGFA
200	ZIC2	ARCHS4 Coexpression,435;Enrichr Queries,415;GTEx Coexpression,241	EFNB2,EBF1,NR2F2
201	SP2	ARCHS4 Coexpression,657;ENCODE ChIP-seq,35;Enrichr Queries,683;ReMap ChIP-seq,190;GTEx Coexpression,259	EFNB2,PGK1,ETS1,VEGFA
202	RXRA	Literature ChIP-seq,105;ARCHS4 Coexpression,445;ENCODE ChIP-seq,111;Enrichr Queries,451;ReMap ChIP-seq,102;GTEx Coexpression,982	EWSR1,NR2F2,ETS1,VEGFA
203	ZNF697	ARCHS4 Coexpression,652;Enrichr Queries,356;GTEx Coexpression,92	EFNB2,ETS1,VEGFA
204	HOXB1	ARCHS4 Coexpression,6;Enrichr Queries,264;GTEx Coexpression,833	EFNB2,EWSR1,NONO,EBF1,NR2F2,ETS1
205	ERG	Literature ChIP-seq,107;ARCHS4 Coexpression,1463;Enrichr Queries,62;ReMap ChIP-seq,196;GTEx Coexpression,12	EFNB2,FUS,EBF1,NR2F2,ETS1
206	ADNP	ARCHS4 Coexpression,947;Enrichr Queries,420;ReMap ChIP-seq,49;GTEx Coexpression,58	DDX3X,EWSR1,FUS,NONO,VEGFA
207	TAL1	Literature ChIP-seq,35;ARCHS4 Coexpression,1342;ENCODE ChIP-seq,58;Enrichr Queries,406;ReMap ChIP-seq,157;GTEx Coexpression,218	EFNB2,FUS,EBF1,NR2F2,ETS1,VEGFA
208	ELK4	ARCHS4 Coexpression,156;ENCODE ChIP-seq,54;Enrichr Queries,757;ReMap ChIP-seq,69;GTEx Coexpression,817	DDX3X,NONO,PGK1,ETS1,VEGFA
209	PRDM1	ARCHS4 Coexpression,915;ENCODE ChIP-seq,18;Enrichr Queries,298;ReMap ChIP-seq,66;GTEx Coexpression,556	DDX3X,NONO,EBF1,PGK1,NR2F2,ETS1,VEGFA
210	EPAS1	ARCHS4 Coexpression,1224;Enrichr Queries,125;ReMap ChIP-seq,91;GTEx Coexpression,47	EFNB2,NR2F2,ETS1,VEGFA
211	E2F7	Literature ChIP-seq,161;ARCHS4 Coexpression,148;Enrichr Queries,1256;ReMap ChIP-seq,4;GTEx Coexpression,292	EWSR1,NONO,NR2F2
212	MEIS1	Literature ChIP-seq,33;ARCHS4 Coexpression,1227;Enrichr Queries,4;ReMap ChIP-seq,105;GTEx Coexpression,497	EFNB2,EBF1,PGK1,NR2F2,ETS1,VEGFA
213	FOXG1	ARCHS4 Coexpression,122;Enrichr Queries,280;GTEx Coexpression,718	EFNB2,EBF1,NR2F2
214	EGR1	Literature ChIP-seq,72;ARCHS4 Coexpression,1232;ENCODE ChIP-seq,20;Enrichr Queries,655;ReMap ChIP-seq,260;GTEx Coexpression,5	EFNB2,DDX3X,FUS,EWSR1,PGK1,EBF1,NR2F2,ETS1,VEGFA
215	DLX4	Literature ChIP-seq,11;ARCHS4 Coexpression,447;Enrichr Queries,526;GTEx Coexpression,516	EBF1,NR2F2,VEGFA
216	GRHL2	Literature ChIP-seq,89;ARCHS4 Coexpression,550;Enrichr Queries,703;ReMap ChIP-seq,213;GTEx Coexpression,323	EFNB2,NR2F2
217	ZNF248	ARCHS4 Coexpression,388;Enrichr Queries,306;GTEx Coexpression,433	EFNB2,NR2F2,ETS1
218	STAT6	Literature ChIP-seq,145;ARCHS4 Coexpression,221;Enrichr Queries,744;GTEx Coexpression,401	ETS1,VEGFA
219	SALL2	ARCHS4 Coexpression,214;Enrichr Queries,622;GTEx Coexpression,298	EFNB2,NONO,NR2F2
220	LEF1	ARCHS4 Coexpression,182;Enrichr Queries,3;ReMap ChIP-seq,286;GTEx Coexpression,1053	EFNB2,EBF1,NR2F2,ETS1,VEGFA
221	E2F4	Literature ChIP-seq,40;ARCHS4 Coexpression,208;ENCODE ChIP-seq,22;Enrichr Queries,423;ReMap ChIP-seq,172;GTEx Coexpression,1427	FUS,EWSR1,NONO,ETS1,VEGFA
222	ZNF521	ARCHS4 Coexpression,615;Enrichr Queries,86;GTEx Coexpression,445	EFNB2,EBF1,NR2F2,ETS1
223	MNT	ARCHS4 Coexpression,774;Enrichr Queries,444;ReMap ChIP-seq,58;GTEx Coexpression,254	EFNB2,NR2F2,ETS1,VEGFA
224	KDM2B	ARCHS4 Coexpression,274;GTEx Coexpression,492	FUS
225	ZFAT	ARCHS4 Coexpression,585;Enrichr Queries,457;GTEx Coexpression,107	PGK1,NR2F2,ETS1,VEGFA
226	SOX21	ARCHS4 Coexpression,336;Enrichr Queries,307;GTEx Coexpression,507	EFNB2,NR2F2,VEGFA
227	CENPA	ARCHS4 Coexpression,607;GTEx Coexpression,166	PGK1
228	FBXL19	ARCHS4 Coexpression,114;GTEx Coexpression,659	FUS
229	MITF	Literature ChIP-seq,64;ARCHS4 Coexpression,968;Enrichr Queries,87;ReMap ChIP-seq,223;GTEx Coexpression,594	DDX3X,FUS,EWSR1,EBF1,NR2F2,ETS1,VEGFA
230	TEAD2	ARCHS4 Coexpression,48;Enrichr Queries,646;ReMap ChIP-seq,266;GTEx Coexpression,591	EFNB2,FUS,NONO,NR2F2
231	PIN1	ARCHS4 Coexpression,452;GTEx Coexpression,327	
232	CEBPG	ARCHS4 Coexpression,353;Enrichr Queries,558;ReMap ChIP-seq,44;GTEx Coexpression,604	NONO,NR2F2,ETS1,VEGFA
233	CASZ1	ARCHS4 Coexpression,660;Enrichr Queries,54;GTEx Coexpression,456	EFNB2,EBF1,NR2F2,ETS1,VEGFA
234	BAZ2A	ARCHS4 Coexpression,222;Enrichr Queries,847;GTEx Coexpression,103	DDX3X,EWSR1,FUS,VEGFA
235	ESR2	Literature ChIP-seq,144;ARCHS4 Coexpression,1363;Enrichr Queries,345;ReMap ChIP-seq,45;GTEx Coexpression,59	NONO,EBF1,NR2F2,ETS1,VEGFA
236	FOXM1	Literature ChIP-seq,18;ARCHS4 Coexpression,39;ENCODE ChIP-seq,24;Enrichr Queries,1252;ReMap ChIP-seq,174;GTEx Coexpression,841	EFNB2,DDX3X,FUS,NONO,NR2F2,ETS1
237	EOMES	Literature ChIP-seq,28;ARCHS4 Coexpression,815;Enrichr Queries,344;ReMap ChIP-seq,62;GTEx Coexpression,710	EFNB2,EWSR1,EBF1,NR2F2,ETS1
238	BCL11A	ARCHS4 Coexpression,1217;ENCODE ChIP-seq,63;Enrichr Queries,35;ReMap ChIP-seq,113;GTEx Coexpression,537	EFNB2,DDX3X,FUS,EBF1,NR2F2,ETS1,VEGFA
239	RUNX1	Literature ChIP-seq,15;ARCHS4 Coexpression,521;Enrichr Queries,599;ReMap ChIP-seq,169;GTEx Coexpression,662	DDX3X,FUS,ETS1,VEGFA
240	FOXD2	ARCHS4 Coexpression,787;Enrichr Queries,174;GTEx Coexpression,219	EFNB2,EBF1,NR2F2,ETS1
241	NKRF	ARCHS4 Coexpression,334;Enrichr Queries,173;GTEx Coexpression,674	DDX3X,FUS,NONO,PGK1
242	THAP1	ARCHS4 Coexpression,845;ENCODE ChIP-seq,100;Enrichr Queries,30;ReMap ChIP-seq,272;GTEx Coexpression,727	EFNB2,DDX3X,NR2F2,ETS1,VEGFA
243	OTX1	ARCHS4 Coexpression,66;Enrichr Queries,215;GTEx Coexpression,904	EFNB2,EBF1,NR2F2

244	IRX3	ARCHS4 Coexpression,295;Enrichr Queries,187;GTEx Coexpression,705	EFNB2,EBF1,NR2F2,VEGFA
245	MEF2B	ARCHS4 Coexpression,215;Enrichr Queries,353;ReMap ChIP-seq,146;GTEx Coexpression,870	EBF1,ETS1,VEGFA
246	SRCAP	ARCHS4 Coexpression,230;Enrichr Queries,865;GTEx Coexpression,93	DDX3X,EWSR1,FUS,VEGFA
247	ZNF608	ARCHS4 Coexpression,270;Enrichr Queries,94;GTEx Coexpression,826	EFNB2,EBF1,NR2F2,ETS1
248	ZNF703	ARCHS4 Coexpression,369;Enrichr Queries,144;GTEx Coexpression,678	EFNB2,NR2F2,ETS1,VEGFA
249	ZNF12	ARCHS4 Coexpression,163;Enrichr Queries,922;GTEx Coexpression,110	DDX3X,NONO,VEGFA
250	SOX4	ARCHS4 Coexpression,1017;Enrichr Queries,158;GTEx Coexpression,21	EFNB2,NONO,NR2F2,ETS1,VEGFA
251	TP53	Literature ChIP-seq,39;ARCHS4 Coexpression,53;Enrichr Queries,563;ReMap ChIP-seq,229;GTEx Coexpression,1111	FUS,NONO,EBF1,PGK1,ETS1,VEGFA
252	KLF3	ARCHS4 Coexpression,160;Enrichr Queries,799;ReMap ChIP-seq,18;GTEx Coexpression,619	NR2F2,ETS1,VEGFA
253	HSF1	Literature ChIP-seq,136;ARCHS4 Coexpression,1321;ENCODE ChIP-seq,4;ReMap ChIP-seq,97;GTEx Coexpression,438	EWSR1,PGK1
254	HOXC9	Literature ChIP-seq,120;ARCHS4 Coexpression,462;Enrichr Queries,284;GTEx Coexpression,732	EBF1,NR2F2,ETS1
255	SOX17	Literature ChIP-seq,31;ARCHS4 Coexpression,1484;Enrichr Queries,49;GTEx Coexpression,34	EFNB2,DDX3X,EBF1,NR2F2,ETS1,VEGFA
256	HIC1	ARCHS4 Coexpression,1161;Enrichr Queries,22;GTEx Coexpression,19	EFNB2,EBF1,NR2F2,ETS1,VEGFA
257	SRY	Literature ChIP-seq,117;ARCHS4 Coexpression,609;Enrichr Queries,539;GTEx Coexpression,338	EBF1,NR2F2,ETS1
258	HOXA2	Literature ChIP-seq,163;ARCHS4 Coexpression,1;Enrichr Queries,98;GTEx Coexpression,1343	EFNB2,EWSR1,NONO,EBF1,NR2F2,ETS1
259	SP100	ARCHS4 Coexpression,18;Enrichr Queries,1031;GTEx Coexpression,161	NONO,ETS1
260	HIF1A	Literature ChIP-seq,146;ARCHS4 Coexpression,143;Enrichr Queries,170;ReMap ChIP-seq,101;GTEx Coexpression,1464	DDX3X,PGK1,ETS1,VEGFA
261	RBPJ	Literature ChIP-seq,112;ARCHS4 Coexpression,1464;Enrichr Queries,186;ReMap ChIP-seq,179;GTEx Coexpression,86	DDX3X,NONO,EBF1,PGK1,ETS1,VEGFA
262	ZBTB42	ARCHS4 Coexpression,525;GTEx Coexpression,287	
263	ZXDA	ARCHS4 Coexpression,213;Enrichr Queries,671;GTEx Coexpression,336	NR2F2,ETS1,VEGFA
264	SPI1	Literature ChIP-seq,73;ARCHS4 Coexpression,667;ENCODE ChIP-seq,32;Enrichr Queries,1143;ReMap ChIP-seq,193;GTEx Coexpression,333	DDX3X,PGK1,NR2F2,ETS1
265	CEBPB	Literature ChIP-seq,61;ARCHS4 Coexpression,870;ENCODE ChIP-seq,34;Enrichr Queries,573;ReMap ChIP-seq,95;GTEx Coexpression,809	EWSR1,PGK1,NR2F2,ETS1,VEGFA
266	HOXB6	ARCHS4 Coexpression,283;Enrichr Queries,106;GTEx Coexpression,832	EFNB2,EBF1,NR2F2,ETS1
267	NR3C2	ARCHS4 Coexpression,409;Enrichr Queries,545;GTEx Coexpression,269	EFNB2,ETS1
268	SMAD1	Literature ChIP-seq,74;ARCHS4 Coexpression,1013;Enrichr Queries,133;ReMap ChIP-seq,32;GTEx Coexpression,787	EFNB2,FUS,NONO,NR2F2,ETS1,VEGFA
269	USF1	ARCHS4 Coexpression,442;ENCODE ChIP-seq,49;Enrichr Queries,787;ReMap ChIP-seq,212;GTEx Coexpression,550	DDX3X,EWSR1,ETS1,VEGFA
270	GLIS3	ARCHS4 Coexpression,1053;Enrichr Queries,28;GTEx Coexpression,146	EFNB2,EBF1,NR2F2,ETS1,VEGFA
271	POU5F1	Literature ChIP-seq,22;ARCHS4 Coexpression,296;Enrichr Queries,685;ReMap ChIP-seq,41;GTEx Coexpression,1001	FUS,NONO,NR2F2,ETS1,VEGFA
272	CENPS	ARCHS4 Coexpression,420;GTEx Coexpression,399	
273	ZBTB40	ARCHS4 Coexpression,52;Enrichr Queries,987;GTEx Coexpression,190	FUS,NONO,ETS1,VEGFA
274	ZIC3	Literature ChIP-seq,147;ARCHS4 Coexpression,240;Enrichr Queries,644;GTEx Coexpression,609	NONO,EBF1,NR2F2
275	ONECUT1	ARCHS4 Coexpression,674;Enrichr Queries,253;ReMap ChIP-seq,39;GTEx Coexpression,675	EFNB2,EBF1,NR2F2,ETS1
276	MYOD1	ARCHS4 Coexpression,436;ENCODE ChIP-seq,64;Enrichr Queries,77;ReMap ChIP-seq,232;GTEx Coexpression,1243	EFNB2,EBF1,NR2F2,ETS1,VEGFA
277	IKZF2	ARCHS4 Coexpression,225;Enrichr Queries,841;GTEx Coexpression,170	PGK1,ETS1,VEGFA
278	ZBTB16	ARCHS4 Coexpression,529;Enrichr Queries,88;ReMap ChIP-seq,12;GTEx Coexpression,1023	EFNB2,DDX3X,EBF1,NR2F2,ETS1,VEGFA
279	MYCN	Literature ChIP-seq,115;ARCHS4 Coexpression,352;Enrichr Queries,105;ReMap ChIP-seq,216;GTEx Coexpression,1277	EFNB2,DDX3X,NONO,NR2F2,ETS1,VEGFA
280	IRX2	ARCHS4 Coexpression,683;Enrichr Queries,250;GTEx Coexpression,307	EFNB2,EBF1,NR2F2
281	CPEB1	ARCHS4 Coexpression,520;GTEx Coexpression,309	
282	ELK1	Literature ChIP-seq,88;ARCHS4 Coexpression,1328;ENCODE ChIP-seq,85;ReMap ChIP-seq,166;GTEx Coexpression,408	EWSR1,EBF1
283	SATB1	ARCHS4 Coexpression,258;Enrichr Queries,446;ReMap ChIP-seq,43;GTEx Coexpression,917	NONO,NR2F2,ETS1,VEGFA
284	ZNF584	ARCHS4 Coexpression,595;Enrichr Queries,466;ReMap ChIP-seq,274;GTEx Coexpression,330	EFNB2,ETS1,VEGFA
285	HSFY2	ARCHS4 Coexpression,389;GTEx Coexpression,448	
286	HLX	ARCHS4 Coexpression,800;Enrichr Queries,39;GTEx Coexpression,417	EFNB2,EBF1,NR2F2,ETS1,VEGFA
287	NHLH2	ARCHS4 Coexpression,304;Enrichr Queries,577;GTEx Coexpression,375	EBF1,NR2F2
288	ZNF512	ARCHS4 Coexpression,128;Enrichr Queries,997;GTEx Coexpression,135	NONO,NR2F2,ETS1
289	ZNF304	ARCHS4 Coexpression,321;Enrichr Queries,571;GTEx Coexpression,370	EFNB2,DDX3X,VEGFA
290	MAFB	ARCHS4 Coexpression,799;Enrichr Queries,371;ReMap ChIP-seq,132;GTEx Coexpression,384	EFNB2,ETS1,VEGFA
291	ZNF324B	ARCHS4 Coexpression,443;GTEx Coexpression,402	
292	IRF3	ARCHS4 Coexpression,173;ENCODE ChIP-seq,9;Enrichr Queries,410;ReMap ChIP-seq,14;GTEx Coexpression,1508	EFNB2,EWSR1,NONO,PGK1,ETS1,VEGFA
293	KLF14	ARCHS4 Coexpression,530;Enrichr Queries,318	EBF1,NR2F2,ETS1
294	GTF2B	ARCHS4 Coexpression,1140;ENCODE ChIP-seq,93;ReMap ChIP-seq,197;GTEx Coexpression,266	
295	ZBTB5	ARCHS4 Coexpression,10;Enrichr Queries,592;GTEx Coexpression,670	EFNB2,FUS,NONO,VEGFA
296	HMG20A	ARCHS4 Coexpression,344;Enrichr Queries,687;GTEx Coexpression,243	NONO,NR2F2,ETS1
297	ARNTL	ARCHS4 Coexpression,116;Enrichr Queries,275;ReMap ChIP-seq,221;GTEx Coexpression,1091	EFNB2,ETS1,VEGFA
298	PRDM5	Literature ChIP-seq,16;ARCHS4 Coexpression,1523;Enrichr Queries,69;GTEx Coexpression,96	DDX3X,NONO,EBF1,NR2F2,ETS1,VEGFA
299	PURA	ARCHS4 Coexpression,367;GTEx Coexpression,485	
300	TFCP2L1	ARCHS4 Coexpression,664;Enrichr Queries,317;GTEx Coexpression,304	EFNB2,ETS1,VEGFA
301	RFX5	ARCHS4 Coexpression,315;ENCODE ChIP-seq,42;Enrichr Queries,840;ReMap ChIP-seq,164;GTEx Coexpression,786	DDX3X,FUS,ETS1,VEGFA
302	SOX9	Literature ChIP-seq,98;ARCHS4 Coexpression,198;Enrichr Queries,160;ReMap ChIP-seq,235;GTEx Coexpression,1456	EFNB2,NR2F2,ETS1,VEGFA
303	BHLHA15	ARCHS4 Coexpression,392;GTEx Coexpression,467	
304	SRF	Literature ChIP-seq,130;ARCHS4 Coexpression,308;ENCODE ChIP-seq,97;Enrichr Queries,375;ReMap ChIP-seq,214;GTEx Coexpression,1454	EFNB2,FUS,ETS1,VEGFA

305	MYBL1	Literature ChIP-seq,155;ARCHS4 Coexpression,104;Enrichr Queries,952;GTEx Coexpression,509	ETS1
306	ARID5A	ARCHS4 Coexpression,499;GTEx Coexpression,361	
307	ZNF366	ARCHS4 Coexpression,705;Enrichr Queries,557;GTEx Coexpression,29	EBF1,ETS1
308	KLF6	ARCHS4 Coexpression,775;Enrichr Queries,328;ReMap ChIP-seq,25;GTEx Coexpression,595	EFNB2,NR2F2,ETS1,VEGFA
309	SETDB1	Literature ChIP-seq,41;ARCHS4 Coexpression,74;ENCODE ChIP-seq,77;Enrichr Queries,1012;ReMap ChIP-seq,241;GTEx Coexpression,1140	DDX3X,EWSR1,FUS,NONO,EBF1,PGK1,NR2F2
310	GLMP	ARCHS4 Coexpression,541;GTEx Coexpression,325	
311	DZIP1	ARCHS4 Coexpression,469;Enrichr Queries,42;GTEx Coexpression,789	EFNB2,EBF1,NR2F2,ETS1,VEGFA
312	HOXD10	ARCHS4 Coexpression,2;Enrichr Queries,285;GTEx Coexpression,1017	FUS,NONO,EBF1,NR2F2,ETS1
313	TFAP2B	ARCHS4 Coexpression,297;Enrichr Queries,248;GTEx Coexpression,760	EFNB2,EBF1,NR2F2
314	ZFP30	ARCHS4 Coexpression,376;Enrichr Queries,383;GTEx Coexpression,547	EFNB2,NR2F2,VEGFA
315	ZKSCAN1	ARCHS4 Coexpression,581;ENCODE ChIP-seq,88;Enrichr Queries,777;ReMap ChIP-seq,31;GTEx Coexpression,702	EWSR1,NR2F2,VEGFA
316	SCRT2	ARCHS4 Coexpression,130;Enrichr Queries,732;GTEx Coexpression,446	EBF1,NR2F2
317	HIVEP2	ARCHS4 Coexpression,141;Enrichr Queries,319;GTEx Coexpression,851	EFNB2,ETS1,VEGFA
318	BHLHE40	ARCHS4 Coexpression,676;ENCODE ChIP-seq,13;ReMap ChIP-seq,142;GTEx Coexpression,920	DDX3X,FUS,EWSR1,PGK1,VEGFA
319	E2F3	ARCHS4 Coexpression,233;Enrichr Queries,619;GTEx Coexpression,463	FUS,ETS1,VEGFA
320	ZNF423	ARCHS4 Coexpression,194;Enrichr Queries,531;GTEx Coexpression,593	EFNB2,EBF1,NR2F2
321	ERF	ARCHS4 Coexpression,317;Enrichr Queries,718;GTEx Coexpression,284	FUS,ETS1,VEGFA
322	BCL6	Literature ChIP-seq,80;ARCHS4 Coexpression,760;Enrichr Queries,681;ReMap ChIP-seq,203;GTEx Coexpression,478	EBF1,ETS1,VEGFA
323	MTF1	ARCHS4 Coexpression,610;GTEx Coexpression,271	
324	ATF2	ARCHS4 Coexpression,1112;ENCODE ChIP-seq,76;Enrichr Queries,456;ReMap ChIP-seq,126;GTEx Coexpression,447	DDX3X,FUS,EWSR1,ETS1,VEGFA
325	PRDM13	ARCHS4 Coexpression,560;Enrichr Queries,565;GTEx Coexpression,208	PGK1,EBF1,NR2F2
326	ZNF736	ARCHS4 Coexpression,889;GTEx Coexpression,1	FUS,EWSR1,NONO
327	HOXB7	Literature ChIP-seq,121;ARCHS4 Coexpression,940;Enrichr Queries,161;ReMap ChIP-seq,292;GTEx Coexpression,712	EFNB2,EBF1,NR2F2,ETS1
328	ALX4	ARCHS4 Coexpression,3;Enrichr Queries,235;GTEx Coexpression,1103	FUS,NONO,EBF1,NR2F2,ETS1
329	ZNF317	ARCHS4 Coexpression,256;Enrichr Queries,721;GTEx Coexpression,364	DDX3X,EWSR1,VEGFA
330	ZNF274	Literature ChIP-seq,148;ARCHS4 Coexpression,677;ENCODE ChIP-seq,30;Enrichr Queries,785;ReMap ChIP-seq,67;GTEx Coexpression,977	FUS,NONO,ETS1,VEGFA
331	PBX2	ARCHS4 Coexpression,277;Enrichr Queries,426;ReMap ChIP-seq,136;GTEx Coexpression,954	FUS,NR2F2,ETS1,VEGFA
332	RUNX3	ARCHS4 Coexpression,324;ENCODE ChIP-seq,102;Enrichr Queries,849;ReMap ChIP-seq,246;GTEx Coexpression,736	ETS1,VEGFA
333	ZBTB10	ARCHS4 Coexpression,1095;Enrichr Queries,157;GTEx Coexpression,104	EFNB2,EBF1,ETS1,VEGFA
334	MYOG	ARCHS4 Coexpression,842;ENCODE ChIP-seq,7;Enrichr Queries,346;GTEx Coexpression,618	EFNB2,DDX3X,FUS,EWSR1,EBF1,ETS1,VEGFA
335	ETS1	Literature ChIP-seq,126;ARCHS4 Coexpression,1336;ENCODE ChIP-seq,43;Enrichr Queries,162;ReMap ChIP-seq,118;GTEx Coexpression,943	EFNB2,FUS,EBF1,NR2F2,ETS1,VEGFA
336	POU4F3	ARCHS4 Coexpression,795;Enrichr Queries,480;GTEx Coexpression,89	EBF1,NR2F2
337	ARID3A	ARCHS4 Coexpression,630;ENCODE ChIP-seq,57;Enrichr Queries,737;ReMap ChIP-seq,117;GTEx Coexpression,734	FUS,PGK1,NR2F2,ETS1,VEGFA
338	DLX3	ARCHS4 Coexpression,382;Enrichr Queries,723;GTEx Coexpression,260	EFNB2,NR2F2
339	E2F5	ARCHS4 Coexpression,1237;Enrichr Queries,82;ReMap ChIP-seq,280;GTEx Coexpression,225	EFNB2,EBF1,ETS1,VEGFA
340	MEIS2	ARCHS4 Coexpression,204;Enrichr Queries,143;ReMap ChIP-seq,47;GTEx Coexpression,1430	EFNB2,EBF1,NR2F2,ETS1
341	HOXB2	ARCHS4 Coexpression,704;Enrichr Queries,21;GTEx Coexpression,645	EFNB2,EBF1,NR2F2,ETS1,VEGFA
342	ZBED4	ARCHS4 Coexpression,78;Enrichr Queries,835;GTEx Coexpression,458	EFNB2,FUS,NONO,ETS1
343	FOXO3	Literature ChIP-seq,75;ARCHS4 Coexpression,644;Enrichr Queries,390;GTEx Coexpression,721	FUS,PGK1,NR2F2,ETS1,VEGFA
344	ZNF280D	ARCHS4 Coexpression,960;Enrichr Queries,333;GTEx Coexpression,81	NR2F2,ETS1,VEGFA
345	BARHL1	ARCHS4 Coexpression,109;Enrichr Queries,523;GTEx Coexpression,743	EBF1,NR2F2
346	ZNF724	ARCHS4 Coexpression,731;GTEx Coexpression,186	PGK1
347	ZNF25	ARCHS4 Coexpression,678;Enrichr Queries,102;GTEx Coexpression,597	EFNB2,NR2F2,ETS1,VEGFA
348	FOXN2	ARCHS4 Coexpression,12;Enrichr Queries,863;GTEx Coexpression,502	DDX3X,ETS1,VEGFA
349	ZFPM2	ARCHS4 Coexpression,762;Enrichr Queries,90;GTEx Coexpression,526	EFNB2,EBF1,NR2F2,ETS1
350	MYSM1	ARCHS4 Coexpression,180;Enrichr Queries,1015;GTEx Coexpression,183	DDX3X,NONO
351	LYL1	Literature ChIP-seq,83;ARCHS4 Coexpression,386;Enrichr Queries,1124;ReMap ChIP-seq,220;GTEx Coexpression,486	EFNB2,ETS1
352	TSHZ3	ARCHS4 Coexpression,1228;Enrichr Queries,68;GTEx Coexpression,84	EFNB2,EBF1,NR2F2,ETS1
353	RELA	Literature ChIP-seq,97;ARCHS4 Coexpression,944;ENCODE ChIP-seq,15;Enrichr Queries,774;ReMap ChIP-seq,184;GTEx Coexpression,752	DDX3X,FUS,EBF1,PGK1,ETS1,VEGFA
354	HOXD12	ARCHS4 Coexpression,89;Enrichr Queries,487;GTEx Coexpression,810	EBF1,NR2F2
355	UBP1	ARCHS4 Coexpression,288;Enrichr Queries,800;GTEx Coexpression,300	DDX3X,FUS,VEGFA
356	PBX1	Literature ChIP-seq,116;ARCHS4 Coexpression,594;Enrichr Queries,108;GTEx Coexpression,1033	EFNB2,DDX3X,EBF1,NR2F2,ETS1
357	PLSCR1	ARCHS4 Coexpression,756;GTEx Coexpression,172	ETS1
358	ZNF704	ARCHS4 Coexpression,809;Enrichr Queries,269;GTEx Coexpression,314	EFNB2,NR2F2,ETS1
359	BNC2	ARCHS4 Coexpression,1042;Enrichr Queries,207;GTEx Coexpression,143	EBF1,NR2F2,ETS1
360	TBP	Literature ChIP-seq,1;ARCHS4 Coexpression,1371;ENCODE ChIP-seq,29;ReMap ChIP-seq,141;GTEx Coexpression,785	EFNB2,FUS,PGK1,EBF1,NR2F2,ETS1,VEGFA
361	FOXP3	Literature ChIP-seq,36;ARCHS4 Coexpression,127;Enrichr Queries,848;GTEx Coexpression,857	ETS1,VEGFA
362	RUNX2	Literature ChIP-seq,51;ARCHS4 Coexpression,927;Enrichr Queries,23;ReMap ChIP-seq,29;GTEx Coexpression,1306	EFNB2,DDX3X,EBF1,NR2F2,ETS1,VEGFA
363	ETV3L	ARCHS4 Coexpression,562;Enrichr Queries,676;GTEx Coexpression,164	PGK1,EBF1,VEGFA
364	THAP12	ARCHS4 Coexpression,26;GTEx Coexpression,912	DDX3X,ETS1
365	NFYC	ARCHS4 Coexpression,505;Enrichr Queries,53;GTEx Coexpression,849	FUS,EWSR1,PGK1,ETS1,VEGFA
366	GLI3	ARCHS4 Coexpression,289;Enrichr Queries,64;GTEx Coexpression,1054	EFNB2,EBF1,NR2F2,ETS1
367	NFKB1	ARCHS4 Coexpression,873;Enrichr Queries,762;ReMap ChIP-seq,40;GTEx Coexpression,202	PGK1,NR2F2,ETS1,VEGFA
368	ZNF34	ARCHS4 Coexpression,625;Enrichr Queries,645;GTEx Coexpression,138	EFNB2,EBF1,VEGFA

369	GBX2	Literature ChIP-seq,149;ARCHS4 Coexpression,75;Enrichr Queries,71;GTEx Coexpression,1584	EFNB2,NONO,EBF1,NR2F2,ETS1
370	STAT5B	ARCHS4 Coexpression,191;Enrichr Queries,824;ReMap ChIP-seq,163;GTEx Coexpression,706	ETS1,VEGFA
371	LHX4	ARCHS4 Coexpression,153;Enrichr Queries,637;GTEx Coexpression,627	FUS,EBF1,NR2F2
372	VDR	Literature ChIP-seq,86;ARCHS4 Coexpression,684;Enrichr Queries,733;ReMap ChIP-seq,171;GTEx Coexpression,691	FUS,PGK1,ETS1,VEGFA
373	GATAD2B	ARCHS4 Coexpression,266;Enrichr Queries,864;GTEx Coexpression,290	DDX3X,FUS
374	OLIG3	ARCHS4 Coexpression,54;Enrichr Queries,515;GTEx Coexpression,853	EBF1,NR2F2
375	HBP1	ARCHS4 Coexpression,265;Enrichr Queries,647;ReMap ChIP-seq,120;GTEx Coexpression,865	FUS,ETS1,VEGFA
376	ZNF831	ARCHS4 Coexpression,181;Enrichr Queries,876;GTEx Coexpression,366	ETS1
377	ZBTB11	ARCHS4 Coexpression,24;Enrichr Queries,1115;ReMap ChIP-seq,259;GTEx Coexpression,504	DDX3X,ETS1
378	MSX2	ARCHS4 Coexpression,302;Enrichr Queries,175;GTEx Coexpression,950	EFNB2,EBF1,NR2F2,ETS1
379	EGR4	ARCHS4 Coexpression,669;Enrichr Queries,337;GTEx Coexpression,422	EFNB2,EBF1,VEGFA
380	ZNF354B	ARCHS4 Coexpression,363;Enrichr Queries,929;GTEx Coexpression,140	EBF1,VEGFA
381	HOXA3	ARCHS4 Coexpression,290;Enrichr Queries,8;GTEx Coexpression,1136	EFNB2,EBF1,NR2F2,ETS1,VEGFA
382	NR2C2	ARCHS4 Coexpression,511;ENCODE ChIP-seq,67;Enrichr Queries,1056;ReMap ChIP-seq,201;GTEx Coexpression,559	EWSR1,ETS1
383	PITX1	ARCHS4 Coexpression,583;Enrichr Queries,380;GTEx Coexpression,474	EBF1,NR2F2,ETS1
384	NFIA	ARCHS4 Coexpression,184;Enrichr Queries,26;GTEx Coexpression,1228	EFNB2,EBF1,NR2F2,ETS1,VEGFA
385	REST	Literature ChIP-seq,49;ARCHS4 Coexpression,1618;ENCODE ChIP-seq,3;Enrichr Queries,322;ReMap ChIP-seq,176;GTEx Coexpression,715	DDX3X,FUS,EWSR1,EBF1,NR2F2,ETS1,VEGFA
386	NCOA3	ARCHS4 Coexpression,1218;Enrichr Queries,263;ReMap ChIP-seq,218;GTEx Coexpression,227	PGK1,ETS1,VEGFA
387	XBP1	ARCHS4 Coexpression,584;Enrichr Queries,745;ReMap ChIP-seq,243;GTEx Coexpression,356	PGK1,VEGFA
388	EBF1	ARCHS4 Coexpression,424;ENCODE ChIP-seq,12;Enrichr Queries,272;ReMap ChIP-seq,135;GTEx Coexpression,1567	EFNB2,FUS,EWSR1,EBF1,PGK1,NR2F2,ETS1,VEGFA
389	TBXT	ARCHS4 Coexpression,741;Enrichr Queries,59;ReMap ChIP-seq,72;GTEx Coexpression,1069	EFNB2,EBF1,NR2F2,ETS1
390	MAZ	ARCHS4 Coexpression,303;ENCODE ChIP-seq,46;Enrichr Queries,617;ReMap ChIP-seq,149;GTEx Coexpression,1315	FUS,NONO,ETS1,VEGFA
391	LIN28B	ARCHS4 Coexpression,229;Enrichr Queries,297;GTEx Coexpression,937	EFNB2,NONO,EBF1,NR2F2
392	HNF4G	ARCHS4 Coexpression,621;ENCODE ChIP-seq,51;Enrichr Queries,350;ReMap ChIP-seq,200;GTEx Coexpression,1224	EFNB2,NR2F2,ETS1,VEGFA
393	HSFX1	ARCHS4 Coexpression,654;GTEx Coexpression,329	
394	IRX1	ARCHS4 Coexpression,311;Enrichr Queries,194;GTEx Coexpression,970	EFNB2,EBF1,NR2F2,ETS1
395	PURB	ARCHS4 Coexpression,246;GTEx Coexpression,738	FUS
396	ZIC4	ARCHS4 Coexpression,238;Enrichr Queries,510;GTEx Coexpression,729	EBF1,PGK1,NR2F2
397	OVOL2	ARCHS4 Coexpression,482;Enrichr Queries,578;ReMap ChIP-seq,295;GTEx Coexpression,622	EFNB2,NR2F2
398	POU3F2	Literature ChIP-seq,125;ARCHS4 Coexpression,72;Enrichr Queries,259;GTEx Coexpression,1523	EFNB2,EBF1,NR2F2
399	ZNF705E	ARCHS4 Coexpression,813;GTEx Coexpression,179	NONO
400	MEF2C	ARCHS4 Coexpression,1102;ENCODE ChIP-seq,117;Enrichr Queries,66;ReMap ChIP-seq,247;GTEx Coexpression,948	EFNB2,EBF1,NR2F2,ETS1
401	ZNF136	ARCHS4 Coexpression,564;Enrichr Queries,911;GTEx Coexpression,13	NONO,EBF1,ETS1
402	VSX1	ARCHS4 Coexpression,718;Enrichr Queries,525;GTEx Coexpression,245	EBF1,NR2F2
403	ZIC5	ARCHS4 Coexpression,411;Enrichr Queries,311;GTEx Coexpression,767	EFNB2,EBF1,NR2F2
404	HOXD11	ARCHS4 Coexpression,62;Enrichr Queries,512;GTEx Coexpression,916	NONO,EBF1,NR2F2
405	NEUROD1	ARCHS4 Coexpression,844;Enrichr Queries,589;ReMap ChIP-seq,175;GTEx Coexpression,382	EBF1,NR2F2
406	NEUROG2	ARCHS4 Coexpression,1085;Enrichr Queries,216;GTEx Coexpression,193	EFNB2,PGK1,EBF1,NR2F2
407	ZNF117	ARCHS4 Coexpression,545;Enrichr Queries,881;GTEx Coexpression,69	EBF1,VEGFA
408	MNX1	Literature ChIP-seq,123;ARCHS4 Coexpression,494;Enrichr Queries,885;GTEx Coexpression,494	NR2F2
409	SNAI3	ARCHS4 Coexpression,372;Enrichr Queries,715;GTEx Coexpression,410	ETS1,VEGFA
410	HOXC11	ARCHS4 Coexpression,793;Enrichr Queries,543;ReMap ChIP-seq,294;GTEx Coexpression,388	EBF1,NR2F2
411	ZNF559	ARCHS4 Coexpression,721;Enrichr Queries,659;GTEx Coexpression,134	DDX3X,NR2F2,ETS1
412	THAP10	ARCHS4 Coexpression,875;Enrichr Queries,323;GTEx Coexpression,318	EFNB2,NR2F2,VEGFA
413	RFX4	ARCHS4 Coexpression,419;Enrichr Queries,572;GTEx Coexpression,532	EBF1,NR2F2
414	ETV1	ARCHS4 Coexpression,1436;Enrichr Queries,169;ReMap ChIP-seq,181;GTEx Coexpression,246	EFNB2,EBF1,NR2F2,ETS1
415	ZBTB39	ARCHS4 Coexpression,43;Enrichr Queries,801;GTEx Coexpression,681	FUS,NONO,ETS1,VEGFA
416	ZNF66	ARCHS4 Coexpression,836;GTEx Coexpression,182	NONO
417	TET3	ARCHS4 Coexpression,417;ReMap ChIP-seq,296;GTEx Coexpression,814	
418	EGR3	ARCHS4 Coexpression,446;Enrichr Queries,373;GTEx Coexpression,711	EFNB2,EBF1,VEGFA
419	SCML4	ARCHS4 Coexpression,154;Enrichr Queries,854;GTEx Coexpression,523	EBF1,ETS1
420	NFE2	ARCHS4 Coexpression,547;ENCODE ChIP-seq,31;Enrichr Queries,1137;ReMap ChIP-seq,152;GTEx Coexpression,688	DDX3X,FUS,EWSR1,ETS1,VEGFA
421	ZBTB46	ARCHS4 Coexpression,361;Enrichr Queries,197;GTEx Coexpression,980	EFNB2,EBF1,NR2F2,ETS1
422	SIX1	ARCHS4 Coexpression,603;Enrichr Queries,177;GTEx Coexpression,759	EFNB2,EBF1,NR2F2,VEGFA
423	SOX2	Literature ChIP-seq,32;ARCHS4 Coexpression,861;Enrichr Queries,113;ReMap ChIP-seq,33;GTEx Coexpression,1527	EFNB2,NR2F2,ETS1,VEGFA
424	MAF	ARCHS4 Coexpression,1447;Enrichr Queries,11;ReMap ChIP-seq,168;GTEx Coexpression,427	EFNB2,EBF1,NR2F2,ETS1,VEGFA
425	ALX1	ARCHS4 Coexpression,413;Enrichr Queries,74;GTEx Coexpression,1056	EFNB2,EBF1,NR2F2,ETS1
426	ATF4	ARCHS4 Coexpression,226;Enrichr Queries,668;ReMap ChIP-seq,59;GTEx Coexpression,1105	FUS,PGK1,VEGFA
427	ATF3	Literature ChIP-seq,84;ARCHS4 Coexpression,982;ENCODE ChIP-seq,79;Enrichr Queries,751;ReMap ChIP-seq,160;GTEx Coexpression,1040	EFNB2,FUS,ETS1,VEGFA
428	ZEB1	ARCHS4 Coexpression,723;ENCODE ChIP-seq,108;Enrichr Queries,31;ReMap ChIP-seq,114;GTEx Coexpression,1605	EFNB2,EBF1,NR2F2,ETS1,VEGFA
429	ZNF652	Literature ChIP-seq,157;ARCHS4 Coexpression,672;Enrichr Queries,707;GTEx Coexpression,534	ETS1,VEGFA
430	ZNF737	ARCHS4 Coexpression,945;GTEx Coexpression,94	NONO
431	BACH1	Literature ChIP-seq,60;ARCHS4 Coexpression,1240;ENCODE ChIP-seq,27;Enrichr Queries,321;ReMap ChIP-seq,36;GTEx Coexpression,1439	EFNB2,DDX3X,EWSR1,FUS,ETS1,VEGFA
432	ZNF143	ARCHS4 Coexpression,1431;ENCODE ChIP-seq,82;Enrichr Queries,131;ReMap ChIP-seq,178;GTEx Coexpression,782	DDX3X,EWSR1,NR2F2,ETS1,VEGFA

433	ZNF292	ARCHS4 Coexpression,686;Enrichr Queries,756;GTEx Coexpression,121	DDX3X,NONO,VEGFA
434	HOXC13	ARCHS4 Coexpression,782;Enrichr Queries,551;GTEx Coexpression,231	EFNB2,NR2F2
435	GATA6	Literature ChIP-seq,46;ARCHS4 Coexpression,717;Enrichr Queries,152;ReMap ChIP-seq,88;GTEx Coexpression,1607	EFNB2,DDX3X,NR2F2,ETS1,VEGFA
436	MAFF	ARCHS4 Coexpression,1616;ENCODE ChIP-seq,53;Enrichr Queries,716;ReMap ChIP-seq,147;GTEx Coexpression,79	FUS,EWSR1,EBF1,ETS1,VEGFA
437	ZNF583	ARCHS4 Coexpression,548;Enrichr Queries,579;GTEx Coexpression,441	ETS1,VEGFA
438	MTF2	Literature ChIP-seq,19;ARCHS4 Coexpression,1439;GTEx Coexpression,111	EFNB2,FUS,EBF1,PGK1,NR2F2,VEGFA
439	NFKB2	ARCHS4 Coexpression,790;Enrichr Queries,686;ReMap ChIP-seq,100;GTEx Coexpression,520	NR2F2,ETS1,VEGFA
440	MBD4	ARCHS4 Coexpression,1113;ENCODE ChIP-seq,72;Enrichr Queries,387;ReMap ChIP-seq,3;GTEx Coexpression,1050	DDX3X,FUS,PGK1,ETS1,VEGFA
441	SNAPC4	ARCHS4 Coexpression,356;Enrichr Queries,1337;ReMap ChIP-seq,240;GTEx Coexpression,175	EWSR1,FUS
442	NR1I2	Literature ChIP-seq,137;ARCHS4 Coexpression,899;Enrichr Queries,401;GTEx Coexpression,680	NR2F2,ETS1,VEGFA
443	TFDP1	ARCHS4 Coexpression,235;Enrichr Queries,794;ReMap ChIP-seq,257;GTEx Coexpression,835	FUS,PGK1,ETS1
444	NR2F6	ARCHS4 Coexpression,708;Enrichr Queries,658;ReMap ChIP-seq,84;GTEx Coexpression,671	NR2F2,VEGFA
445	PRDM6	ARCHS4 Coexpression,90;Enrichr Queries,267;GTEx Coexpression,1239	EFNB2,EBF1,NR2F2
446	JUND	Literature ChIP-seq,79;ARCHS4 Coexpression,1107;ENCODE ChIP-seq,5;Enrichr Queries,615;ReMap ChIP-seq,124;GTEx Coexpression,1264	FUS,EWSR1,PGK1,NR2F2,ETS1,VEGFA
447	DMRT1	Literature ChIP-seq,111;ARCHS4 Coexpression,567;Enrichr Queries,518;GTEx Coexpression,940	EBF1,NR2F2
448	NR4A2	Literature ChIP-seq,153;ARCHS4 Coexpression,608;Enrichr Queries,188;GTEx Coexpression,1188	EFNB2,NR2F2,ETS1,VEGFA
449	PRDM2	ARCHS4 Coexpression,252;Enrichr Queries,1075;GTEx Coexpression,280	ETS1
450	ETS2	Literature ChIP-seq,150;ARCHS4 Coexpression,910;Enrichr Queries,336;GTEx Coexpression,747	EFNB2,ETS1,VEGFA
451	HOXB8	ARCHS4 Coexpression,64;Enrichr Queries,151;GTEx Coexpression,1395	EFNB2,FUS,NONO,EBF1,NR2F2,ETS1
452	TCF12	ARCHS4 Coexpression,168;ENCODE ChIP-seq,16;Enrichr Queries,802;ReMap ChIP-seq,98;GTEx Coexpression,1600	NONO,NR2F2,ETS1
453	TLX1	ARCHS4 Coexpression,649;Enrichr Queries,501;GTEx Coexpression,461	EBF1,NR2F2
454	MBD2	ARCHS4 Coexpression,174;Enrichr Queries,418;ReMap ChIP-seq,273;GTEx Coexpression,1284	DDX3X,ETS1,VEGFA
455	ASCL1	ARCHS4 Coexpression,635;Enrichr Queries,225;ReMap ChIP-seq,208;GTEx Coexpression,1084	EBF1,NR2F2,VEGFA
456	TBX21	ARCHS4 Coexpression,177;Enrichr Queries,1172;ReMap ChIP-seq,119;GTEx Coexpression,690	NR2F2,ETS1
457	ARID3C	ARCHS4 Coexpression,753;GTEx Coexpression,326	
458	DNTTIP1	ARCHS4 Coexpression,763;GTEx Coexpression,317	
459	TERF2	ARCHS4 Coexpression,1038;Enrichr Queries,357;ReMap ChIP-seq,244;GTEx Coexpression,527	DDX3X,ETS1,VEGFA
460	ZNF550	ARCHS4 Coexpression,1091;Enrichr Queries,361;GTEx Coexpression,174	EFNB2,NONO,ETS1,VEGFA
461	HMGA2	ARCHS4 Coexpression,23;Enrichr Queries,159;GTEx Coexpression,1448	EFNB2,NONO,NR2F2,ETS1,VEGFA
462	RLF	ARCHS4 Coexpression,83;Enrichr Queries,852;GTEx Coexpression,697	DDX3X,VEGFA
463	HMGA1	ARCHS4 Coexpression,70;Enrichr Queries,697;GTEx Coexpression,866	FUS,NONO,PGK1,VEGFA
464	BATF	ARCHS4 Coexpression,599;ENCODE ChIP-seq,110;Enrichr Queries,1127;ReMap ChIP-seq,239;GTEx Coexpression,649	ETS1
465	SP4	ARCHS4 Coexpression,1330;ENCODE ChIP-seq,105;Enrichr Queries,396;ReMap ChIP-seq,130;GTEx Coexpression,763	EFNB2,DDX3X,EBF1,ETS1
466	NFIL3	ARCHS4 Coexpression,557;Enrichr Queries,729;GTEx Coexpression,353	ETS1,VEGFA
467	SOX1	ARCHS4 Coexpression,305;Enrichr Queries,58;GTEx Coexpression,1276	EFNB2,EBF1,NR2F2,VEGFA
468	ETV6	ARCHS4 Coexpression,523;Enrichr Queries,823;ReMap ChIP-seq,238;GTEx Coexpression,602	ETS1,VEGFA
469	TSHZ2	ARCHS4 Coexpression,1289;Enrichr Queries,325;GTEx Coexpression,26	EBF1,NR2F2,ETS1
470	PLAGL1	ARCHS4 Coexpression,604;Enrichr Queries,25;GTEx Coexpression,1013	EFNB2,EBF1,NR2F2,ETS1,VEGFA
471	HOXA4	ARCHS4 Coexpression,636;Enrichr Queries,72;GTEx Coexpression,934	EFNB2,EBF1,NR2F2,ETS1
472	ZKSCAN4	ARCHS4 Coexpression,237;Enrichr Queries,351;GTEx Coexpression,1055	EFNB2,NONO,ETS1,VEGFA
473	CREB3	ARCHS4 Coexpression,566;Enrichr Queries,585;ReMap ChIP-seq,278;GTEx Coexpression,762	ETS1,VEGFA
474	PRDM12	ARCHS4 Coexpression,765;Enrichr Queries,593;GTEx Coexpression,286	EBF1,NR2F2
475	PPARD	Literature ChIP-seq,99;ARCHS4 Coexpression,1278;Enrichr Queries,651;GTEx Coexpression,165	EFNB2,ETS1,VEGFA
476	ZNF514	ARCHS4 Coexpression,645;Enrichr Queries,905;GTEx Coexpression,101	NR2F2,VEGFA
477	BATF3	ARCHS4 Coexpression,450;Enrichr Queries,1026;GTEx Coexpression,177	ETS1
478	RAG1	ARCHS4 Coexpression,5;Enrichr Queries,791;GTEx Coexpression,862	FUS,EBF1,ETS1
479	ZBTB7A	ARCHS4 Coexpression,1387;ENCODE ChIP-seq,75;Enrichr Queries,397;ReMap ChIP-seq,103;GTEx Coexpression,804	DDX3X,NR2F2,ETS1,VEGFA
480	IRF8	Literature ChIP-seq,76;ARCHS4 Coexpression,696;Enrichr Queries,902;GTEx Coexpression,539	ETS1
481	LHX5	ARCHS4 Coexpression,856;Enrichr Queries,529;GTEx Coexpression,276	EBF1,NR2F2
482	ZBTB1	ARCHS4 Coexpression,20;Enrichr Queries,441;GTEx Coexpression,1201	DDX3X,NR2F2,ETS1,VEGFA
483	EGR2	ARCHS4 Coexpression,713;Enrichr Queries,724;GTEx Coexpression,226	ETS1,VEGFA
484	SREBF1	Literature ChIP-seq,138;ARCHS4 Coexpression,876;ENCODE ChIP-seq,37;Enrichr Queries,747;ReMap ChIP-seq,107;GTEx Coexpression,1422	FUS,EWSR1,ETS1,VEGFA
485	ZNF175	ARCHS4 Coexpression,1202;Enrichr Queries,704;ReMap ChIP-seq,90;GTEx Coexpression,222	NR2F2,VEGFA
486	HEY1	ARCHS4 Coexpression,99;Enrichr Queries,16;GTEx Coexpression,1555	EFNB2,EBF1,NR2F2,ETS1,VEGFA
487	CEBPA	Literature ChIP-seq,114;ARCHS4 Coexpression,1179;Enrichr Queries,730;ReMap ChIP-seq,106;GTEx Coexpression,655	NR2F2,ETS1,VEGFA
488	TIGD1	ARCHS4 Coexpression,995;GTEx Coexpression,119	FUS
489	ZNF24	ARCHS4 Coexpression,25;Enrichr Queries,772;ReMap ChIP-seq,70;GTEx Coexpression,1363	DDX3X,NONO,NR2F2,VEGFA
490	KLF1	Literature ChIP-seq,92;ARCHS4 Coexpression,662;Enrichr Queries,1174;ReMap ChIP-seq,121;GTEx Coexpression,740	NR2F2,VEGFA
491	EN1	ARCHS4 Coexpression,796;Enrichr Queries,211;GTEx Coexpression,669	EFNB2,EBF1,NR2F2
492	RFX1	ARCHS4 Coexpression,503;Enrichr Queries,427;ReMap ChIP-seq,228;GTEx Coexpression,1080	NR2F2,ETS1,VEGFA
493	FOSB	ARCHS4 Coexpression,1251;Enrichr Queries,389;GTEx Coexpression,44	EFNB2,DDX3X,ETS1,VEGFA
494	ATF6	ARCHS4 Coexpression,471;Enrichr Queries,851;GTEx Coexpression,363	ETS1,VEGFA
495	ZNF711	ARCHS4 Coexpression,94;Enrichr Queries,582;GTEx Coexpression,1009	EFNB2,NONO,NR2F2
496	PKNOX1	ARCHS4 Coexpression,919;Enrichr Queries,413;ReMap ChIP-seq,63;GTEx Coexpression,852	EFNB2,NR2F2,ETS1,VEGFA
497	GATA4	Literature ChIP-seq,45;ARCHS4 Coexpression,1606;Enrichr Queries,12;ReMap ChIP-seq,28;GTEx Coexpression,1120	EFNB2,EWSR1,EBF1,NR2F2,ETS1,VEGFA

498	ZNF525	ARCHS4 Coexpression,1089;GTEx Coexpression,39	EWSR1,NONO
499	FOXC2	ARCHS4 Coexpression,96;Enrichr Queries,27;GTEx Coexpression,1571	EFNB2,EBF1,NR2F2,ETS1,VEGFA
500	LMX1B	ARCHS4 Coexpression,460;Enrichr Queries,561;GTEx Coexpression,673	EBF1,NR2F2
501	ZNF544	ARCHS4 Coexpression,1242;Enrichr Queries,416;GTEx Coexpression,38	EFNB2,FUS,EWSR1,ETS1,VEGFA
502	ELF3	ARCHS4 Coexpression,648;Enrichr Queries,1018;ReMap ChIP-seq,96;GTEx Coexpression,500	NR2F2,VEGFA
503	ZEB2	ARCHS4 Coexpression,582;Enrichr Queries,103;ReMap ChIP-seq,76;GTEx Coexpression,1506	EBF1,NR2F2,ETS1,VEGFA
504	FEZF1	ARCHS4 Coexpression,49;Enrichr Queries,560;GTEx Coexpression,1092	NONO,EBF1,PGK1,NR2F2
505	PRRX1	ARCHS4 Coexpression,1391;Enrichr Queries,262;GTEx Coexpression,56	EBF1,NR2F2,ETS1
506	ZNF597	ARCHS4 Coexpression,1236;Enrichr Queries,224;GTEx Coexpression,255	EFNB2,DDX3X,ETS1
507	ZNF275	ARCHS4 Coexpression,142;Enrichr Queries,101;GTEx Coexpression,1472	EFNB2,DDX3X,FUS,ETS1,VEGFA
508	VEZF1	ARCHS4 Coexpression,928;Enrichr Queries,202;GTEx Coexpression,587	DDX3X,NR2F2,ETS1,VEGFA
509	POU4F1	ARCHS4 Coexpression,36;Enrichr Queries,554;GTEx Coexpression,1127	EBF1,NR2F2
510	FOXL1	ARCHS4 Coexpression,106;Enrichr Queries,190;GTEx Coexpression,1423	EFNB2,EBF1,NR2F2,ETS1
511	SOX11	Literature ChIP-seq,93;ARCHS4 Coexpression,983;Enrichr Queries,128;ReMap ChIP-seq,87;GTEx Coexpression,1576	EFNB2,EBF1,NR2F2,VEGFA
512	NR4A1	ARCHS4 Coexpression,1119;Enrichr Queries,995;ReMap ChIP-seq,125;GTEx Coexpression,57	NR2F2,VEGFA
513	STAT2	ARCHS4 Coexpression,918;ENCODE ChIP-seq,36;Enrichr Queries,530;ReMap ChIP-seq,65;GTEx Coexpression,1324	DDX3X,ETS1,VEGFA
514	PKNOX2	ARCHS4 Coexpression,316;Enrichr Queries,79;GTEx Coexpression,1329	EFNB2,EBF1,NR2F2,ETS1
515	ATF1	ARCHS4 Coexpression,1306;ENCODE ChIP-seq,95;Enrichr Queries,468;ReMap ChIP-seq,237;GTEx Coexpression,778	DDX3X,ETS1,VEGFA
516	ZNF302	ARCHS4 Coexpression,733;Enrichr Queries,606;GTEx Coexpression,393	ETS1,VEGFA
517	ZBED2	ARCHS4 Coexpression,216;Enrichr Queries,1336;GTEx Coexpression,181	ETS1,VEGFA
518	ETV4	ARCHS4 Coexpression,205;Enrichr Queries,691;ReMap ChIP-seq,115;GTEx Coexpression,1301	NONO,ETS1,VEGFA
519	POU2F1	ARCHS4 Coexpression,874;Enrichr Queries,818;ReMap ChIP-seq,211;GTEx Coexpression,414	EBF1,ETS1
520	ZNF2	ARCHS4 Coexpression,375;Enrichr Queries,789	ETS1,VEGFA
521	RELB	ARCHS4 Coexpression,586;Enrichr Queries,717;ReMap ChIP-seq,93;GTEx Coexpression,932	NR2F2,ETS1,VEGFA
522	CENPX	GTEx Coexpression,582	
523	DMRTA2	ARCHS4 Coexpression,597;Enrichr Queries,251;GTEx Coexpression,902	EFNB2,EBF1,NR2F2
524	ZNF436	ARCHS4 Coexpression,348;Enrichr Queries,109;GTEx Coexpression,1295	EFNB2,NR2F2,ETS1,VEGFA
525	ZBTB34	ARCHS4 Coexpression,791;Enrichr Queries,437;GTEx Coexpression,525	EFNB2,ETS1,VEGFA
526	BNC1	ARCHS4 Coexpression,869;Enrichr Queries,287;GTEx Coexpression,598	EFNB2,NR2F2,ETS1
527	FOXP4	ARCHS4 Coexpression,192;Enrichr Queries,171;GTEx Coexpression,1394	EFNB2,FUS,NR2F2,ETS1,VEGFA
528	RREB1	ARCHS4 Coexpression,568;Enrichr Queries,421;GTEx Coexpression,769	NR2F2,ETS1,VEGFA
529	RFX3	ARCHS4 Coexpression,695;Enrichr Queries,796;GTEx Coexpression,268	NR2F2,ETS1
530	ZNF777	ARCHS4 Coexpression,201;Enrichr Queries,1132;GTEx Coexpression,430	FUS,NR2F2
531	ZFY	ARCHS4 Coexpression,312;Enrichr Queries,209;GTEx Coexpression,1244	EFNB2,NR2F2,ETS1,VEGFA
532	CTCFL	ARCHS4 Coexpression,998;ENCODE ChIP-seq,90;Enrichr Queries,576;ReMap ChIP-seq,110;GTEx Coexpression,1173	ETS1,VEGFA
533	PRDM9	ARCHS4 Coexpression,171;Enrichr Queries,447;GTEx Coexpression,1153	NONO,NR2F2,ETS1,VEGFA
534	ZNF350	ARCHS4 Coexpression,864;Enrichr Queries,249;GTEx Coexpression,658	EFNB2,NR2F2,VEGFA
535	HOXD9	ARCHS4 Coexpression,32;Enrichr Queries,237;GTEx Coexpression,1503	FUS,EBF1,NR2F2,ETS1
536	ZNF318	ARCHS4 Coexpression,211;Enrichr Queries,1155;ReMap ChIP-seq,48;GTEx Coexpression,949	DDX3X,FUS,ETS1
537	ASCL5	ARCHS4 Coexpression,880;GTEx Coexpression,302	
538	CEBPZ	ARCHS4 Coexpression,587;ENCODE ChIP-seq,109;Enrichr Queries,1077;ReMap ChIP-seq,263;GTEx Coexpression,926	DDX3X
539	ARID5B	ARCHS4 Coexpression,1051;Enrichr Queries,310;GTEx Coexpression,421	NR2F2,ETS1,VEGFA
540	ZNF319	ARCHS4 Coexpression,1061;Enrichr Queries,465;GTEx Coexpression,257	EFNB2,ETS1,VEGFA
541	TBX18	ARCHS4 Coexpression,1458;Enrichr Queries,260;GTEx Coexpression,67	EFNB2,EBF1,NR2F2
542	HMGN3	ARCHS4 Coexpression,868;ENCODE ChIP-seq,96;ReMap ChIP-seq,73;GTEx Coexpression,1345	NR2F2
543	MBD6	ARCHS4 Coexpression,1129;GTEx Coexpression,62	EWSR1
544	ZNF750	ARCHS4 Coexpression,627;Enrichr Queries,1308;ReMap ChIP-seq,155;GTEx Coexpression,297	
545	BARHL2	ARCHS4 Coexpression,1028;Enrichr Queries,484;GTEx Coexpression,279	EBF1,NR2F2
546	GTF2IRD1	ARCHS4 Coexpression,253;GTEx Coexpression,941	ETS1
547	ESRRA	ARCHS4 Coexpression,1117;ENCODE ChIP-seq,106;Enrichr Queries,858;ReMap ChIP-seq,94;GTEx Coexpression,811	ETS1,VEGFA
548	ZNF267	ARCHS4 Coexpression,1243;Enrichr Queries,308;GTEx Coexpression,242	DDX3X,ETS1,VEGFA
549	ZXDB	ARCHS4 Coexpression,97;Enrichr Queries,743;GTEx Coexpression,955	DDX3X,ETS1,VEGFA
550	DLX2	ARCHS4 Coexpression,1027;Enrichr Queries,168;GTEx Coexpression,601	EFNB2,EBF1,NR2F2,VEGFA
551	ASH1L	ARCHS4 Coexpression,377;Enrichr Queries,1119;GTEx Coexpression,303	DDX3X
552	ZBTB32	ARCHS4 Coexpression,232;Enrichr Queries,1173;GTEx Coexpression,396	ETS1
553	ZNF300	ARCHS4 Coexpression,1154;Enrichr Queries,627;GTEx Coexpression,20	EFNB2,FUS,EBF1,NR2F2
554	CREM	Literature ChIP-seq,63;ARCHS4 Coexpression,894;Enrichr Queries,588;ReMap ChIP-seq,92;GTEx Coexpression,1367	EFNB2,DDX3X,FUS,EWSR1,NR2F2,ETS1,VEGFA
555	POU3F3	ARCHS4 Coexpression,176;Enrichr Queries,214;GTEx Coexpression,1413	EFNB2,EBF1,NR2F2
556	SOX18	ARCHS4 Coexpression,1613;Enrichr Queries,189;GTEx Coexpression,2	EFNB2,EBF1,NR2F2,ETS1
557	HEY2	ARCHS4 Coexpression,267;Enrichr Queries,36;GTEx Coexpression,1501	EFNB2,EBF1,NR2F2,ETS1,VEGFA
558	CHCHD3	ARCHS4 Coexpression,738;GTEx Coexpression,470	
559	HAND2	ARCHS4 Coexpression,495;Enrichr Queries,93;GTEx Coexpression,1226	EFNB2,EBF1,NR2F2,ETS1
560	ZNF598	ARCHS4 Coexpression,307;Enrichr Queries,1238;GTEx Coexpression,274	FUS
561	ZNF534	ARCHS4 Coexpression,480;GTEx Coexpression,733	
562	ZNF256	ARCHS4 Coexpression,87;Enrichr Queries,1292;GTEx Coexpression,442	NONO
563	ZGPAT	ARCHS4 Coexpression,406;Enrichr Queries,1188;GTEx Coexpression,229	
564	AKNA	ARCHS4 Coexpression,329;Enrichr Queries,846;GTEx Coexpression,648	ETS1,VEGFA
565	NANOG	Literature ChIP-seq,50;ARCHS4 Coexpression,1269;ENCODE ChIP-seq,118;Enrichr Queries,748;ReMap ChIP-seq,1;GTEx Coexpression,1461	EFNB2,FUS,NONO,EBF1,NR2F2,ETS1,VEGFA

566	PHOX2A	ARCHS4 Coexpression,223;Enrichr Queries,217;GTEx Coexpression,1384	NONO,EBF1,NR2F2,ETS1
567	TSHZ1	ARCHS4 Coexpression,1564;Enrichr Queries,33;GTEx Coexpression,230	EFNB2,EBF1,NR2F2,ETS1,VEGFA
568	NCOA1	ARCHS4 Coexpression,444;Enrichr Queries,820;ReMap ChIP-seq,74;GTEx Coexpression,1102	NR2F2,ETS1,VEGFA
569	FOXQ1	ARCHS4 Coexpression,502;Enrichr Queries,191;GTEx Coexpression,1138	EFNB2,NR2F2,ETS1,VEGFA
570	ETV5	ARCHS4 Coexpression,571;Enrichr Queries,354;GTEx Coexpression,907	EFNB2,ETS1,VEGFA
571	ZNF286B	ARCHS4 Coexpression,970;GTEx Coexpression,253	
572	INSM2	ARCHS4 Coexpression,579;Enrichr Queries,781;GTEx Coexpression,479	EBF1,NR2F2
573	SOX5	ARCHS4 Coexpression,196;Enrichr Queries,40;GTEx Coexpression,1603	EFNB2,EBF1,NR2F2,ETS1,VEGFA
574	ZNF223	ARCHS4 Coexpression,103;Enrichr Queries,1350;GTEx Coexpression,390	FUS
575	MBD3	ARCHS4 Coexpression,117;Enrichr Queries,979;ReMap ChIP-seq,52;GTEx Coexpression,1310	FUS,NR2F2
576	FOS	ARCHS4 Coexpression,931;ENCODE ChIP-seq,52;Enrichr Queries,735;ReMap ChIP-seq,191;GTEx Coexpression,1164	EWSR1,PGK1,ETS1,VEGFA
577	YBX1	ARCHS4 Coexpression,69;Enrichr Queries,439;GTEx Coexpression,1336	DDX3X,FUS,NONO,PGK1
578	NFIC	ARCHS4 Coexpression,1373;ENCODE ChIP-seq,104;Enrichr Queries,282;ReMap ChIP-seq,245;GTEx Coexpression,1073	NR2F2,ETS1,VEGFA
579	ZNF202	ARCHS4 Coexpression,231;Enrichr Queries,570;GTEx Coexpression,1046	FUS,ETS1,VEGFA
580	DMRT3	ARCHS4 Coexpression,188;Enrichr Queries,485;GTEx Coexpression,1174	EFNB2,EBF1,NR2F2
581	NEUROG1	ARCHS4 Coexpression,200;Enrichr Queries,507;GTEx Coexpression,1143	EBF1,NR2F2
582	ELF5	Literature ChIP-seq,131;ARCHS4 Coexpression,1471;Enrichr Queries,304;ReMap ChIP-seq,290;GTEx Coexpression,888	EFNB2,EBF1,ETS1
583	HHEX	ARCHS4 Coexpression,1070;Enrichr Queries,1011;ReMap ChIP-seq,234;GTEx Coexpression,154	ETS1,VEGFA
584	ZBTB24	ARCHS4 Coexpression,85;Enrichr Queries,636;GTEx Coexpression,1133	ETS1,VEGFA
585	DBX1	ARCHS4 Coexpression,340;Enrichr Queries,521;GTEx Coexpression,994	NONO,EBF1,NR2F2
586	UNCX	ARCHS4 Coexpression,1046;Enrichr Queries,513;GTEx Coexpression,296	EBF1,NR2F2
587	THYN1	ARCHS4 Coexpression,822;GTEx Coexpression,415	
588	SIX2	ARCHS4 Coexpression,1071;Enrichr Queries,320;ReMap ChIP-seq,7;GTEx Coexpression,1078	EFNB2,EBF1,NR2F2,ETS1
589	ZNF311	ARCHS4 Coexpression,1604;Enrichr Queries,204;GTEx Coexpression,53	EBF1,NR2F2,ETS1
590	ZBED5	ARCHS4 Coexpression,484;GTEx Coexpression,757	
591	KLF12	ARCHS4 Coexpression,187;Enrichr Queries,179;GTEx Coexpression,1496	EFNB2,EBF1,NR2F2,ETS1
592	TFAP4	ARCHS4 Coexpression,1080;Enrichr Queries,815;ReMap ChIP-seq,129;GTEx Coexpression,469	ETS1,VEGFA
593	NFATC3	ARCHS4 Coexpression,118;Enrichr Queries,770;GTEx Coexpression,984	ETS1,VEGFA
594	NANOGP8	ARCHS4 Coexpression,650;GTEx Coexpression,599	
595	TFEB	Literature ChIP-seq,135;ARCHS4 Coexpression,866;Enrichr Queries,753;GTEx Coexpression,744	ETS1,VEGFA
596	EMX2	ARCHS4 Coexpression,400;Enrichr Queries,378;GTEx Coexpression,1099	EFNB2,EBF1,NR2F2
597	ZNF90	ARCHS4 Coexpression,370;Enrichr Queries,1375;GTEx Coexpression,136	NONO
598	ZNF629	ARCHS4 Coexpression,298;Enrichr Queries,316;GTEx Coexpression,1267	EFNB2,FUS,NR2F2,VEGFA
599	PLAGL2	ARCHS4 Coexpression,271;Enrichr Queries,1133;GTEx Coexpression,477	FUS,VEGFA
600	DMRTB1	ARCHS4 Coexpression,907;Enrichr Queries,80;GTEx Coexpression,898	EFNB2,EBF1,NR2F2,ETS1
601	ATF7	ARCHS4 Coexpression,1285;Enrichr Queries,1106;ReMap ChIP-seq,81;GTEx Coexpression,42	EBF1,NR2F2,ETS1,VEGFA
602	PRDM14	Literature ChIP-seq,34;ARCHS4 Coexpression,1231;Enrichr Queries,522;ReMap ChIP-seq,99;GTEx Coexpression,1257	NONO,EBF1,NR2F2,VEGFA
603	ZBTB7C	ARCHS4 Coexpression,754;Enrichr Queries,626;GTEx Coexpression,508	EFNB2,EBF1
604	SATB2	ARCHS4 Coexpression,1109;Enrichr Queries,84;GTEx Coexpression,695	EFNB2,EBF1,NR2F2,ETS1
605	JDP2	ARCHS4 Coexpression,792;Enrichr Queries,673;GTEx Coexpression,425	ETS1,VEGFA
606	NEUROD2	Literature ChIP-seq,70;ARCHS4 Coexpression,921;Enrichr Queries,970;GTEx Coexpression,561	EFNB2,NR2F2,VEGFA
607	ZNF462	ARCHS4 Coexpression,999;Enrichr Queries,384;GTEx Coexpression,511	EFNB2,NR2F2,ETS1
608	HIC2	ARCHS4 Coexpression,841;Enrichr Queries,182;GTEx Coexpression,872	EFNB2,DDX3X,ETS1,VEGFA
609	TLX2	ARCHS4 Coexpression,337;Enrichr Queries,493;GTEx Coexpression,1071	NONO,EBF1,NR2F2
610	NKX61	ARCHS4 Coexpression,895;GTEx Coexpression,373	
611	MXI1	ARCHS4 Coexpression,1300;ENCODE ChIP-seq,74;Enrichr Queries,303;ReMap ChIP-seq,145;GTEx Coexpression,1350	EWSR1,PGK1,ETS1,VEGFA
612	FOXC1	ARCHS4 Coexpression,287;Enrichr Queries,43;GTEx Coexpression,1574	EFNB2,EBF1,NR2F2,ETS1,VEGFA
613	GFI1B	Literature ChIP-seq,71;ARCHS4 Coexpression,1426;Enrichr Queries,1125;ReMap ChIP-seq,127;GTEx Coexpression,428	FUS,PGK1,ETS1,VEGFA
614	SP140	ARCHS4 Coexpression,183;Enrichr Queries,1182;GTEx Coexpression,542	ETS1
615	ZNF232	ARCHS4 Coexpression,749;Enrichr Queries,245;GTEx Coexpression,914	NR2F2,ETS1,VEGFA
616	IRF2	ARCHS4 Coexpression,846;Enrichr Queries,769;ReMap ChIP-seq,35;GTEx Coexpression,894	DDX3X,ETS1,VEGFA
617	MAX	ARCHS4 Coexpression,1516;ENCODE ChIP-seq,10;Enrichr Queries,764;ReMap ChIP-seq,177;GTEx Coexpression,716	EWSR1,NONO,PGK1,ETS1,VEGFA
618	ZNF589	ARCHS4 Coexpression,239;Enrichr Queries,1341;ReMap ChIP-seq,284;GTEx Coexpression,683	NONO
619	ZBTB6	ARCHS4 Coexpression,44;Enrichr Queries,633;GTEx Coexpression,1234	DDX3X,ETS1
620	ADNP2	ARCHS4 Coexpression,262;Enrichr Queries,430;GTEx Coexpression,1220	DDX3X,NONO,ETS1,VEGFA
621	PITX2	ARCHS4 Coexpression,850;Enrichr Queries,172;GTEx Coexpression,890	EFNB2,EBF1,NR2F2,ETS1
622	SHOX	ARCHS4 Coexpression,1186;Enrichr Queries,602;GTEx Coexpression,125	EBF1,NR2F2
623	ZHX1	ARCHS4 Coexpression,438;Enrichr Queries,773;ReMap ChIP-seq,186;GTEx Coexpression,1155	NR2F2,ETS1
624	ZNF596	ARCHS4 Coexpression,651;Enrichr Queries,1076;GTEx Coexpression,187	FUS,VEGFA
625	FOXF1	ARCHS4 Coexpression,1362;Enrichr Queries,29;GTEx Coexpression,524	EFNB2,EBF1,NR2F2,ETS1,VEGFA
626	KMT2A	ARCHS4 Coexpression,823;ReMap ChIP-seq,21;GTEx Coexpression,1072	EFNB2,EBF1
627	PAX1	ARCHS4 Coexpression,1126;Enrichr Queries,482;GTEx Coexpression,310	EBF1,NR2F2
628	FOSL1	ARCHS4 Coexpression,1111;ENCODE ChIP-seq,59;Enrichr Queries,696;ReMap ChIP-seq,183;GTEx Coexpression,1148	DDX3X,ETS1,VEGFA
629	ZFHX3	ARCHS4 Coexpression,767;Enrichr Queries,166;GTEx Coexpression,986	EFNB2,EBF1,NR2F2,ETS1
630	HNF1A	ARCHS4 Coexpression,825;Enrichr Queries,652;GTEx Coexpression,444	ETS1,VEGFA
631	HES7	ARCHS4 Coexpression,437;Enrichr Queries,660;GTEx Coexpression,829	EBF1,VEGFA
632	PRDM4	ARCHS4 Coexpression,1418;Enrichr Queries,181;GTEx Coexpression,334	EFNB2,DDX3X,ETS1,VEGFA
633	ZNF157	ARCHS4 Coexpression,402;Enrichr Queries,1215;GTEx Coexpression,316	

634	ZNF45	ARCHS4 Coexpression,34;Enrichr Queries,1317;GTEx Coexpression,585	DDX3X,NONO
635	BARX2	ARCHS4 Coexpression,617;Enrichr Queries,388;GTEx Coexpression,931	EFNB2,NR2F2,ETS1
636	CHAMP1	ARCHS4 Coexpression,68;GTEx Coexpression,1223	FUS,NONO
637	ZNF410	ARCHS4 Coexpression,110;Enrichr Queries,370;GTEx Coexpression,1457	FUS,PGK1,NR2F2,VEGFA
638	TBX1	ARCHS4 Coexpression,886;Enrichr Queries,83;GTEx Coexpression,969	EFNB2,NR2F2,ETS1,VEGFA
639	PBX4	ARCHS4 Coexpression,884;Enrichr Queries,855;GTEx Coexpression,200	ETS1,VEGFA
640	BORCS8MEF2B	ARCHS4 Coexpression,647	
641	OTP	ARCHS4 Coexpression,251;Enrichr Queries,508;GTEx Coexpression,1182	EBF1,NR2F2
642	ZKSCAN2	ARCHS4 Coexpression,40;Enrichr Queries,467;GTEx Coexpression,1434	EFNB2,NONO,ETS1,VEGFA
643	RARB	Literature ChIP-seq,68;ARCHS4 Coexpression,1068;Enrichr Queries,2;GTEx Coexpression,1452	EFNB2,EBF1,NR2F2,ETS1,VEGFA
644	E2F6	ARCHS4 Coexpression,1199;ENCODE ChIP-seq,92;Enrichr Queries,343;ReMap ChIP-seq,148;GTEx Coexpression,1459	NR2F2,ETS1,VEGFA
645	HOXC10	ARCHS4 Coexpression,853;Enrichr Queries,611;GTEx Coexpression,481	EBF1,NR2F2
646	PATZ1	ARCHS4 Coexpression,51;Enrichr Queries,338;GTEx Coexpression,1556	FUS,NONO,NR2F2,ETS1,VEGFA
647	NR6A1	ARCHS4 Coexpression,926;Enrichr Queries,15;GTEx Coexpression,1006	EFNB2,EBF1,NR2F2,ETS1,VEGFA
648	NR1H4	ARCHS4 Coexpression,735;Enrichr Queries,624;GTEx Coexpression,588	NR2F2,ETS1
649	ZBTB3	ARCHS4 Coexpression,661;Enrichr Queries,955;GTEx Coexpression,331	VEGFA
650	HELT	ARCHS4 Coexpression,631;Enrichr Queries,489;GTEx Coexpression,830	EBF1,NR2F2
651	ZNF804B	ARCHS4 Coexpression,459;Enrichr Queries,1325;GTEx Coexpression,168	VEGFA
652	FOXD4	ARCHS4 Coexpression,761;Enrichr Queries,882;GTEx Coexpression,311	NR2F2
653	ZNF805	ARCHS4 Coexpression,653;Enrichr Queries,664;GTEx Coexpression,637	ETS1,VEGFA
654	SAFB	ARCHS4 Coexpression,4;GTEx Coexpression,1299	FUS,EWSR1,NONO
655	ZNF92	ARCHS4 Coexpression,1090;Enrichr Queries,1288;ReMap ChIP-seq,55;GTEx Coexpression,176	EWSR1,PGK1
656	TLX3	ARCHS4 Coexpression,533;Enrichr Queries,503;GTEx Coexpression,922	EBF1,NR2F2
657	FOXJ3	ARCHS4 Coexpression,310;Enrichr Queries,1059;GTEx Coexpression,589	ETS1
658	NPAS2	ARCHS4 Coexpression,904;Enrichr Queries,247;GTEx Coexpression,807	EFNB2,NR2F2,VEGFA
659	BARX1	ARCHS4 Coexpression,121;Enrichr Queries,475;GTEx Coexpression,1364	FUS,EBF1,NR2F2
660	TFCP2	ARCHS4 Coexpression,9;Enrichr Queries,825;GTEx Coexpression,1128	DDX3X,FUS,NONO,ETS1,VEGFA
661	SOX14	ARCHS4 Coexpression,490;Enrichr Queries,206;GTEx Coexpression,1266	EBF1,NR2F2,ETS1
662	NR4A3	ARCHS4 Coexpression,519;Enrichr Queries,680;GTEx Coexpression,766	ETS1,VEGFA
663	CSRNP1	ARCHS4 Coexpression,378;GTEx Coexpression,933	
664	ZNF415	ARCHS4 Coexpression,865;Enrichr Queries,828;GTEx Coexpression,275	EFNB2,VEGFA
665	BHLHE22	ARCHS4 Coexpression,1284;ReMap ChIP-seq,279;GTEx Coexpression,406	
666	ASCL3	ARCHS4 Coexpression,371;Enrichr Queries,1285;GTEx Coexpression,315	
667	ZNF513	ARCHS4 Coexpression,546;Enrichr Queries,1343;GTEx Coexpression,82	EWSR1
668	ZNF732	ARCHS4 Coexpression,854;GTEx Coexpression,460	
669	ZNF212	ARCHS4 Coexpression,332;Enrichr Queries,1171;GTEx Coexpression,471	EWSR1,VEGFA
670	OTX2	ARCHS4 Coexpression,1280;Enrichr Queries,212;ReMap ChIP-seq,262;GTEx Coexpression,882	EFNB2,EBF1,NR2F2
671	JUNB	ARCHS4 Coexpression,1075;Enrichr Queries,700;ReMap ChIP-seq,78;GTEx Coexpression,784	ETS1,VEGFA
672	HOXA7	ARCHS4 Coexpression,1039;Enrichr Queries,117;GTEx Coexpression,824	EFNB2,EBF1,NR2F2,ETS1
673	ZNF33B	ARCHS4 Coexpression,948;Enrichr Queries,616;GTEx Coexpression,418	NR2F2,VEGFA
674	TRAFD1	ARCHS4 Coexpression,365;Enrichr Queries,1104;GTEx Coexpression,514	VEGFA
675	ZNF160	ARCHS4 Coexpression,457;Enrichr Queries,281;GTEx Coexpression,1246	PGK1,ETS1,VEGFA
676	MYCL	ARCHS4 Coexpression,742;GTEx Coexpression,581	
677	ZNF641	ARCHS4 Coexpression,454;Enrichr Queries,542;GTEx Coexpression,989	ETS1,VEGFA
678	ZNF850	ARCHS4 Coexpression,979;GTEx Coexpression,345	
679	FOXE1	ARCHS4 Coexpression,888;Enrichr Queries,936;GTEx Coexpression,163	NR2F2,VEGFA
680	SMAD9	ARCHS4 Coexpression,1125;Enrichr Queries,243;GTEx Coexpression,621	EBF1,NR2F2,VEGFA
681	USF2	ARCHS4 Coexpression,1108;ENCODE ChIP-seq,48;Enrichr Queries,374;ReMap ChIP-seq,195;GTEx Coexpression,1591	DDX3X,NR2F2,ETS1,VEGFA
682	FOXK2	ARCHS4 Coexpression,338;Enrichr Queries,1153;ReMap ChIP-seq,189;GTEx Coexpression,973	FUS,VEGFA
683	ZNF251	ARCHS4 Coexpression,357;Enrichr Queries,360;GTEx Coexpression,1273	EFNB2,NR2F2,VEGFA
684	ZNF664	ARCHS4 Coexpression,28;Enrichr Queries,470;GTEx Coexpression,1493	FUS,NONO,ETS1,VEGFA
685	NEUROD4	ARCHS4 Coexpression,113;Enrichr Queries,706;GTEx Coexpression,1172	EBF1,NR2F2
686	FOXD4L3	ARCHS4 Coexpression,247;Enrichr Queries,874;GTEx Coexpression,871	EBF1,NR2F2
687	TBX3	Literature ChIP-seq,3;ARCHS4 Coexpression,1073;Enrichr Queries,41;GTEx Coexpression,1540	EFNB2,EBF1,PGK1,NR2F2,ETS1,VEGFA
688	TBX15	ARCHS4 Coexpression,522;Enrichr Queries,293;GTEx Coexpression,1178	EBF1,NR2F2,ETS1
689	ZNF691	ARCHS4 Coexpression,751;Enrichr Queries,795;GTEx Coexpression,452	ETS1,VEGFA
690	ZFP41	ARCHS4 Coexpression,139;Enrichr Queries,1168;GTEx Coexpression,692	FUS,VEGFA
691	ZNF654	ARCHS4 Coexpression,79;Enrichr Queries,958;GTEx Coexpression,963	DDX3X,VEGFA
692	ATOH1	ARCHS4 Coexpression,540;Enrichr Queries,232;GTEx Coexpression,1235	EFNB2,EBF1,NR2F2
693	IRX4	ARCHS4 Coexpression,675;Enrichr Queries,964;GTEx Coexpression,371	NR2F2
694	TRERF1	ARCHS4 Coexpression,241;Enrichr Queries,454;GTEx Coexpression,1316	NR2F2,ETS1,VEGFA
695	CSRNP3	ARCHS4 Coexpression,829;GTEx Coexpression,515	
696	SIX4	ARCHS4 Coexpression,639;Enrichr Queries,137;GTEx Coexpression,1242	EFNB2,NR2F2,ETS1,VEGFA
697	DPF3	ARCHS4 Coexpression,593;Enrichr Queries,135;GTEx Coexpression,1292	EFNB2,EBF1,NR2F2,ETS1
698	AHRR	ARCHS4 Coexpression,777;GTEx Coexpression,571	
699	PRDM16	Literature ChIP-seq,156;ARCHS4 Coexpression,858;Enrichr Queries,266;GTEx Coexpression,1416	EBF1,NR2F2,ETS1
700	ZNF16	ARCHS4 Coexpression,902;Enrichr Queries,603;GTEx Coexpression,521	ETS1,VEGFA
701	ATOH8	ARCHS4 Coexpression,1506;Enrichr Queries,309;GTEx Coexpression,212	EBF1,NR2F2,ETS1
702	MXD4	ARCHS4 Coexpression,1425;Enrichr Queries,381;GTEx Coexpression,224	EFNB2,ETS1,VEGFA
703	EEA1	ARCHS4 Coexpression,1009;Enrichr Queries,448;GTEx Coexpression,574	DDX3X,ETS1,VEGFA
704	RXRG	ARCHS4 Coexpression,764;Enrichr Queries,638;GTEx Coexpression,630	EBF1,VEGFA
705	ZNF565	ARCHS4 Coexpression,996;Enrichr Queries,639;GTEx Coexpression,397	NR2F2,ETS1
706	LMX1A	ARCHS4 Coexpression,320;Enrichr Queries,600;GTEx Coexpression,1112	EBF1,NR2F2

707	YBX3	ARCHS4 Coexpression,404;GTEx Coexpression,951	
708	PITX3	ARCHS4 Coexpression,418;Enrichr Queries,230;GTEx Coexpression,1390	EBF1,NR2F2,ETS1
709	THAP2	ARCHS4 Coexpression,105;Enrichr Queries,993;GTEx Coexpression,942	FUS,VEGFA
710	ZNF467	ARCHS4 Coexpression,605;Enrichr Queries,594;GTEx Coexpression,843	ETS1,VEGFA
711	ETV2	Literature ChIP-seq,90;ARCHS4 Coexpression,798;Enrichr Queries,586;GTEx Coexpression,1249	FUS,EBF1,ETS1
712	STAT4	Literature ChIP-seq,128;ARCHS4 Coexpression,851;Enrichr Queries,1022;GTEx Coexpression,724	ETS1
713	LIN28A	ARCHS4 Coexpression,218;GTEx Coexpression,1145	NONO
714	ZNF581	ARCHS4 Coexpression,395;Enrichr Queries,1402;GTEx Coexpression,248	
715	ZNF80	ARCHS4 Coexpression,837;Enrichr Queries,918;GTEx Coexpression,293	ETS1
716	MBD1	ARCHS4 Coexpression,1083;Enrichr Queries,726;GTEx Coexpression,239	ETS1,VEGFA
717	MIXL1	ARCHS4 Coexpression,882;Enrichr Queries,553;GTEx Coexpression,613	EBF1,ETS1
718	SMAD5	ARCHS4 Coexpression,1489;Enrichr Queries,129;ReMap ChIP-seq,5;GTEx Coexpression,1110	DDX3X,FUS,NR2F2,ETS1,VEGFA
719	ZNF432	ARCHS4 Coexpression,1332;Enrichr Queries,347;GTEx Coexpression,372	EFNB2,ETS1,VEGFA
720	NFYB	ARCHS4 Coexpression,1192;ENCODE ChIP-seq,84;Enrichr Queries,419;ReMap ChIP-seq,173;GTEx Coexpression,1552	PGK1,NR2F2,ETS1,VEGFA
721	HIVEP1	ARCHS4 Coexpression,1131;Enrichr Queries,688;GTEx Coexpression,234	ETS1,VEGFA
722	ZNF568	ARCHS4 Coexpression,441;Enrichr Queries,1269;GTEx Coexpression,343	
723	FOXK1	ARCHS4 Coexpression,932;Enrichr Queries,836;ReMap ChIP-seq,253;GTEx Coexpression,720	NR2F2,ETS1
724	ZNF26	ARCHS4 Coexpression,1030;Enrichr Queries,604;GTEx Coexpression,423	ETS1,VEGFA
725	SMYD3	ARCHS4 Coexpression,1037;GTEx Coexpression,335	
726	ZFP28	ARCHS4 Coexpression,857;Enrichr Queries,400;GTEx Coexpression,803	EFNB2,EBF1,VEGFA
727	MAFK	ARCHS4 Coexpression,1189;ENCODE ChIP-seq,1;Enrichr Queries,749;ReMap ChIP-seq,192;GTEx Coexpression,1303	DDX3X,FUS,EWSR1,ETS1,VEGFA
728	ZNF174	ARCHS4 Coexpression,504;Enrichr Queries,714;GTEx Coexpression,844	FUS,VEGFA
729	SOX6	ARCHS4 Coexpression,1177;Enrichr Queries,50;ReMap ChIP-seq,51;GTEx Coexpression,1474	EFNB2,EBF1,NR2F2,ETS1,VEGFA
730	NAIF1	ARCHS4 Coexpression,912;GTEx Coexpression,466	
731	MLXIP	ARCHS4 Coexpression,1006;Enrichr Queries,806;GTEx Coexpression,258	ETS1,VEGFA
732	ZNF417	ARCHS4 Coexpression,455;Enrichr Queries,1310;GTEx Coexpression,308	
733	CRX	Literature ChIP-seq,140;ARCHS4 Coexpression,1449;Enrichr Queries,552;GTEx Coexpression,625	EBF1,ETS1
734	LEUTX	ARCHS4 Coexpression,1295;Enrichr Queries,654;GTEx Coexpression,126	PGK1,NR2F2,ETS1
735	IRF6	ARCHS4 Coexpression,727;Enrichr Queries,1007;GTEx Coexpression,344	EFNB2
736	ZNF460	ARCHS4 Coexpression,1572;Enrichr Queries,391;GTEx Coexpression,116	EFNB2,DDX3X,PGK1,VEGFA
737	IRF5	ARCHS4 Coexpression,732;Enrichr Queries,1146;GTEx Coexpression,207	PGK1,ETS1
738	ZNF592	ARCHS4 Coexpression,481;Enrichr Queries,1092;ReMap ChIP-seq,42;GTEx Coexpression,1165	FUS,ETS1
739	ZNF558	ARCHS4 Coexpression,1273;Enrichr Queries,342;GTEx Coexpression,472	EBF1,ETS1,VEGFA
740	NKX28	ARCHS4 Coexpression,784;GTEx Coexpression,608	
741	MZF1	ARCHS4 Coexpression,166;Enrichr Queries,330;GTEx Coexpression,1593	FUS,NR2F2,ETS1,VEGFA
742	REPIN1	ARCHS4 Coexpression,149;Enrichr Queries,968;GTEx Coexpression,975	FUS,VEGFA
743	FOXO6	ARCHS4 Coexpression,862;Enrichr Queries,185;GTEx Coexpression,1045	EFNB2,NR2F2,ETS1,VEGFA
744	ZNF165	ARCHS4 Coexpression,381;Enrichr Queries,740;ReMap ChIP-seq,291;GTEx Coexpression,1378	EFNB2,VEGFA
745	ZNF782	ARCHS4 Coexpression,1021;Enrichr Queries,945;GTEx Coexpression,128	EFNB2,NONO
746	ONECUT3	ARCHS4 Coexpression,1343;Enrichr Queries,95;GTEx Coexpression,661	EFNB2,EBF1,NR2F2,ETS1
747	IRF9	ARCHS4 Coexpression,492;Enrichr Queries,877;ReMap ChIP-seq,270;GTEx Coexpression,1161	VEGFA
748	CXXC4	ARCHS4 Coexpression,848;ReMap ChIP-seq,77;GTEx Coexpression,1175	NR2F2
749	SPEN	ARCHS4 Coexpression,129;GTEx Coexpression,1271	FUS
750	ZSCAN4	ARCHS4 Coexpression,1213;Enrichr Queries,549;GTEx Coexpression,341	EBF1,ETS1
751	TFE3	ARCHS4 Coexpression,491;Enrichr Queries,838;GTEx Coexpression,775	ETS1,VEGFA
752	GBX1	ARCHS4 Coexpression,592;Enrichr Queries,506;GTEx Coexpression,1007	EBF1,NR2F2
753	ZNF800	ARCHS4 Coexpression,92;Enrichr Queries,808;GTEx Coexpression,1208	DDX3X,ETS1
754	SPIB	ARCHS4 Coexpression,1060;Enrichr Queries,853;ReMap ChIP-seq,61;GTEx Coexpression,838	EBF1,ETS1
755	NFIX	ARCHS4 Coexpression,1349;Enrichr Queries,107;GTEx Coexpression,653	EBF1,NR2F2,ETS1,VEGFA
756	TIGD2	ARCHS4 Coexpression,1133;Enrichr Queries,662;GTEx Coexpression,321	EFNB2,VEGFA
757	POGK	ARCHS4 Coexpression,19;Enrichr Queries,656;GTEx Coexpression,1443	EFNB2,FUS,NONO,ETS1
758	ST18	ARCHS4 Coexpression,818;Enrichr Queries,1029;GTEx Coexpression,273	EBF1
759	ZNF35	ARCHS4 Coexpression,957;Enrichr Queries,408;GTEx Coexpression,758	EFNB2,ETS1,VEGFA
760	ZNF326	ARCHS4 Coexpression,1207;Enrichr Queries,203;GTEx Coexpression,713	DDX3X,FUS,EWSR1,NONO
761	LHX3	ARCHS4 Coexpression,620;Enrichr Queries,514;GTEx Coexpression,990	EBF1,NR2F2
762	RFX2	ARCHS4 Coexpression,498;Enrichr Queries,720;ReMap ChIP-seq,271;GTEx Coexpression,1344	ETS1,VEGFA
763	ISL2	ARCHS4 Coexpression,1069;Enrichr Queries,220;GTEx Coexpression,837	EBF1,NR2F2,VEGFA
764	CDX4	ARCHS4 Coexpression,393;Enrichr Queries,923;GTEx Coexpression,812	NR2F2
765	FOXJ1	ARCHS4 Coexpression,659;Enrichr Queries,971;GTEx Coexpression,499	EFNB2
766	ZNF426	ARCHS4 Coexpression,962;Enrichr Queries,414;GTEx Coexpression,756	EFNB2,ETS1,VEGFA
767	ZNF648	ARCHS4 Coexpression,1057;Enrichr Queries,827;GTEx Coexpression,251	EBF1,NR2F2
768	LHX2	ARCHS4 Coexpression,1196;Enrichr Queries,258;ReMap ChIP-seq,64;GTEx Coexpression,1335	EFNB2,DDX3X,EBF1,NR2F2
769	PLAG1	ARCHS4 Coexpression,1294;Enrichr Queries,7;GTEx Coexpression,839	EFNB2,EBF1,NR2F2,ETS1,VEGFA
770	TP73	ARCHS4 Coexpression,509;Enrichr Queries,605;GTEx Coexpression,1028	ETS1,VEGFA
771	ZNF674	ARCHS4 Coexpression,1386;Enrichr Queries,213;GTEx Coexpression,551	NONO,PGK1,VEGFA
772	ZNF213	ARCHS4 Coexpression,396;Enrichr Queries,1211;GTEx Coexpression,546	
773	POU3F4	ARCHS4 Coexpression,261;Enrichr Queries,742;GTEx Coexpression,1151	EBF1,NR2F2
774	ZNF214	ARCHS4 Coexpression,93;Enrichr Queries,926;GTEx Coexpression,1135	PGK1,NR2F2
775	ZNF491	ARCHS4 Coexpression,808;Enrichr Queries,778;GTEx Coexpression,570	EFNB2,NR2F2
776	CREB3L1	ARCHS4 Coexpression,1597;Enrichr Queries,261;ReMap ChIP-seq,68;GTEx Coexpression,956	EFNB2,ETS1,VEGFA
777	ZNF277	ARCHS4 Coexpression,102;Enrichr Queries,741;GTEx Coexpression,1320	FUS,PGK1,VEGFA
778	ARNTL2	ARCHS4 Coexpression,596;Enrichr Queries,710;GTEx Coexpression,858	ETS1,VEGFA
779	ZNF266	ARCHS4 Coexpression,1442;Enrichr Queries,219;GTEx Coexpression,503	EFNB2,ETS1,VEGFA
780	CEBPE	ARCHS4 Coexpression,778;Enrichr Queries,1148;GTEx Coexpression,240	ETS1

781	HIF3A	ARCHS4 Coexpression,804;Enrichr Queries,301;GTEx Coexpression,1061	NR2F2,ETS1,VEGFA
782	IKZF4	ARCHS4 Coexpression,877;Enrichr Queries,713;GTEx Coexpression,578	ETS1,VEGFA
783	FOXS1	ARCHS4 Coexpression,111;Enrichr Queries,538;GTEx Coexpression,1520	EBF1,NR2F2
784	MYRFL	ARCHS4 Coexpression,398;GTEx Coexpression,1048	
785	GPBP1	ARCHS4 Coexpression,1134;GTEx Coexpression,313	
786	NOBOX	ARCHS4 Coexpression,701;Enrichr Queries,917;GTEx Coexpression,553	EBF1
787	NKX12	ARCHS4 Coexpression,812;GTEx Coexpression,636	
788	ZNF227	ARCHS4 Coexpression,91;Enrichr Queries,887;GTEx Coexpression,1197	NONO,VEGFA
789	ZNF114	ARCHS4 Coexpression,1293;Enrichr Queries,712;GTEx Coexpression,171	ETS1,VEGFA
790	KLF9	ARCHS4 Coexpression,1622;Enrichr Queries,1001;ReMap ChIP-seq,79;GTEx Coexpression,201	EBF1,VEGFA
791	SOX12	ARCHS4 Coexpression,209;Enrichr Queries,433;GTEx Coexpression,1536	EFNB2,FUS,NR2F2,VEGFA
792	PHOX2B	ARCHS4 Coexpression,613;Enrichr Queries,254;GTEx Coexpression,1311	EBF1,NR2F2,ETS1
793	ZSCAN5C	ARCHS4 Coexpression,835;Enrichr Queries,1193;GTEx Coexpression,153	PGK1
794	FOXD4L1	ARCHS4 Coexpression,1029;Enrichr Queries,569;GTEx Coexpression,584	NR2F2,VEGFA
795	POU2AF1	ARCHS4 Coexpression,1259;GTEx Coexpression,197	PGK1
796	ZNF516	ARCHS4 Coexpression,950;Enrichr Queries,195;GTEx Coexpression,1041	EFNB2,NR2F2,ETS1,VEGFA
797	MAFA	ARCHS4 Coexpression,552;Enrichr Queries,340;GTEx Coexpression,1297	EFNB2,NR2F2,VEGFA
798	KLF10	ARCHS4 Coexpression,952;Enrichr Queries,417;GTEx Coexpression,823	EFNB2,ETS1,VEGFA
799	ZNF496	ARCHS4 Coexpression,71;Enrichr Queries,1045;GTEx Coexpression,1077	FUS,NONO,VEGFA
800	MEOX1	ARCHS4 Coexpression,1626;Enrichr Queries,535;GTEx Coexpression,37	EBF1,ETS1
801	GZF1	ARCHS4 Coexpression,281;Enrichr Queries,833;GTEx Coexpression,1088	DDX3X,ETS1,VEGFA
802	ZNF254	ARCHS4 Coexpression,1405;Enrichr Queries,665;GTEx Coexpression,132	EFNB2,NR2F2,VEGFA
803	ZNF385D	ARCHS4 Coexpression,1054;Enrichr Queries,843;GTEx Coexpression,306	EBF1,ETS1
804	THAP7	ARCHS4 Coexpression,539;Enrichr Queries,1202;GTEx Coexpression,462	
805	FOXE3	ARCHS4 Coexpression,59;Enrichr Queries,867;GTEx Coexpression,1278	FUS,NONO,NR2F2
806	ZNF3	ARCHS4 Coexpression,859;Enrichr Queries,349;GTEx Coexpression,996	EFNB2,ETS1,VEGFA
807	POU5F1B	ARCHS4 Coexpression,473;GTEx Coexpression,997	
808	ISL1	ARCHS4 Coexpression,612;Enrichr Queries,100;GTEx Coexpression,1494	EFNB2,EBF1,NR2F2,ETS1
809	JRK	ARCHS4 Coexpression,243;GTEx Coexpression,1233	FUS
810	HMG20B	ARCHS4 Coexpression,590;Enrichr Queries,1219;GTEx Coexpression,407	
811	WIZ	ARCHS4 Coexpression,157;Enrichr Queries,1041;GTEx Coexpression,1018	FUS
812	ZFP37	ARCHS4 Coexpression,1593;Enrichr Queries,271;GTEx Coexpression,354	EFNB2,NR2F2,VEGFA
813	ZNF764	ARCHS4 Coexpression,466;Enrichr Queries,1346;GTEx Coexpression,409	
814	FOXD4L4	ARCHS4 Coexpression,489;GTEx Coexpression,992	
815	ZNF148	ARCHS4 Coexpression,108;Enrichr Queries,782;GTEx Coexpression,1333	DDX3X,FUS,ETS1
816	ZNF146	ARCHS4 Coexpression,220;Enrichr Queries,974;GTEx Coexpression,1031	DDX3X,NONO
817	GCM2	ARCHS4 Coexpression,401;Enrichr Queries,879;GTEx Coexpression,946	EBF1
818	NCOA2	ARCHS4 Coexpression,1016;Enrichr Queries,1079;ReMap ChIP-seq,217;GTEx Coexpression,657	VEGFA
819	XPA	ARCHS4 Coexpression,573;Enrichr Queries,358;GTEx Coexpression,1300	NR2F2,ETS1,VEGFA
820	TERF1	ARCHS4 Coexpression,508;Enrichr Queries,947;ReMap ChIP-seq,242;GTEx Coexpression,1279	VEGFA
821	ZNF205	ARCHS4 Coexpression,616;Enrichr Queries,1264;GTEx Coexpression,355	
822	RBPJL	ARCHS4 Coexpression,1305;Enrichr Queries,536;GTEx Coexpression,394	ETS1,VEGFA
823	PHF21A	ARCHS4 Coexpression,769;Enrichr Queries,1098;GTEx Coexpression,369	VEGFA
824	ZNF483	ARCHS4 Coexpression,506;Enrichr Queries,1217;GTEx Coexpression,518	
825	NFE2L3	ARCHS4 Coexpression,285;Enrichr Queries,719;GTEx Coexpression,1237	FUS,ETS1,VEGFA
826	ZNF761	ARCHS4 Coexpression,1299;Enrichr Queries,896;GTEx Coexpression,51	NONO,VEGFA
827	ZNF101	ARCHS4 Coexpression,1036;Enrichr Queries,871;GTEx Coexpression,339	ETS1
828	HOXA13	ARCHS4 Coexpression,591;Enrichr Queries,540;GTEx Coexpression,1116	EBF1,NR2F2
829	GATAD2A	ARCHS4 Coexpression,202;Enrichr Queries,776;GTEx Coexpression,1269	DDX3X,FUS,NONO
830	CDX1	ARCHS4 Coexpression,737;Enrichr Queries,984;GTEx Coexpression,528	NR2F2
831	DOT1L	ARCHS4 Coexpression,279;Enrichr Queries,1149;GTEx Coexpression,822	FUS,VEGFA
832	HOXA11	ARCHS4 Coexpression,322;Enrichr Queries,385;GTEx Coexpression,1544	EBF1,NR2F2,ETS1
833	RORB	ARCHS4 Coexpression,752;Enrichr Queries,167;GTEx Coexpression,1332	EFNB2,EBF1,NR2F2,VEGFA
834	ZBTB12	ARCHS4 Coexpression,33;Enrichr Queries,667;GTEx Coexpression,1551	FUS,NONO,NR2F2,VEGFA
835	ZZZ3	ARCHS4 Coexpression,1598;ENCODE ChIP-seq,116;Enrichr Queries,1091;ReMap ChIP-seq,2;GTEx Coexpression,952	DDX3X,NONO,EBF1
836	ZFP3	ARCHS4 Coexpression,493;Enrichr Queries,705;GTEx Coexpression,1059	EFNB2,ETS1
837	ZNF276	ARCHS4 Coexpression,165;Enrichr Queries,1140;GTEx Coexpression,958	ETS1,VEGFA
838	NR0B1	Literature ChIP-seq,100;ARCHS4 Coexpression,488;Enrichr Queries,893;GTEx Coexpression,1537	PGK1,VEGFA
839	CREB3L3	ARCHS4 Coexpression,534;Enrichr Queries,1276;GTEx Coexpression,455	
840	GTF3A	ARCHS4 Coexpression,387;Enrichr Queries,1082;GTEx Coexpression,797	PGK1
841	ZNF548	ARCHS4 Coexpression,1203;Enrichr Queries,1024;GTEx Coexpression,41	EBF1,ETS1,VEGFA
842	ZFPM1	ARCHS4 Coexpression,1160;Enrichr Queries,455;GTEx Coexpression,654	EFNB2,ETS1,VEGFA
843	DLX5	ARCHS4 Coexpression,1297;Enrichr Queries,274;GTEx Coexpression,701	EFNB2,EBF1,NR2F2
844	ZNF789	ARCHS4 Coexpression,1276;Enrichr Queries,973;GTEx Coexpression,24	FUS,EWSR1,VEGFA
845	CDC5L	ARCHS4 Coexpression,1561;Enrichr Queries,155;GTEx Coexpression,558	DDX3X,FUS,NONO,PGK1
846	NKX21	ARCHS4 Coexpression,1303;GTEx Coexpression,213	VEGFA
847	ZNF282	ARCHS4 Coexpression,275;Enrichr Queries,1081;GTEx Coexpression,919	FUS,VEGFA
848	GFI1	ARCHS4 Coexpression,236;Enrichr Queries,1163;GTEx Coexpression,877	ETS1
849	TEAD1	ARCHS4 Coexpression,1556;Enrichr Queries,130;ReMap ChIP-seq,89;GTEx Coexpression,1261	EFNB2,NR2F2,ETS1,VEGFA
850	HES5	ARCHS4 Coexpression,601;Enrichr Queries,165;GTEx Coexpression,1513	EFNB2,EBF1,NR2F2,VEGFA
851	NEUROG3	ARCHS4 Coexpression,362;Enrichr Queries,562;GTEx Coexpression,1357	EBF1,NR2F2
852	ZNF19	ARCHS4 Coexpression,967;Enrichr Queries,954;GTEx Coexpression,360	VEGFA
853	ZSCAN30	ARCHS4 Coexpression,1249;GTEx Coexpression,272	
854	SAFB2	ARCHS4 Coexpression,158;GTEx Coexpression,1365	FUS
855	ELF4	ARCHS4 Coexpression,743;Enrichr Queries,725;GTEx Coexpression,818	ETS1,VEGFA

856	SP5	ARCHS4 Coexpression,1301;Enrichr Queries,352;GTEx Coexpression,638	EFNB2,EBF1,NR2F2
857	RAX	ARCHS4 Coexpression,1559;Enrichr Queries,483;GTEx Coexpression,249	EBF1,NR2F2
858	ATOH7	ARCHS4 Coexpression,513;Enrichr Queries,906;GTEx Coexpression,873	NR2F2
859	HEYL	ARCHS4 Coexpression,624;Enrichr Queries,193;GTEx Coexpression,1477	EFNB2,EBF1,NR2F2,ETS1
860	CLOCK	Literature ChIP-seq,2;ARCHS4 Coexpression,976;Enrichr Queries,1161;ReMap ChIP-seq,226;GTEx Coexpression,1465	DDX3X,FUS,NR2F2,VEGFA
861	ETV3	ARCHS4 Coexpression,668;Enrichr Queries,850;GTEx Coexpression,781	ETS1,VEGFA
862	ZNF142	ARCHS4 Coexpression,167;Enrichr Queries,1205;GTEx Coexpression,927	FUS
863	ZIM3	ARCHS4 Coexpression,526;Enrichr Queries,1291;GTEx Coexpression,483	
864	NME2	ARCHS4 Coexpression,820;ReMap ChIP-seq,83;GTEx Coexpression,1399	VEGFA
865	EHF	ARCHS4 Coexpression,821;Enrichr Queries,1039;ReMap ChIP-seq,293;GTEx Coexpression,918	VEGFA
866	ZNF22	ARCHS4 Coexpression,1292;Enrichr Queries,913;GTEx Coexpression,100	ETS1
867	ZFP92	ARCHS4 Coexpression,1044;Enrichr Queries,1226;GTEx Coexpression,35	PGK1,ETS1
868	ZNF341	ARCHS4 Coexpression,588;Enrichr Queries,1170;GTEx Coexpression,548	NR2F2
869	ISX	ARCHS4 Coexpression,879;Enrichr Queries,1160;GTEx Coexpression,267	ETS1
870	ZNF549	ARCHS4 Coexpression,937;Enrichr Queries,878;GTEx Coexpression,493	ETS1
871	PRR12	ARCHS4 Coexpression,155;Enrichr Queries,1061;GTEx Coexpression,1093	FUS
872	ZNF124	ARCHS4 Coexpression,1339;Enrichr Queries,434;GTEx Coexpression,541	EFNB2,ETS1,VEGFA
873	ATF5	ARCHS4 Coexpression,887;Enrichr Queries,990;GTEx Coexpression,439	VEGFA
874	ZNF671	ARCHS4 Coexpression,748;Enrichr Queries,1035;GTEx Coexpression,535	ETS1
875	KLF8	ARCHS4 Coexpression,1121;Enrichr Queries,625;GTEx Coexpression,575	EFNB2,ETS1
876	NR1D2	ARCHS4 Coexpression,903;Enrichr Queries,595;GTEx Coexpression,825	ETS1,VEGFA
877	HOXB13	ARCHS4 Coexpression,368;Enrichr Queries,888;ReMap ChIP-seq,265;GTEx Coexpression,1578	NR2F2
878	MAFG	ARCHS4 Coexpression,900;Enrichr Queries,653;ReMap ChIP-seq,250;GTEx Coexpression,1298	ETS1,VEGFA
879	ZNF562	ARCHS4 Coexpression,1084;Enrichr Queries,812;GTEx Coexpression,431	ETS1,VEGFA
880	ZIC1	ARCHS4 Coexpression,1406;Enrichr Queries,255;GTEx Coexpression,668	EFNB2,EBF1,NR2F2
881	ZC3H8	ARCHS4 Coexpression,1272;Enrichr Queries,950;ReMap ChIP-seq,267;GTEx Coexpression,617	VEGFA
882	TEAD3	ARCHS4 Coexpression,966;Enrichr Queries,120;GTEx Coexpression,1245	EFNB2,NR2F2,ETS1,VEGFA
883	FOXJ2	ARCHS4 Coexpression,1258;Enrichr Queries,822;ReMap ChIP-seq,281;GTEx Coexpression,748	ETS1,VEGFA
884	FOXB2	ARCHS4 Coexpression,405;Enrichr Queries,472;GTEx Coexpression,1455	EBF1,NR2F2
885	SETDB2	ARCHS4 Coexpression,1171;Enrichr Queries,1151;GTEx Coexpression,11	EWSR1,NONO,VEGFA
886	ZNF74	ARCHS4 Coexpression,456;Enrichr Queries,698;GTEx Coexpression,1181	EFNB2,VEGFA
887	ZNF425	ARCHS4 Coexpression,958;Enrichr Queries,746;GTEx Coexpression,633	EFNB2,VEGFA
888	ZFP62	ARCHS4 Coexpression,1492;Enrichr Queries,394;GTEx Coexpression,451	DDX3X,ETS1,VEGFA
889	ZBTB38	ARCHS4 Coexpression,366;Enrichr Queries,648;GTEx Coexpression,1325	ETS1,VEGFA
890	FOXA3	ARCHS4 Coexpression,671;Enrichr Queries,1088;GTEx Coexpression,580	ETS1
891	ZNF224	ARCHS4 Coexpression,1169;Enrichr Queries,986;GTEx Coexpression,185	NONO,VEGFA
892	SKOR1	ARCHS4 Coexpression,878;GTEx Coexpression,684	
893	POU6F1	ARCHS4 Coexpression,1557;Enrichr Queries,402;GTEx Coexpression,385	EBF1,ETS1,VEGFA
894	MECOM	Literature ChIP-seq,106;ARCHS4 Coexpression,724;GTEx Coexpression,1515	VEGFA
895	BSX	ARCHS4 Coexpression,936;Enrichr Queries,205;GTEx Coexpression,1204	EBF1,NR2F2,ETS1
896	TBX5	Literature ChIP-seq,110;ARCHS4 Coexpression,1444;Enrichr Queries,359;GTEx Coexpression,1216	EBF1,NR2F2,ETS1
897	E2F2	ARCHS4 Coexpression,722;Enrichr Queries,1087;GTEx Coexpression,538	ETS1
898	ASCL4	ARCHS4 Coexpression,1062;Enrichr Queries,524;GTEx Coexpression,761	EBF1,NR2F2
899	TCF20	ARCHS4 Coexpression,725;Enrichr Queries,1224;GTEx Coexpression,400	
900	TFAP2D	ARCHS4 Coexpression,330;Enrichr Queries,550;GTEx Coexpression,1470	EBF1,NR2F2
901	ZNF554	ARCHS4 Coexpression,1000;Enrichr Queries,775;GTEx Coexpression,577	NR2F2,VEGFA
902	RHOXF2	ARCHS4 Coexpression,890;Enrichr Queries,150;GTEx Coexpression,1314	DDX3X,FUS,PGK1,ETS1
903	DMRT2	ARCHS4 Coexpression,414;Enrichr Queries,678;GTEx Coexpression,1262	EBF1,NR2F2
904	ZNF398	ARCHS4 Coexpression,719;Enrichr Queries,1232;GTEx Coexpression,403	
905	ZNF337	ARCHS4 Coexpression,623;Enrichr Queries,1169;GTEx Coexpression,563	VEGFA
906	ZNF790	ARCHS4 Coexpression,892;Enrichr Queries,1116;GTEx Coexpression,350	VEGFA
907	OLIG2	Literature ChIP-seq,42;ARCHS4 Coexpression,1413;Enrichr Queries,241;GTEx Coexpression,1449	EFNB2,EBF1,NR2F2,ETS1,VEGFA
908	ZNF540	ARCHS4 Coexpression,905;Enrichr Queries,1096;GTEx Coexpression,368	EBF1
909	HOXD1	ARCHS4 Coexpression,838;Enrichr Queries,238;GTEx Coexpression,1296	EFNB2,NR2F2,ETS1
910	NFE4	GTEx Coexpression,791	
911	MYF5	ARCHS4 Coexpression,619;Enrichr Queries,580;GTEx Coexpression,1185	EBF1,NR2F2
912	ZSCAN10	ARCHS4 Coexpression,278;Enrichr Queries,1253;GTEx Coexpression,854	NONO
913	TGIF1	ARCHS4 Coexpression,992;Enrichr Queries,752;GTEx Coexpression,643	ETS1,VEGFA
914	ZNF346	ARCHS4 Coexpression,1200;Enrichr Queries,1073;GTEx Coexpression,120	EFNB2,VEGFA
915	ZNF391	ARCHS4 Coexpression,1211;Enrichr Queries,953;GTEx Coexpression,232	VEGFA
916	ZFHX4	ARCHS4 Coexpression,537;Enrichr Queries,290;GTEx Coexpression,1572	EFNB2,EBF1,NR2F2
917	ZNF7	ARCHS4 Coexpression,961;Enrichr Queries,996;GTEx Coexpression,443	VEGFA
918	REL	ARCHS4 Coexpression,1281;Enrichr Queries,832;GTEx Coexpression,288	ETS1,VEGFA
919	ZBTB8A	ARCHS4 Coexpression,1537;GTEx Coexpression,65	EBF1
920	FOXD1	ARCHS4 Coexpression,132;GTEx Coexpression,1471	NR2F2
921	IRF7	ARCHS4 Coexpression,477;Enrichr Queries,1049;GTEx Coexpression,880	VEGFA
922	ZNF416	ARCHS4 Coexpression,164;Enrichr Queries,1257;GTEx Coexpression,985	NONO
923	ZNF486	ARCHS4 Coexpression,1348;Enrichr Queries,875;GTEx Coexpression,184	ETS1,VEGFA
924	GSC2	ARCHS4 Coexpression,556;Enrichr Queries,870;GTEx Coexpression,981	NR2F2
925	PREB	ARCHS4 Coexpression,428;Enrichr Queries,1164;GTEx Coexpression,815	PGK1
926	GPBP1L1	ARCHS4 Coexpression,852;GTEx Coexpression,753	
927	TET2	ARCHS4 Coexpression,1124;GTEx Coexpression,482	
928	CGGBP1	ARCHS4 Coexpression,76;GTEx Coexpression,1530	DDX3X,ETS1
929	ZNF510	ARCHS4 Coexpression,685;Enrichr Queries,1380;GTEx Coexpression,346	
930	ELF2	ARCHS4 Coexpression,1317;Enrichr Queries,790;GTEx Coexpression,305	DDX3X,ETS1

931	TBX20	ARCHS4 Coexpression,1611;Enrichr Queries,60;GTEx Coexpression,742	EFNB2,EBF1,NR2F2,ETS1
932	DBP	ARCHS4 Coexpression,871;Enrichr Queries,940;GTEx Coexpression,603	VEGFA
933	ZNF281	ARCHS4 Coexpression,1340;Enrichr Queries,329;GTEx Coexpression,745	DDX3X,ETS1,VEGFA
934	EVX1	ARCHS4 Coexpression,403;Enrichr Queries,473;GTEx Coexpression,1539	EBF1,NR2F2
935	TAL2	ARCHS4 Coexpression,1081;Enrichr Queries,722;GTEx Coexpression,612	EBF1,NR2F2
936	ZNF57	ARCHS4 Coexpression,1341;Enrichr Queries,517;GTEx Coexpression,560	ETS1,VEGFA
937	FERD3L	ARCHS4 Coexpression,517;Enrichr Queries,495;GTEx Coexpression,1407	EBF1,NR2F2
938	FOXI1	ARCHS4 Coexpression,776;Enrichr Queries,914;GTEx Coexpression,730	ETS1
939	TFDP3	ARCHS4 Coexpression,637;Enrichr Queries,809;GTEx Coexpression,978	NR2F2,ETS1
940	ZNF766	ARCHS4 Coexpression,1092;Enrichr Queries,1247;ReMap ChIP-seq,288;GTEx Coexpression,605	
941	MESP2	ARCHS4 Coexpression,991;Enrichr Queries,1201;GTEx Coexpression,236	
942	MECP2	ARCHS4 Coexpression,1500;Enrichr Queries,198;GTEx Coexpression,731	DDX3X,NR2F2,ETS1,VEGFA
943	SP110	ARCHS4 Coexpression,1147;Enrichr Queries,880;GTEx Coexpression,404	ETS1
944	HSFX2	ARCHS4 Coexpression,811	
945	VSX2	ARCHS4 Coexpression,516;Enrichr Queries,491;GTEx Coexpression,1426	EBF1,NR2F2
946	ZSCAN9	ARCHS4 Coexpression,1480;GTEx Coexpression,145	EWSR1
947	LBX2	ARCHS4 Coexpression,689;Enrichr Queries,1023;GTEx Coexpression,728	NR2F2
948	ZNF765	ARCHS4 Coexpression,997;Enrichr Queries,1239;GTEx Coexpression,205	NONO
949	ZNF646	ARCHS4 Coexpression,633;Enrichr Queries,1255;GTEx Coexpression,555	
950	ZNF776	ARCHS4 Coexpression,359;Enrichr Queries,1266;GTEx Coexpression,819	
951	DDIT3	ARCHS4 Coexpression,1256;Enrichr Queries,1030;GTEx Coexpression,159	VEGFA
952	CCDC17	ARCHS4 Coexpression,766;GTEx Coexpression,867	
953	ZNF451	ARCHS4 Coexpression,1353;Enrichr Queries,992;GTEx Coexpression,106	DDX3X,EWSR1
954	ZNF821	ARCHS4 Coexpression,515;Enrichr Queries,1159;GTEx Coexpression,777	VEGFA
955	RHOXF1	ARCHS4 Coexpression,833;Enrichr Queries,1122;GTEx Coexpression,496	EBF1
956	MXD1	ARCHS4 Coexpression,1127;Enrichr Queries,734;GTEx Coexpression,592	ETS1,VEGFA
957	ZSCAN21	ARCHS4 Coexpression,1099;Enrichr Queries,1027;GTEx Coexpression,328	VEGFA
958	THAP11	Literature ChIP-seq,85;ARCHS4 Coexpression,1574;Enrichr Queries,1186;ReMap ChIP-seq,287;GTEx Coex-pression,960	EWSR1
959	PROP1	ARCHS4 Coexpression,942;Enrichr Queries,499;GTEx Coexpression,1019	EBF1,NR2F2
960	ZNF442	ARCHS4 Coexpression,1440;Enrichr Queries,946;GTEx Coexpression,74	EBF1,VEGFA
961	NR1I3	ARCHS4 Coexpression,780;Enrichr Queries,1300;GTEx Coexpression,381	
962	ZNF20	ARCHS4 Coexpression,451;Enrichr Queries,798;GTEx Coexpression,1212	EFNB2,ETS1
963	DEAF1	ARCHS4 Coexpression,1588;Enrichr Queries,948;ReMap ChIP-seq,252;GTEx Coexpression,495	VEGFA
964	LBX1	ARCHS4 Coexpression,768;Enrichr Queries,474;GTEx Coexpression,1221	EBF1,NR2F2
965	HOXC8	ARCHS4 Coexpression,770;Enrichr Queries,110;GTEx Coexpression,1585	EFNB2,EBF1,NR2F2,ETS1
966	ZNF611	ARCHS4 Coexpression,1122;Enrichr Queries,440;GTEx Coexpression,903	EFNB2,NR2F2,ETS1
967	ZKSCAN7	ARCHS4 Coexpression,580;GTEx Coexpression,1066	
968	ZNF625	ARCHS4 Coexpression,1022;Enrichr Queries,1352;GTEx Coexpression,97	NONO
969	ZBED9	ARCHS4 Coexpression,802;GTEx Coexpression,848	
970	PURG	ARCHS4 Coexpression,943;GTEx Coexpression,707	
971	ZNF791	ARCHS4 Coexpression,1105;Enrichr Queries,598;GTEx Coexpression,773	DDX3X,VEGFA
972	SIX3	ARCHS4 Coexpression,1384;Enrichr Queries,70;GTEx Coexpression,1022	EFNB2,EBF1,NR2F2,VEGFA
973	ARX	ARCHS4 Coexpression,729;Enrichr Queries,302;GTEx Coexpression,1445	EBF1,NR2F2,ETS1
974	HIVEP3	ARCHS4 Coexpression,415;Enrichr Queries,1038;GTEx Coexpression,1025	ETS1
975	MLX	ARCHS4 Coexpression,681;Enrichr Queries,792;GTEx Coexpression,1005	PGK1,VEGFA
976	ZNF860	ARCHS4 Coexpression,1155;GTEx Coexpression,498	
977	PTF1A	ARCHS4 Coexpression,1308;Enrichr Queries,511;GTEx Coexpression,663	EBF1,NR2F2
978	FOXB1	ARCHS4 Coexpression,803;Enrichr Queries,490;GTEx Coexpression,1193	EBF1,NR2F2
979	ZNF804A	ARCHS4 Coexpression,569;Enrichr Queries,1071;GTEx Coexpression,847	EFNB2
980	ZNF735	ARCHS4 Coexpression,1019;GTEx Coexpression,641	
981	SCMH1	ARCHS4 Coexpression,273;GTEx Coexpression,1388	FUS
982	BPTF	ARCHS4 Coexpression,714;GTEx Coexpression,947	
983	ZNF585A	ARCHS4 Coexpression,1056;Enrichr Queries,925;GTEx Coexpression,513	VEGFA
984	NKX24	ARCHS4 Coexpression,673;GTEx Coexpression,991	
985	ZNF343	ARCHS4 Coexpression,745;Enrichr Queries,844;GTEx Coexpression,908	NR2F2,ETS1
986	HSFY1	ARCHS4 Coexpression,465;GTEx Coexpression,1200	
987	HMBOX1	ARCHS4 Coexpression,1221;Enrichr Queries,694;ReMap ChIP-seq,268;GTEx Coexpression,1149	ETS1,VEGFA
988	RBAK	ARCHS4 Coexpression,1364;Enrichr Queries,761;GTEx Coexpression,374	DDX3X,ETS1
989	VAX1	ARCHS4 Coexpression,665;Enrichr Queries,504;GTEx Coexpression,1331	EBF1,NR2F2
990	HDX	ARCHS4 Coexpression,1374;Enrichr Queries,1051;GTEx Coexpression,75	EBF1
991	GRHL1	ARCHS4 Coexpression,1542;Enrichr Queries,666;GTEx Coexpression,294	EFNB2,VEGFA
992	SIX6	ARCHS4 Coexpression,786;Enrichr Queries,505;GTEx Coexpression,1211	EBF1,NR2F2
993	HINFP	ARCHS4 Coexpression,1312;ReMap ChIP-seq,50;GTEx Coexpression,1141	NR2F2
994	ZNF449	ARCHS4 Coexpression,1591;Enrichr Queries,766;GTEx Coexpression,147	DDX3X,EBF1,VEGFA
995	HOXA10	ARCHS4 Coexpression,1257;Enrichr Queries,48;GTEx Coexpression,1199	EFNB2,EBF1,NR2F2,ETS1,VEGFA
996	E2F8	ARCHS4 Coexpression,687;Enrichr Queries,1314;GTEx Coexpression,505	
997	SNAI2	ARCHS4 Coexpression,1610;Enrichr Queries,89;ReMap ChIP-seq,225;GTEx Coexpression,1419	EFNB2,NR2F2,ETS1,VEGFA
998	ZNF264	ARCHS4 Coexpression,1047;Enrichr Queries,438;GTEx Coexpression,1024	EFNB2,ETS1,VEGFA
999	ZNF331	ARCHS4 Coexpression,507;Enrichr Queries,497;GTEx Coexpression,1507	ETS1,VEGFA
1000	REXO4	ARCHS4 Coexpression,250;GTEx Coexpression,1425	FUS
1001	ZNF667	ARCHS4 Coexpression,1079;Enrichr Queries,222;GTEx Coexpression,1213	EFNB2,ETS1,VEGFA
1002	MLXIPL	ARCHS4 Coexpression,922;Enrichr Queries,960;GTEx Coexpression,632	VEGFA
1003	ZNF189	ARCHS4 Coexpression,715;Enrichr Queries,904;GTEx Coexpression,899	VEGFA
1004	POU2F3	ARCHS4 Coexpression,1534;Enrichr Queries,675;GTEx Coexpression,312	EBF1,ETS1
1005	TCF24	ARCHS4 Coexpression,986;Enrichr Queries,1273;GTEx Coexpression,262	

1006	ZNF557	ARCHS4 Coexpression,1003;Enrichr Queries,458;GTEx Coexpression,1062	EFNB2,NR2F2,VEGFA
1007	ZNF131	ARCHS4 Coexpression,1452;Enrichr Queries,461;GTEx Coexpression,610	DDX3X,ETS1,VEGFA
1008	PRDM15	ARCHS4 Coexpression,1128;Enrichr Queries,1141;GTEx Coexpression,256	VEGFA
1009	TSC22D1	ARCHS4 Coexpression,1184;Enrichr Queries,315;GTEx Coexpression,1026	EFNB2,PGK1,VEGFA
1010	ZNF230	ARCHS4 Coexpression,744;Enrichr Queries,1105;GTEx Coexpression,679	EFNB2
1011	GMEB1	ARCHS4 Coexpression,1255;Enrichr Queries,412;GTEx Coexpression,861	FUS,ETS1,VEGFA
1012	L3MBTL1	ARCHS4 Coexpression,1198;GTEx Coexpression,488	
1013	ANKZF1	ARCHS4 Coexpression,98;Enrichr Queries,862;GTEx Coexpression,1573	PGK1,ETS1,VEGFA
1014	ZNF441	ARCHS4 Coexpression,700;Enrichr Queries,1047;GTEx Coexpression,788	VEGFA
1015	ZMAT4	ARCHS4 Coexpression,691;Enrichr Queries,1316;GTEx Coexpression,529	
1016	NFX1	ARCHS4 Coexpression,1014;Enrichr Queries,1158;GTEx Coexpression,365	FUS
1017	ZNF587B	ARCHS4 Coexpression,1429;GTEx Coexpression,263	
1018	ZNF236	ARCHS4 Coexpression,483;Enrichr Queries,1085;GTEx Coexpression,971	ETS1
1019	NR2E3	ARCHS4 Coexpression,1399;Enrichr Queries,294	EBF1,NR2F2,ETS1
1020	KCMF1	ARCHS4 Coexpression,576;Enrichr Queries,432;GTEx Coexpression,1532	DDX3X,PGK1,VEGFA
1021	LTF	ARCHS4 Coexpression,720;GTEx Coexpression,974	
1022	GTF2IRD2	ARCHS4 Coexpression,1024;Enrichr Queries,460;GTEx Coexpression,1057	NR2F2,ETS1,VEGFA
1023	ZBTB4	ARCHS4 Coexpression,1482;Enrichr Queries,669;GTEx Coexpression,391	ETS1,VEGFA
1024	ZNF710	ARCHS4 Coexpression,430;Enrichr Queries,1062;GTEx Coexpression,1052	NR2F2
1025	ZBTB7B	ARCHS4 Coexpression,806;Enrichr Queries,1131;GTEx Coexpression,607	VEGFA
1026	NKX23	ARCHS4 Coexpression,1460;GTEx Coexpression,238	
1027	ZNF83	ARCHS4 Coexpression,1345;Enrichr Queries,544;ReMap ChIP-seq,283;GTEx Coexpression,1232	ETS1,VEGFA
1028	ZNF595	ARCHS4 Coexpression,1174;Enrichr Queries,528	ETS1,VEGFA
1029	ZNF681	ARCHS4 Coexpression,938;Enrichr Queries,1206;GTEx Coexpression,411	
1030	TIGD7	ARCHS4 Coexpression,1402;Enrichr Queries,519;GTEx Coexpression,634	EFNB2,NR2F2
1031	ZNF594	ARCHS4 Coexpression,1614;Enrichr Queries,469;GTEx Coexpression,473	EFNB2,NR2F2,VEGFA
1032	SP9	ARCHS4 Coexpression,728;GTEx Coexpression,976	
1033	ZNF219	ARCHS4 Coexpression,391;Enrichr Queries,1283;GTEx Coexpression,883	
1034	MYNN	ARCHS4 Coexpression,1479;Enrichr Queries,1120;ReMap ChIP-seq,19;GTEx Coexpression,795	FUS,VEGFA
1035	ZNF688	ARCHS4 Coexpression,909;Enrichr Queries,872;GTEx Coexpression,780	VEGFA
1036	ZNF662	ARCHS4 Coexpression,1434;Enrichr Queries,886;GTEx Coexpression,244	VEGFA
1037	FOXI3	ARCHS4 Coexpression,1331;GTEx Coexpression,379	
1038	HES2	ARCHS4 Coexpression,885;Enrichr Queries,1034;GTEx Coexpression,646	EFNB2
1039	SOX10	ARCHS4 Coexpression,1538;Enrichr Queries,278;ReMap ChIP-seq,236;GTEx Coexpression,1376	NR2F2,ETS1,VEGFA
1040	ZNF543	ARCHS4 Coexpression,1624;Enrichr Queries,677;GTEx Coexpression,270	EFNB2,ETS1
1041	CAMTA1	ARCHS4 Coexpression,964;Enrichr Queries,780;GTEx Coexpression,827	NR2F2,ETS1
1042	MGA	ARCHS4 Coexpression,1499;Enrichr Queries,994;GTEx Coexpression,80	DDX3X,FUS
1043	ZNF607	ARCHS4 Coexpression,335;Enrichr Queries,1272;GTEx Coexpression,966	NONO
1044	TWIST1	ARCHS4 Coexpression,1548;Enrichr Queries,37;ReMap ChIP-seq,277;GTEx Coexpression,1570	EFNB2,EBF1,NR2F2,ETS1,VEGFA
1045	NFE2L1	ARCHS4 Coexpression,1335;Enrichr Queries,657;ReMap ChIP-seq,285;GTEx Coexpression,1157	PGK1,VEGFA
1046	ZNF692	ARCHS4 Coexpression,197;Enrichr Queries,1020;GTEx Coexpression,1359	FUS,VEGFA
1047	ZNF816	ARCHS4 Coexpression,1603;GTEx Coexpression,115	NONO
1048	ZNF660	ARCHS4 Coexpression,898;Enrichr Queries,682;GTEx Coexpression,999	EFNB2,EBF1
1049	NPAS4	ARCHS4 Coexpression,855;Enrichr Queries,1259;GTEx Coexpression,465	
1050	ARNT2	ARCHS4 Coexpression,1420;Enrichr Queries,276;GTEx Coexpression,884	EFNB2,NR2F2,ETS1
1051	ZNF841	ARCHS4 Coexpression,1010;Enrichr Queries,335;GTEx Coexpression,1236	EFNB2,ETS1,VEGFA
1052	VAX2	ARCHS4 Coexpression,1245;Enrichr Queries,509;GTEx Coexpression,828	EBF1,NR2F2
1053	DRAP1	ARCHS4 Coexpression,561;Enrichr Queries,961;GTEx Coexpression,1064	PGK1
1054	ZBTB25	ARCHS4 Coexpression,1435;Enrichr Queries,692;GTEx Coexpression,459	ETS1,VEGFA
1055	ESRRG	ARCHS4 Coexpression,433;Enrichr Queries,760;GTEx Coexpression,1397	EBF1,NR2F2
1056	ZSCAN22	ARCHS4 Coexpression,1152;Enrichr Queries,148;GTEx Coexpression,1290	EFNB2,NR2F2,ETS1,VEGFA
1057	THRB	ARCHS4 Coexpression,1304;Enrichr Queries,6;GTEx Coexpression,1282	EFNB2,EBF1,NR2F2,ETS1,VEGFA
1058	ZNF133	ARCHS4 Coexpression,474;Enrichr Queries,695;GTEx Coexpression,1424	EFNB2,VEGFA
1059	ZHX3	ARCHS4 Coexpression,1001;Enrichr Queries,429;GTEx Coexpression,1168	NR2F2,ETS1,VEGFA
1060	TIGD3	ARCHS4 Coexpression,544;Enrichr Queries,1110;GTEx Coexpression,945	EFNB2
1061	AEBP2	ARCHS4 Coexpression,1355;Enrichr Queries,277;GTEx Coexpression,968	DDX3X,ETS1,VEGFA
1062	ZNF676	ARCHS4 Coexpression,1511;Enrichr Queries,1021;GTEx Coexpression,68	EBF1,VEGFA
1063	DMTF1	ARCHS4 Coexpression,426;Enrichr Queries,661;GTEx Coexpression,1514	ETS1,VEGFA
1064	TRPS1	ARCHS4 Coexpression,1503;Enrichr Queries,156;GTEx Coexpression,944	EBF1,NR2F2,ETS1,VEGFA
1065	ZNF763	ARCHS4 Coexpression,646;Enrichr Queries,797;GTEx Coexpression,1162	EFNB2,ETS1
1066	ZNF541	ARCHS4 Coexpression,896;Enrichr Queries,1274;GTEx Coexpression,435	
1067	ZFHX2	ARCHS4 Coexpression,559;Enrichr Queries,1044;GTEx Coexpression,1003	VEGFA
1068	NR2C1	ARCHS4 Coexpression,1543;Enrichr Queries,424;GTEx Coexpression,640	NR2F2,ETS1,VEGFA
1069	ZSCAN20	ARCHS4 Coexpression,563;Enrichr Queries,859;GTEx Coexpression,1186	NR2F2,ETS1
1070	E4F1	Literature ChIP-seq,164;ARCHS4 Coexpression,930;Enrichr Queries,845;GTEx Coexpression,1543	ETS1,VEGFA
1071	ZNF785	ARCHS4 Coexpression,1400;GTEx Coexpression,342	
1072	HOXA5	ARCHS4 Coexpression,1020;Enrichr Queries,51;GTEx Coexpression,1547	EFNB2,EBF1,NR2F2,ETS1,VEGFA
1073	ZNF8	ARCHS4 Coexpression,1315;Enrichr Queries,431	EFNB2,ETS1,VEGFA
1074	DMRTA1	ARCHS4 Coexpression,1106;Enrichr Queries,369;GTEx Coexpression,1146	EBF1,NR2F2,ETS1
1075	ZNF827	ARCHS4 Coexpression,925;Enrichr Queries,596;GTEx Coexpression,1101	ETS1,VEGFA
1076	ZNF70	ARCHS4 Coexpression,1490;Enrichr Queries,793;GTEx Coexpression,340	EFNB2,NR2F2
1077	NRL	ARCHS4 Coexpression,977;Enrichr Queries,516;GTEx Coexpression,1131	EBF1,NR2F2
1078	TCF15	ARCHS4 Coexpression,693;Enrichr Queries,407;GTEx Coexpression,1525	EFNB2,EBF1,ETS1
1079	NR1H2	ARCHS4 Coexpression,978;Enrichr Queries,1234;ReMap ChIP-seq,17;GTEx Coexpression,1272	EFNB2,VEGFA
1080	ZFP2	ARCHS4 Coexpression,1191;Enrichr Queries,299;GTEx Coexpression,1137	EFNB2,EBF1,VEGFA
1081	ZNF665	ARCHS4 Coexpression,1226;Enrichr Queries,1340;GTEx Coexpression,61	NR2F2

1082	ZNF778	ARCHS4 Coexpression,1365;Enrichr Queries,1043;GTEx Coexpression,221	VEGFA
1083	ZNF813	ARCHS4 Coexpression,1470;GTEx Coexpression,283	
1084	ZBTB9	ARCHS4 Coexpression,190;Enrichr Queries,977;GTEx Coexpression,1463	NONO,VEGFA
1085	BHLHE41	ARCHS4 Coexpression,759;GTEx Coexpression,995	
1086	FOXI2	ARCHS4 Coexpression,1268;Enrichr Queries,534;GTEx Coexpression,831	EBF1,NR2F2
1087	ZBTB26	ARCHS4 Coexpression,21;Enrichr Queries,1228;GTEx Coexpression,1386	DDX3X,NONO
1088	ZNF461	ARCHS4 Coexpression,434;Enrichr Queries,1351;GTEx Coexpression,850	
1089	CREB3L2	ARCHS4 Coexpression,939;Enrichr Queries,140;GTEx Coexpression,1557	EFNB2,NR2F2,ETS1,VEGFA
1090	ZNF875	ARCHS4 Coexpression,817;Enrichr Queries,839;GTEx Coexpression,983	EFNB2,VEGFA
1091	GLIS2	ARCHS4 Coexpression,1528;Enrichr Queries,149;GTEx Coexpression,964	EFNB2,NR2F2,ETS1,VEGFA
1092	ZNF471	ARCHS4 Coexpression,1055;Enrichr Queries,976;GTEx Coexpression,611	EBF1
1093	ZNF696	ARCHS4 Coexpression,826;Enrichr Queries,857;GTEx Coexpression,962	ETS1,VEGFA
1094	ZNF18	ARCHS4 Coexpression,1033;Enrichr Queries,727;GTEx Coexpression,886	ETS1,VEGFA
1095	ZNF746	ARCHS4 Coexpression,394;Enrichr Queries,1338;GTEx Coexpression,915	
1096	MBNL2	ARCHS4 Coexpression,640;Enrichr Queries,613;GTEx Coexpression,1396	ETS1,VEGFA
1097	THAP6	ARCHS4 Coexpression,1168;Enrichr Queries,702;GTEx Coexpression,779	ETS1,VEGFA
1098	ZBTB45	ARCHS4 Coexpression,642;Enrichr Queries,1014;GTEx Coexpression,998	VEGFA
1099	MYT1L	ARCHS4 Coexpression,497;Enrichr Queries,1230;GTEx Coexpression,929	
1100	GSX1	ARCHS4 Coexpression,1093;Enrichr Queries,486;GTEx Coexpression,1079	EBF1,NR2F2
1101	ZNF354C	ARCHS4 Coexpression,1034;Enrichr Queries,386;GTEx Coexpression,1240	EBF1,NR2F2,ETS1
1102	OVOL1	ARCHS4 Coexpression,536;Enrichr Queries,1213;GTEx Coexpression,911	
1103	NPAS3	ARCHS4 Coexpression,840;Enrichr Queries,362;GTEx Coexpression,1458	EBF1,NR2F2,ETS1
1104	ZNF429	ARCHS4 Coexpression,1290;Enrichr Queries,1089;GTEx Coexpression,282	EFNB2
1105	ZNF787	ARCHS4 Coexpression,834;Enrichr Queries,1347;GTEx Coexpression,484	
1106	ZNF362	ARCHS4 Coexpression,1421;Enrichr Queries,292;GTEx Coexpression,953	NR2F2,ETS1,VEGFA
1107	ZNF333	ARCHS4 Coexpression,1123;Enrichr Queries,803;GTEx Coexpression,741	NR2F2,VEGFA
1108	GRHL3	ARCHS4 Coexpression,461;Enrichr Queries,1296;GTEx Coexpression,910	
1109	ZFP1	ARCHS4 Coexpression,973;Enrichr Queries,693;GTEx Coexpression,1002	EFNB2,VEGFA
1110	ZNF446	ARCHS4 Coexpression,602;Enrichr Queries,983;GTEx Coexpression,1083	EWSR1
1111	ZNF770	ARCHS4 Coexpression,1035;Enrichr Queries,908;GTEx Coexpression,725	VEGFA
1112	HLF	ARCHS4 Coexpression,1120;Enrichr Queries,583;GTEx Coexpression,965	EBF1,VEGFA
1113	ZSCAN31	ARCHS4 Coexpression,1246;GTEx Coexpression,533	
1114	FAM200B	ARCHS4 Coexpression,549;GTEx Coexpression,1231	
1115	HOMEZ	ARCHS4 Coexpression,849;Enrichr Queries,442;GTEx Coexpression,1383	NR2F2,ETS1,VEGFA
1116	ZNF385C	ARCHS4 Coexpression,994;Enrichr Queries,1240;GTEx Coexpression,440	
1117	DMRTC2	ARCHS4 Coexpression,518;Enrichr Queries,1374;GTEx Coexpression,783	
1118	CXXC1	ARCHS4 Coexpression,30;Enrichr Queries,1113;GTEx Coexpression,1533	FUS,EWSR1,VEGFA
1119	ZNF786	ARCHS4 Coexpression,847;Enrichr Queries,1395;GTEx Coexpression,436	
1120	HOXC12	ARCHS4 Coexpression,1319;Enrichr Queries,479;GTEx Coexpression,881	EBF1,NR2F2
1121	NANOGNB	ARCHS4 Coexpression,634;GTEx Coexpression,1154	
1122	NR5A1	ARCHS4 Coexpression,1023;Enrichr Queries,270;GTEx Coexpression,1391	NR2F2,ETS1,VEGFA
1123	ZNF71	ARCHS4 Coexpression,805;Enrichr Queries,805;GTEx Coexpression,1075	NR2F2,ETS1
1124	PAX4	ARCHS4 Coexpression,1026;Enrichr Queries,1003;GTEx Coexpression,660	ETS1
1125	ZNF440	ARCHS4 Coexpression,1175;Enrichr Queries,650;GTEx Coexpression,869	EFNB2,VEGFA
1126	ZMAT1	ARCHS4 Coexpression,425;Enrichr Queries,1046;GTEx Coexpression,1225	VEGFA
1127	ZSCAN16	ARCHS4 Coexpression,1530;Enrichr Queries,240;GTEx Coexpression,928	EFNB2,ETS1,VEGFA
1128	PRDM8	ARCHS4 Coexpression,980;Enrichr Queries,124;GTEx Coexpression,1597	EBF1,NR2F2,ETS1,VEGFA
1129	FOXH1	ARCHS4 Coexpression,565;Enrichr Queries,1072;GTEx Coexpression,1065	VEGFA
1130	ASCL2	ARCHS4 Coexpression,467;Enrichr Queries,1093;GTEx Coexpression,1144	ETS1
1131	ZNF347	ARCHS4 Coexpression,1275;Enrichr Queries,1185;GTEx Coexpression,247	
1132	ZNF30	ARCHS4 Coexpression,1158;Enrichr Queries,856;GTEx Coexpression,693	ETS1,VEGFA
1133	ZNF852	ARCHS4 Coexpression,1602;GTEx Coexpression,204	NONO
1134	ZNF430	ARCHS4 Coexpression,1145;Enrichr Queries,892;GTEx Coexpression,672	ETS1
1135	MTERF4	ARCHS4 Coexpression,323;GTEx Coexpression,1485	ETS1
1136	ZNF609	ARCHS4 Coexpression,217;Enrichr Queries,1177;GTEx Coexpression,1318	FUS
1137	ZNF600	ARCHS4 Coexpression,730;Enrichr Queries,873;GTEx Coexpression,1109	ETS1
1138	SNAPC2	ARCHS4 Coexpression,622;GTEx Coexpression,1187	
1139	ZNF280B	ARCHS4 Coexpression,1357;Enrichr Queries,453	NR2F2,ETS1,VEGFA
1140	CCDC169SO HLH2	ARCHS4 Coexpression,906	
1141	SALL3	ARCHS4 Coexpression,969;Enrichr Queries,273;GTEx Coexpression,1478	EFNB2,EBF1,NR2F2
1142	SIM2	ARCHS4 Coexpression,1208;Enrichr Queries,679;GTEx Coexpression,834	EBF1,NR2F2
1143	ZBTB22	ARCHS4 Coexpression,1144;Enrichr Queries,1016;GTEx Coexpression,562	VEGFA
1144	ZNF273	ARCHS4 Coexpression,1250;Enrichr Queries,1178;GTEx Coexpression,295	
1145	ZSCAN12	ARCHS4 Coexpression,1555;Enrichr Queries,1097;GTEx Coexpression,77	NONO,VEGFA
1146	MSGN1	ARCHS4 Coexpression,1524;Enrichr Queries,404;GTEx Coexpression,801	EBF1,NR2F2,ETS1
1147	ALX3	ARCHS4 Coexpression,914;Enrichr Queries,500;GTEx Coexpression,1319	EBF1,NR2F2
1148	DLX1	ARCHS4 Coexpression,1052;Enrichr Queries,312;GTEx Coexpression,1373	EFNB2,EBF1,NR2F2
1149	DLX6	ARCHS4 Coexpression,1058;Enrichr Queries,527;GTEx Coexpression,1152	EBF1,NR2F2
1150	NOTO	ARCHS4 Coexpression,883;Enrichr Queries,478;GTEx Coexpression,1377	EBF1,NR2F2
1151	ZNF473	ARCHS4 Coexpression,797;Enrichr Queries,895;GTEx Coexpression,1049	VEGFA
1152	ZNF420	ARCHS4 Coexpression,1360;Enrichr Queries,894;GTEx Coexpression,490	VEGFA
1153	PAX7	ARCHS4 Coexpression,924;Enrichr Queries,546;GTEx Coexpression,1275	EBF1,NR2F2
1154	ZNF32	ARCHS4 Coexpression,326;Enrichr Queries,910;GTEx Coexpression,1509	FUS,VEGFA
1155	ZNF284	ARCHS4 Coexpression,1409;Enrichr Queries,690;GTEx Coexpression,647	NR2F2,VEGFA
1156	TGIF2LX	ARCHS4 Coexpression,757;Enrichr Queries,405;GTEx Coexpression,1586	EBF1,NR2F2,ETS1

1157	ZNF200	ARCHS4 Coexpression,1172;Enrichr Queries,459;GTEx Coexpression,1117	EFNB2,ETS1,VEGFA
1158	ETV7	ARCHS4 Coexpression,453;Enrichr Queries,1404;GTEx Coexpression,895	
1159	SPZ1	ARCHS4 Coexpression,485;GTEx Coexpression,1351	
1160	ZNF781	ARCHS4 Coexpression,1244;Enrichr Queries,1080;GTEx Coexpression,434	EBF1
1161	ZNF512B	ARCHS4 Coexpression,137;Enrichr Queries,1138;GTEx Coexpression,1486	FUS,VEGFA
1162	ZNF579	ARCHS4 Coexpression,355;Enrichr Queries,1382;GTEx Coexpression,1027	NR2F2
1163	MYT1	ARCHS4 Coexpression,1235;Enrichr Queries,1136;GTEx Coexpression,395	EBF1
1164	MXD3	ARCHS4 Coexpression,872;Enrichr Queries,1203;GTEx Coexpression,696	
1165	SOX3	ARCHS4 Coexpression,794;Enrichr Queries,630;GTEx Coexpression,1348	EFNB2,NR2F2
1166	ZNF773	ARCHS4 Coexpression,1313;Enrichr Queries,1108;GTEx Coexpression,351	VEGFA
1167	TBX6	ARCHS4 Coexpression,709;Enrichr Queries,1166;GTEx Coexpression,900	VEGFA
1168	ZNF620	ARCHS4 Coexpression,1279;Enrichr Queries,1099;GTEx Coexpression,398	VEGFA
1169	ZNF135	ARCHS4 Coexpression,819;Enrichr Queries,754;GTEx Coexpression,1203	EBF1,ETS1
1170	THRA	Literature ChIP-seq,152;ARCHS4 Coexpression,1585;Enrichr Queries,607;GTEx Coexpression,1360	NR2F2,VEGFA
1171	ZNF699	ARCHS4 Coexpression,682;Enrichr Queries,608;GTEx Coexpression,1488	EFNB2,ETS1
1172	ZNF76	ARCHS4 Coexpression,195;Enrichr Queries,1055;GTEx Coexpression,1528	EWSR1,VEGFA
1173	ZNF576	ARCHS4 Coexpression,694;Enrichr Queries,989;GTEx Coexpression,1097	NR2F2
1174	ZKSCAN3	ARCHS4 Coexpression,1457;Enrichr Queries,1162;GTEx Coexpression,162	NONO,VEGFA
1175	THAP5	ARCHS4 Coexpression,1149;Enrichr Queries,1381;GTEx Coexpression,252	
1176	ZNF43	ARCHS4 Coexpression,1395;Enrichr Queries,1386;GTEx Coexpression,3	FUS,EWSR1,NONO
1177	FOXD4L6	ARCHS4 Coexpression,972;Enrichr Queries,884	NR2F2
1178	ZNF713	ARCHS4 Coexpression,1410;Enrichr Queries,701;GTEx Coexpression,677	EFNB2,NR2F2
1179	ZNF844	ARCHS4 Coexpression,1139;GTEx Coexpression,723	
1180	ZNF215	ARCHS4 Coexpression,1157;Enrichr Queries,771;GTEx Coexpression,868	ETS1,VEGFA
1181	NKX22	ARCHS4 Coexpression,832;GTEx Coexpression,1034	
1182	ZSCAN1	ARCHS4 Coexpression,423;Enrichr Queries,1121;GTEx Coexpression,1255	NR2F2
1183	ZNF296	ARCHS4 Coexpression,510;Enrichr Queries,1366;GTEx Coexpression,925	
1184	RORC	ARCHS4 Coexpression,933;Enrichr Queries,1033;GTEx Coexpression,836	VEGFA
1185	ZIK1	ARCHS4 Coexpression,1443;Enrichr Queries,949;GTEx Coexpression,412	EFNB2
1186	NR2E1	ARCHS4 Coexpression,1352;Enrichr Queries,67;GTEx Coexpression,1385	EFNB2,EBF1,NR2F2,VEGFA
1187	ZNF438	ARCHS4 Coexpression,1390;Enrichr Queries,988;GTEx Coexpression,429	ETS1
1188	HSF5	ARCHS4 Coexpression,658;Enrichr Queries,631;GTEx Coexpression,1519	EBF1,ETS1
1189	ZNF320	ARCHS4 Coexpression,1004;Enrichr Queries,738;GTEx Coexpression,1067	EFNB2,VEGFA
1190	INSM1	ARCHS4 Coexpression,891;Enrichr Queries,689;GTEx Coexpression,1230	EBF1,NR2F2
1191	ZUP1	ARCHS4 Coexpression,1477;Enrichr Queries,1165;GTEx Coexpression,173	PGK1,VEGFA
1192	ZNF134	ARCHS4 Coexpression,1527;Enrichr Queries,934;GTEx Coexpression,357	VEGFA
1193	ZFP69	ARCHS4 Coexpression,524;GTEx Coexpression,1355	
1194	FOXN4	ARCHS4 Coexpression,788;Enrichr Queries,1017;GTEx Coexpression,1015	NR2F2
1195	PRDM10	ARCHS4 Coexpression,1148;Enrichr Queries,788;GTEx Coexpression,887	ETS1,VEGFA
1196	TBX22	ARCHS4 Coexpression,427;Enrichr Queries,1354;GTEx Coexpression,1042	
1197	ZNF788P	ARCHS4 Coexpression,1190;Enrichr Queries,898;GTEx Coexpression,737	ETS1
1198	ZNF689	ARCHS4 Coexpression,614;Enrichr Queries,1078;GTEx Coexpression,1134	ETS1
1199	ZNF687	ARCHS4 Coexpression,331;Enrichr Queries,1192;GTEx Coexpression,1307	FUS
1200	ZNF573	ARCHS4 Coexpression,1565;Enrichr Queries,244;GTEx Coexpression,1021	NR2F2,ETS1,VEGFA
1201	SIM1	ARCHS4 Coexpression,706;Enrichr Queries,559;GTEx Coexpression,1568	EBF1,NR2F2
1202	ZNF575	ARCHS4 Coexpression,1454;Enrichr Queries,1315;GTEx Coexpression,64	DDX3X
1203	ZBTB49	ARCHS4 Coexpression,985;GTEx Coexpression,905	
1204	DMBX1	ARCHS4 Coexpression,1267;Enrichr Queries,223;GTEx Coexpression,1347	EFNB2,EBF1,NR2F2
1205	ZNF354A	ARCHS4 Coexpression,1389;Enrichr Queries,900;GTEx Coexpression,549	VEGFA
1206	ZBED1	ARCHS4 Coexpression,1346;Enrichr Queries,868;ReMap ChIP-seq,71;GTEx Coexpression,1502	FUS,PGK1
1207	ZNF574	ARCHS4 Coexpression,655;Enrichr Queries,1345;GTEx Coexpression,842	
1208	ZNF502	ARCHS4 Coexpression,773;Enrichr Queries,941;GTEx Coexpression,1129	ETS1
1209	ARID2	ARCHS4 Coexpression,1361;Enrichr Queries,1083;ReMap ChIP-seq,30;GTEx Coexpression,1323	DDX3X,NR2F2,VEGFA
1210	FIGLA	ARCHS4 Coexpression,1137;Enrichr Queries,498;GTEx Coexpression,1214	EBF1,NR2F2
1211	DPF1	ARCHS4 Coexpression,1311;Enrichr Queries,967;GTEx Coexpression,573	EFNB2
1212	RFX7	ARCHS4 Coexpression,897;Enrichr Queries,1058;GTEx Coexpression,896	ETS1
1213	ZNF891	ARCHS4 Coexpression,1138;GTEx Coexpression,765	
1214	LCORL	ARCHS4 Coexpression,1350;Enrichr Queries,1147;GTEx Coexpression,359	VEGFA
1215	TCF23	ARCHS4 Coexpression,1481;Enrichr Queries,1326;GTEx Coexpression,50	NR2F2
1216	ZNF511	ARCHS4 Coexpression,863;Enrichr Queries,1385;GTEx Coexpression,614	
1217	ZNF497	ARCHS4 Coexpression,383;Enrichr Queries,1371;GTEx Coexpression,1108	
1218	ZNF536	ARCHS4 Coexpression,747;Enrichr Queries,1010;GTEx Coexpression,1107	EBF1
1219	ZNF615	ARCHS4 Coexpression,1067;Enrichr Queries,999;GTEx Coexpression,799	VEGFA
1220	TMF1	ARCHS4 Coexpression,309;GTEx Coexpression,1601	FUS
1221	ZNF385A	ARCHS4 Coexpression,1261;Enrichr Queries,1074;GTEx Coexpression,531	VEGFA
1222	ZNF566	ARCHS4 Coexpression,1264;Enrichr Queries,1280;GTEx Coexpression,322	
1223	ZNF404	ARCHS4 Coexpression,1101;Enrichr Queries,903;GTEx Coexpression,864	VEGFA
1224	ATMIN	ARCHS4 Coexpression,1219;Enrichr Queries,372;GTEx Coexpression,1280	DDX3X,ETS1,VEGFA
1225	MSC	ARCHS4 Coexpression,726;Enrichr Queries,699;GTEx Coexpression,1450	ETS1,VEGFA
1226	ZNF41	ARCHS4 Coexpression,570;Enrichr Queries,1054;GTEx Coexpression,1251	DDX3X
1227	ZNF77	ARCHS4 Coexpression,1446;Enrichr Queries,779;GTEx Coexpression,651	EFNB2,ETS1
1228	ZNF79	ARCHS4 Coexpression,397;Enrichr Queries,1312;GTEx Coexpression,1169	
1229	OVOL3	ARCHS4 Coexpression,1063;GTEx Coexpression,856	
1230	ZNF582	ARCHS4 Coexpression,827;Enrichr Queries,1065;GTEx Coexpression,988	EBF1
1231	CIC	ARCHS4 Coexpression,1507;Enrichr Queries,1184;GTEx Coexpression,194	FUS
1232	IRX6	ARCHS4 Coexpression,1370;Enrichr Queries,229;GTEx Coexpression,1291	EFNB2,EBF1,NR2F2

1233	ZBTB20	ARCHS4 Coexpression,1225;Enrichr Queries,119;GTEx Coexpression,1546	EBF1,NR2F2,ETS1,VEGFA
1234	FOXN1	ARCHS4 Coexpression,663;Enrichr Queries,1344;GTEx Coexpression,885	
1235	ZNF705B	ARCHS4 Coexpression,449;GTEx Coexpression,1479	
1236	POU1F1	ARCHS4 Coexpression,1437;Enrichr Queries,305;GTEx Coexpression,1150	EBF1,NR2F2,ETS1
1237	ARID3B	ARCHS4 Coexpression,666;GTEx Coexpression,1263	
1238	FIZ1	ARCHS4 Coexpression,186;Enrichr Queries,1154;GTEx Coexpression,1559	FUS,VEGFA
1239	TEF	ARCHS4 Coexpression,1404;Enrichr Queries,956;GTEx Coexpression,540	VEGFA
1240	ZNF443	ARCHS4 Coexpression,1375;Enrichr Queries,623;GTEx Coexpression,906	EFNB2,ETS1
1241	DACH2	ARCHS4 Coexpression,531;Enrichr Queries,1128;GTEx Coexpression,1247	EBF1
1242	ZNF169	ARCHS4 Coexpression,935;Enrichr Queries,1322;GTEx Coexpression,656	
1243	ZNF329	ARCHS4 Coexpression,1560;Enrichr Queries,728;GTEx Coexpression,629	EFNB2,VEGFA
1244	ZNF493	ARCHS4 Coexpression,1141;Enrichr Queries,1324;GTEx Coexpression,457	
1245	ZXDC	ARCHS4 Coexpression,929;Enrichr Queries,783;GTEx Coexpression,1210	ETS1,VEGFA
1246	ZNF84	ARCHS4 Coexpression,1173;Enrichr Queries,556;GTEx Coexpression,1194	EFNB2,VEGFA
1247	ZNF585B	ARCHS4 Coexpression,824;Enrichr Queries,1006;GTEx Coexpression,1098	VEGFA
1248	ZNF197	ARCHS4 Coexpression,1465;Enrichr Queries,1379;ReMap ChIP-seq,261;GTEx Coexpression,800	
1249	RFX8	ARCHS4 Coexpression,472;GTEx Coexpression,1483	
1250	ZNF365	ARCHS4 Coexpression,578;Enrichr Queries,1313;GTEx Coexpression,1044	
1251	ZSCAN29	ARCHS4 Coexpression,1049;Enrichr Queries,1152;GTEx Coexpression,735	VEGFA
1252	ZSCAN5B	ARCHS4 Coexpression,1216;Enrichr Queries,1196;GTEx Coexpression,530	
1253	SKOR2	ARCHS4 Coexpression,1338;GTEx Coexpression,624	
1254	THAP8	ARCHS4 Coexpression,496;Enrichr Queries,1123;GTEx Coexpression,1326	VEGFA
1255	SPIC	ARCHS4 Coexpression,917;Enrichr Queries,1227;GTEx Coexpression,802	
1256	ZNF280A	ARCHS4 Coexpression,955;Enrichr Queries,584;GTEx Coexpression,1410	EFNB2,ETS1
1257	SOX15	ARCHS4 Coexpression,839;Enrichr Queries,1268;GTEx Coexpression,846	
1258	ZNF234	ARCHS4 Coexpression,1041;Enrichr Queries,1053;GTEx Coexpression,860	VEGFA
1259	ZNF628	ARCHS4 Coexpression,816;Enrichr Queries,1175;GTEx Coexpression,967	
1260	ZNF623	ARCHS4 Coexpression,697;Enrichr Queries,830;GTEx Coexpression,1431	ETS1,VEGFA
1261	ZNF564	ARCHS4 Coexpression,785;Enrichr Queries,1195;GTEx Coexpression,979	
1262	ZNF823	ARCHS4 Coexpression,1417;Enrichr Queries,826;GTEx Coexpression,717	EFNB2,ETS1
1263	ZFP90	ARCHS4 Coexpression,1002;Enrichr Queries,768;GTEx Coexpression,1190	EFNB2,ETS1
1264	ZNF335	ARCHS4 Coexpression,1045;Enrichr Queries,1176;ReMap ChIP-seq,297;GTEx Coexpression,1429	
1265	RHOXF2B	ARCHS4 Coexpression,801;Enrichr Queries,1144;GTEx Coexpression,1020	PGK1
1266	PPARA	ARCHS4 Coexpression,1170;Enrichr Queries,640;GTEx Coexpression,1156	ETS1,VEGFA
1267	ZNF518A	ARCHS4 Coexpression,1571;Enrichr Queries,1342;GTEx Coexpression,55	EWSR1
1268	ZNF706	ARCHS4 Coexpression,1209;Enrichr Queries,1057;GTEx Coexpression,703	PGK1
1269	ZNF749	ARCHS4 Coexpression,959;Enrichr Queries,1025;GTEx Coexpression,987	VEGFA
1270	ZFP42	Literature ChIP-seq,54;ARCHS4 Coexpression,1283;Enrichr Queries,1305;GTEx Coexpression,1322	EWSR1,ETS1
1271	ZNF678	ARCHS4 Coexpression,1496;Enrichr Queries,1102;GTEx Coexpression,377	NR2F2
1272	ZFP14	ARCHS4 Coexpression,1248;Enrichr Queries,1013;GTEx Coexpression,714	NR2F2
1273	ESX1	ARCHS4 Coexpression,1627;Enrichr Queries,226;GTEx Coexpression,1122	EBF1,NR2F2,ETS1
1274	ZNF208	ARCHS4 Coexpression,373;Enrichr Queries,1212;GTEx Coexpression,1392	
1275	ZNF283	ARCHS4 Coexpression,1422;Enrichr Queries,348;GTEx Coexpression,1209	EFNB2,ETS1,VEGFA
1276	ZNF485	ARCHS4 Coexpression,1327;Enrichr Queries,566;GTEx Coexpression,1087	ETS1,VEGFA
1277	ZNF695	ARCHS4 Coexpression,1407;Enrichr Queries,1358;GTEx Coexpression,215	PGK1
1278	ZNF385B	ARCHS4 Coexpression,881;Enrichr Queries,758;GTEx Coexpression,1342	EBF1,NR2F2
1279	DR1	ARCHS4 Coexpression,1590;Enrichr Queries,821;GTEx Coexpression,572	DDX3X,PGK1
1280	KLF15	ARCHS4 Coexpression,416;Enrichr Queries,963;GTEx Coexpression,1604	VEGFA
1281	ZNF529	ARCHS4 Coexpression,1066;Enrichr Queries,817;GTEx Coexpression,1100	EFNB2,ETS1
1282	ZNF610	ARCHS4 Coexpression,1287;Enrichr Queries,1187;GTEx Coexpression,510	
1283	ZNF285	ARCHS4 Coexpression,1553;GTEx Coexpression,437	
1284	ZNF93	ARCHS4 Coexpression,1581;Enrichr Queries,1208;GTEx Coexpression,198	PGK1
1285	ZNF771	ARCHS4 Coexpression,487;Enrichr Queries,1383;GTEx Coexpression,1119	
1286	ZNF843	ARCHS4 Coexpression,971;Enrichr Queries,1019;GTEx Coexpression,1000	NR2F2
1287	ZNF184	ARCHS4 Coexpression,1201;Enrichr Queries,436;GTEx Coexpression,1354	EFNB2,NR2F2,ETS1
1288	ZNF780A	ARCHS4 Coexpression,1077;Enrichr Queries,909;GTEx Coexpression,1011	VEGFA
1289	ZNF644	ARCHS4 Coexpression,1536;Enrichr Queries,897;ReMap ChIP-seq,222;GTEx Coexpression,1349	DDX3X
1290	ZNF616	ARCHS4 Coexpression,1567;Enrichr Queries,1360;GTEx Coexpression,76	NONO
1291	DRGX	ARCHS4 Coexpression,810;Enrichr Queries,609;GTEx Coexpression,1589	EBF1,NR2F2
1292	ZNF445	ARCHS4 Coexpression,1059;Enrichr Queries,1250;GTEx Coexpression,700	
1293	RBSN	ARCHS4 Coexpression,670;GTEx Coexpression,1338	
1294	ZNF250	ARCHS4 Coexpression,1298;Enrichr Queries,355;GTEx Coexpression,1361	EFNB2,ETS1,VEGFA
1295	ZNF138	ARCHS4 Coexpression,1509;Enrichr Queries,942;GTEx Coexpression,564	VEGFA
1296	CUX2	ARCHS4 Coexpression,577;Enrichr Queries,972;GTEx Coexpression,1468	EBF1
1297	ZNF195	ARCHS4 Coexpression,1401;Enrichr Queries,927;GTEx Coexpression,689	VEGFA
1298	ZNF324	ARCHS4 Coexpression,1167;Enrichr Queries,1183;GTEx Coexpression,667	
1299	ZNF677	ARCHS4 Coexpression,410;Enrichr Queries,1246;GTEx Coexpression,1362	
1300	LHX8	ARCHS4 Coexpression,990;Enrichr Queries,568;GTEx Coexpression,1462	EBF1,NR2F2
1301	ZNF784	ARCHS4 Coexpression,1205;Enrichr Queries,1397;GTEx Coexpression,419	
1302	BAZ2B	ARCHS4 Coexpression,867;Enrichr Queries,1200;GTEx Coexpression,957	
1303	ZBTB48	ARCHS4 Coexpression,734;Enrichr Queries,1167;GTEx Coexpression,1124	VEGFA
1304	NKX31	ARCHS4 Coexpression,543;GTEx Coexpression,1475	
1305	SPDEF	ARCHS4 Coexpression,514;Enrichr Queries,1005;GTEx Coexpression,1511	ETS1
1306	EVX2	ARCHS4 Coexpression,1011;Enrichr Queries,476;GTEx Coexpression,1545	EBF1,NR2F2
1307	HESX1	ARCHS4 Coexpression,913;Enrichr Queries,1243;GTEx Coexpression,876	
1308	CAMTA2	ARCHS4 Coexpression,1451;Enrichr Queries,1156;GTEx Coexpression,426	VEGFA

1309	ZNF500	ARCHS4 Coexpression,1575;Enrichr Queries,1278;GTEx Coexpression,180	NR2F2
1310	SKIL	ARCHS4 Coexpression,1445;Enrichr Queries,184;GTEx Coexpression,1406	EFNB2,DDX3X,ETS1,VEGFA
1311	ZNF556	ARCHS4 Coexpression,1396;Enrichr Queries,932;GTEx Coexpression,708	EFNB2
1312	ZNF48	ARCHS4 Coexpression,301;Enrichr Queries,1290;GTEx Coexpression,1446	FUS
1313	THAP3	ARCHS4 Coexpression,555;Enrichr Queries,982;GTEx Coexpression,1505	VEGFA
1314	TBX19	ARCHS4 Coexpression,1187;Enrichr Queries,674;GTEx Coexpression,1183	ETS1,VEGFA
1315	ZNF407	ARCHS4 Coexpression,1589;Enrichr Queries,1222;GTEx Coexpression,235	
1316	TTF1	ARCHS4 Coexpression,1239;GTEx Coexpression,792	
1317	ZNF560	ARCHS4 Coexpression,1230;Enrichr Queries,1248;GTEx Coexpression,569	
1318	TBX10	ARCHS4 Coexpression,656;Enrichr Queries,1331;GTEx Coexpression,1060	
1319	ZNF683	ARCHS4 Coexpression,690;Enrichr Queries,869;GTEx Coexpression,1489	ETS1
1320	ZNF730	ARCHS4 Coexpression,1558;GTEx Coexpression,475	
1321	ZNF613	ARCHS4 Coexpression,1453;Enrichr Queries,1242;GTEx Coexpression,358	
1322	ZNF99	ARCHS4 Coexpression,1487;Enrichr Queries,1179;GTEx Coexpression,392	
1323	TBPL2	ARCHS4 Coexpression,1552;GTEx Coexpression,487	
1324	ZNF865	ARCHS4 Coexpression,1130;GTEx Coexpression,909	
1325	MYF6	ARCHS4 Coexpression,643;Enrichr Queries,1330;GTEx Coexpression,1094	
1326	ZSCAN2	ARCHS4 Coexpression,1578;Enrichr Queries,376;GTEx Coexpression,1118	NR2F2,ETS1,VEGFA
1327	ZSCAN18	ARCHS4 Coexpression,1518;Enrichr Queries,829;GTEx Coexpression,726	EFNB2,VEGFA
1328	EMX1	ARCHS4 Coexpression,1146;Enrichr Queries,547;GTEx Coexpression,1380	EBF1,NR2F2
1329	ZNF547	ARCHS4 Coexpression,1159;Enrichr Queries,1251;GTEx Coexpression,664	
1330	ZSCAN5A	ARCHS4 Coexpression,1135;Enrichr Queries,635;GTEx Coexpression,1304	EFNB2,ETS1
1331	ZNF653	ARCHS4 Coexpression,1182;Enrichr Queries,1302;GTEx Coexpression,590	
1332	ZNF394	ARCHS4 Coexpression,1151;Enrichr Queries,1190;GTEx Coexpression,739	
1333	ZNF714	ARCHS4 Coexpression,1594;Enrichr Queries,1329;GTEx Coexpression,157	NONO
1334	JRKL	ARCHS4 Coexpression,699;Enrichr Queries,834;GTEx Coexpression,1549	ETS1,VEGFA
1335	ZNF454	ARCHS4 Coexpression,843;Enrichr Queries,1129;GTEx Coexpression,1114	EBF1
1336	ZNF707	ARCHS4 Coexpression,551;Enrichr Queries,1103;GTEx Coexpression,1432	VEGFA
1337	ZNF37A	ARCHS4 Coexpression,1252;Enrichr Queries,1237;GTEx Coexpression,600	
1338	ZNF419	ARCHS4 Coexpression,1087;Enrichr Queries,935;GTEx Coexpression,1068	VEGFA
1339	ZNF10	ARCHS4 Coexpression,988;Enrichr Queries,621;GTEx Coexpression,1482	EFNB2,VEGFA
1340	NR1D1	ARCHS4 Coexpression,1467;Enrichr Queries,938;GTEx Coexpression,687	VEGFA
1341	CREB3L4	ARCHS4 Coexpression,572;Enrichr Queries,1000;GTEx Coexpression,1521	VEGFA
1342	ZBTB47	ARCHS4 Coexpression,981;Enrichr Queries,610;GTEx Coexpression,1510	NR2F2,ETS1
1343	BATF2	ARCHS4 Coexpression,470;Enrichr Queries,1376;GTEx Coexpression,1256	
1344	ZNF98	ARCHS4 Coexpression,1247;Enrichr Queries,1214;GTEx Coexpression,642	
1345	ZNF235	ARCHS4 Coexpression,1605;Enrichr Queries,1066;GTEx Coexpression,432	VEGFA
1346	ZNF561	ARCHS4 Coexpression,1178;Enrichr Queries,919;GTEx Coexpression,1008	VEGFA
1347	ZNF286A	ARCHS4 Coexpression,1368;Enrichr Queries,574;GTEx Coexpression,1163	NR2F2,ETS1
1348	HSF2	ARCHS4 Coexpression,1262;Enrichr Queries,395;GTEx Coexpression,1451	NR2F2,ETS1,VEGFA
1349	ZNF414	ARCHS4 Coexpression,439;Enrichr Queries,1303;GTEx Coexpression,1366	
1350	ZNF431	ARCHS4 Coexpression,1118;Enrichr Queries,1225;GTEx Coexpression,768	
1351	ZNF225	ARCHS4 Coexpression,758;GTEx Coexpression,1317	
1352	ZNF639	ARCHS4 Coexpression,1136;Enrichr Queries,1197;ReMap ChIP-seq,275;GTEx Coexpression,1542	
1353	CXXC5	ARCHS4 Coexpression,1367;GTEx Coexpression,709	
1354	TFAP2E	ARCHS4 Coexpression,1512;Enrichr Queries,1223;GTEx Coexpression,380	
1355	ZNF507	ARCHS4 Coexpression,1210;Enrichr Queries,901;GTEx Coexpression,1010	ETS1
1356	ZNF619	ARCHS4 Coexpression,1197;Enrichr Queries,1297;GTEx Coexpression,628	
1357	CENPBD1	ARCHS4 Coexpression,963;GTEx Coexpression,1123	
1358	ZNF316	ARCHS4 Coexpression,941;Enrichr Queries,1064;GTEx Coexpression,1125	VEGFA
1359	ZNF44	ARCHS4 Coexpression,1008;Enrichr Queries,1060;GTEx Coexpression,1063	VEGFA
1360	ZNF396	ARCHS4 Coexpression,1204;Enrichr Queries,1301;GTEx Coexpression,626	
1361	TCFL5	ARCHS4 Coexpression,1617;Enrichr Queries,641;GTEx Coexpression,879	EFNB2,ETS1
1362	YBX2	ARCHS4 Coexpression,1076;Enrichr Queries,1028;GTEx Coexpression,1035	VEGFA
1363	ZNF222	ARCHS4 Coexpression,1415;Enrichr Queries,980;GTEx Coexpression,749	VEGFA
1364	ZNF726	ARCHS4 Coexpression,1398;GTEx Coexpression,699	
1365	ZNF672	ARCHS4 Coexpression,740;Enrichr Queries,1037;GTEx Coexpression,1374	VEGFA
1366	GSX2	ARCHS4 Coexpression,1180;Enrichr Queries,481;GTEx Coexpression,1492	EBF1,NR2F2
1367	PCGF6	ARCHS4 Coexpression,1266;Enrichr Queries,1112;GTEx Coexpression,776	VEGFA
1368	EBF4	ARCHS4 Coexpression,1423;Enrichr Queries,618;GTEx Coexpression,1113	EBF1,VEGFA
1369	ZNF121	ARCHS4 Coexpression,1623;Enrichr Queries,916;GTEx Coexpression,615	VEGFA
1370	ZNF518B	ARCHS4 Coexpression,1517;Enrichr Queries,192;GTEx Coexpression,1447	EFNB2,NR2F2,ETS1,VEGFA
1371	ZNF772	ARCHS4 Coexpression,1539;Enrichr Queries,819;GTEx Coexpression,806	EFNB2,VEGFA
1372	POU6F2	ARCHS4 Coexpression,606;Enrichr Queries,1134;GTEx Coexpression,1428	EBF1
1373	ZNF669	ARCHS4 Coexpression,1549;Enrichr Queries,1349;GTEx Coexpression,278	
1374	DPRX	ARCHS4 Coexpression,1394;Enrichr Queries,252;GTEx Coexpression,1531	EBF1,NR2F2,ETS1
1375	ZNF709	ARCHS4 Coexpression,1050;Enrichr Queries,1157;GTEx Coexpression,972	NR2F2
1376	CBLL2	ARCHS4 Coexpression,831;Enrichr Queries,755;GTEx Coexpression,1595	EBF1,ETS1
1377	ZNF239	ARCHS4 Coexpression,688;Enrichr Queries,1032;GTEx Coexpression,1466	VEGFA
1378	ZBTB17	ARCHS4 Coexpression,916;Enrichr Queries,861;GTEx Coexpression,1409	ETS1,VEGFA
1379	ZNF577	ARCHS4 Coexpression,993;Enrichr Queries,1258;GTEx Coexpression,936	
1380	ZNF439	ARCHS4 Coexpression,1150;Enrichr Queries,548;GTEx Coexpression,1491	EFNB2,ETS1
1381	ZNF257	ARCHS4 Coexpression,1086;Enrichr Queries,1100;GTEx Coexpression,1004	NR2F2
1382	AIRE	ARCHS4 Coexpression,408;Enrichr Queries,1368;GTEx Coexpression,1414	
1383	ZNF519	ARCHS4 Coexpression,974;Enrichr Queries,1359;GTEx Coexpression,859	
1384	ZFP91	ARCHS4 Coexpression,554;Enrichr Queries,1145;GTEx Coexpression,1497	DDX3X

1385	ZNF599	ARCHS4 Coexpression,692;Enrichr Queries,1070;GTEx Coexpression,1437	ETS1
1386	FOXR1	ARCHS4 Coexpression,1214;Enrichr Queries,1391;GTEx Coexpression,596	
1387	GLIS1	ARCHS4 Coexpression,1569;Enrichr Queries,227;GTEx Coexpression,1412	EBF1,NR2F2,ETS1
1388	RAX2	ARCHS4 Coexpression,908;Enrichr Queries,1363;GTEx Coexpression,938	
1389	ZNF768	ARCHS4 Coexpression,814;Enrichr Queries,891;GTEx Coexpression,1504	FUS
1390	ZNF23	ARCHS4 Coexpression,1322;Enrichr Queries,969;GTEx Coexpression,923	VEGFA
1391	FEZF2	ARCHS4 Coexpression,1347;Enrichr Queries,541;GTEx Coexpression,1328	EBF1,NR2F2
1392	SP6	ARCHS4 Coexpression,1253;Enrichr Queries,1281;GTEx Coexpression,682	
1393	ZNF132	ARCHS4 Coexpression,1114;Enrichr Queries,1235;GTEx Coexpression,878	
1394	ZNF668	ARCHS4 Coexpression,532;Enrichr Queries,1199;GTEx Coexpression,1499	
1395	ZNF501	ARCHS4 Coexpression,1455;Enrichr Queries,763;GTEx Coexpression,1016	EFNB2,VEGFA
1396	CPXCR1	ARCHS4 Coexpression,528;Enrichr Queries,1181;GTEx Coexpression,1526	
1397	FOXO4	ARCHS4 Coexpression,1234;Enrichr Queries,634;GTEx Coexpression,1370	ETS1,VEGFA
1398	ZNF383	ARCHS4 Coexpression,1025;Enrichr Queries,1052;GTEx Coexpression,1167	VEGFA
1399	ZNF879	ARCHS4 Coexpression,1393;GTEx Coexpression,774	
1400	GMEB2	ARCHS4 Coexpression,1156;Enrichr Queries,1063;GTEx Coexpression,1036	VEGFA
1401	TBPL1	ARCHS4 Coexpression,771;GTEx Coexpression,1402	
1402	ZNF408	ARCHS4 Coexpression,641;Enrichr Queries,1311;GTEx Coexpression,1309	
1403	ZNF799	ARCHS4 Coexpression,1366;Enrichr Queries,937;GTEx Coexpression,959	EFNB2
1404	ZNF728	ARCHS4 Coexpression,1132;GTEx Coexpression,1043	
1405	JAZF1	ARCHS4 Coexpression,1074;Enrichr Queries,614;GTEx Coexpression,1575	EBF1,ETS1
1406	POU5F2	ARCHS4 Coexpression,1096;GTEx Coexpression,1085	
1407	ZNF571	ARCHS4 Coexpression,1018;Enrichr Queries,1040;GTEx Coexpression,1219	VEGFA
1408	ZNF177	ARCHS4 Coexpression,1333;Enrichr Queries,912;GTEx Coexpression,1032	EFNB2
1409	MESP1	ARCHS4 Coexpression,1310;Enrichr Queries,899;GTEx Coexpression,1070	NR2F2
1410	ZSCAN26	ARCHS4 Coexpression,1325;GTEx Coexpression,863	
1411	ZNF382	ARCHS4 Coexpression,1282;Enrichr Queries,1378;GTEx Coexpression,623	
1412	ZNF506	ARCHS4 Coexpression,1274;Enrichr Queries,1004;GTEx Coexpression,1012	EFNB2
1413	MTERF3	ARCHS4 Coexpression,1320;GTEx Coexpression,874	
1414	TFEC	ARCHS4 Coexpression,736;Enrichr Queries,1245;GTEx Coexpression,1312	
1415	SNAPC5	ARCHS4 Coexpression,716;GTEx Coexpression,1480	
1416	MEF2D	ARCHS4 Coexpression,1188;Enrichr Queries,814;GTEx Coexpression,1293	ETS1,VEGFA
1417	ZNF630	ARCHS4 Coexpression,783;GTEx Coexpression,1415	
1418	SGSM2	ARCHS4 Coexpression,1428;GTEx Coexpression,771	
1419	HMX1	ARCHS4 Coexpression,1271;Enrichr Queries,477;GTEx Coexpression,1558	EBF1,NR2F2
1420	ZNF555	ARCHS4 Coexpression,911;Enrichr Queries,1126;GTEx Coexpression,1270	VEGFA
1421	TIGD5	ARCHS4 Coexpression,987;Enrichr Queries,1207;GTEx Coexpression,1115	
1422	ZNF705G	ARCHS4 Coexpression,711;GTEx Coexpression,1495	
1423	FEV	ARCHS4 Coexpression,923;Enrichr Queries,890;GTEx Coexpression,1498	EBF1
1424	ZNF606	ARCHS4 Coexpression,1032;Enrichr Queries,1084;GTEx Coexpression,1196	EFNB2
1425	ZNF287	ARCHS4 Coexpression,1143;Enrichr Queries,981;GTEx Coexpression,1191	NR2F2
1426	TBR1	ARCHS4 Coexpression,1176;Enrichr Queries,708;GTEx Coexpression,1433	EBF1,NR2F2
1427	MEIS3	ARCHS4 Coexpression,1582;Enrichr Queries,399;GTEx Coexpression,1337	EBF1,ETS1,VEGFA
1428	ZNF626	ARCHS4 Coexpression,463;Enrichr Queries,1357;GTEx Coexpression,1500	
1429	VENTX	ARCHS4 Coexpression,1153;Enrichr Queries,1369;GTEx Coexpression,798	
1430	ZFP57	ARCHS4 Coexpression,860;Enrichr Queries,1320;GTEx Coexpression,1142	
1431	NPAS1	ARCHS4 Coexpression,1065;Enrichr Queries,921;GTEx Coexpression,1341	VEGFA
1432	AHCTF1	ARCHS4 Coexpression,1291;Enrichr Queries,1101;GTEx Coexpression,935	DDX3X
1433	ZNF837	ARCHS4 Coexpression,750;Enrichr Queries,1403;GTEx Coexpression,1180	
1434	ZNF226	ARCHS4 Coexpression,1424;Enrichr Queries,837;GTEx Coexpression,1076	ETS1,VEGFA
1435	ZNF551	ARCHS4 Coexpression,1220;Enrichr Queries,939;GTEx Coexpression,1179	ETS1
1436	ZBTB18	ARCHS4 Coexpression,1529;GTEx Coexpression,698	
1437	ZNF268	ARCHS4 Coexpression,1206;Enrichr Queries,567;GTEx Coexpression,1569	NR2F2,VEGFA
1438	ZNF546	ARCHS4 Coexpression,1183;Enrichr Queries,1135;GTEx Coexpression,1029	ETS1
1439	RFX6	ARCHS4 Coexpression,629;Enrichr Queries,1279;GTEx Coexpression,1440	
1440	ZNF141	ARCHS4 Coexpression,1356;Enrichr Queries,1229;GTEx Coexpression,764	
1441	TPRX1	ARCHS4 Coexpression,1165;Enrichr Queries,1254;GTEx Coexpression,930	
1442	ZNF470	ARCHS4 Coexpression,1015;Enrichr Queries,1002;GTEx Coexpression,1339	VEGFA
1443	KLF17	ARCHS4 Coexpression,975;Enrichr Queries,933;GTEx Coexpression,1453	ETS1
1444	HES4	ARCHS4 Coexpression,1307;Enrichr Queries,492;GTEx Coexpression,1566	EFNB2,VEGFA
1445	PHF1	ARCHS4 Coexpression,1040;GTEx Coexpression,1205	
1446	FOXD4L5	ARCHS4 Coexpression,422;Enrichr Queries,1400;GTEx Coexpression,1550	
1447	TFDP2	ARCHS4 Coexpression,1164;Enrichr Queries,670;GTEx Coexpression,1538	ETS1,VEGFA
1448	SOHLH2	ARCHS4 Coexpression,946;Enrichr Queries,1009;GTEx Coexpression,1418	EFNB2
1449	ZFP64	ARCHS4 Coexpression,1473;Enrichr Queries,811;GTEx Coexpression,1089	NR2F2,ETS1
1450	PRMT3	ARCHS4 Coexpression,1048;Enrichr Queries,1241;GTEx Coexpression,1086	
1451	SEBOX	ARCHS4 Coexpression,1031;Enrichr Queries,1220	
1452	CARF	ARCHS4 Coexpression,1094;GTEx Coexpression,1158	
1453	ZNF729	ARCHS4 Coexpression,1215;GTEx Coexpression,1037	
1454	USF3	ARCHS4 Coexpression,1222;GTEx Coexpression,1030	
1455	ZNF586	ARCHS4 Coexpression,1475;Enrichr Queries,1090;GTEx Coexpression,816	ETS1
1456	ZFP82	ARCHS4 Coexpression,1562;Enrichr Queries,930;GTEx Coexpression,889	ETS1
1457	ZNF675	ARCHS4 Coexpression,1005;Enrichr Queries,1249;GTEx Coexpression,1130	
1458	ZNF91	ARCHS4 Coexpression,1163;Enrichr Queries,1377;GTEx Coexpression,845	
1459	AHDC1	ARCHS4 Coexpression,1510;Enrichr Queries,1289;GTEx Coexpression,586	
1460	ZNF621	ARCHS4 Coexpression,1318;Enrichr Queries,842;GTEx Coexpression,1227	ETS1,VEGFA

1461	ZNF474	ARCHS4 Coexpression,464;Enrichr Queries,1384;GTEx Coexpression,1541	
1462	ZNF627	ARCHS4 Coexpression,1576;Enrichr Queries,632;GTEx Coexpression,1184	EFNB2,ETS1
1463	GTF2IRD2B	ARCHS4 Coexpression,1277;Enrichr Queries,959;GTEx Coexpression,1160	VEGFA
1464	ZNF587	ARCHS4 Coexpression,1142;Enrichr Queries,966;GTEx Coexpression,1289	VEGFA
1465	ZBTB37	ARCHS4 Coexpression,1078;Enrichr Queries,1067;GTEx Coexpression,1252	VEGFA
1466	ZNF624	ARCHS4 Coexpression,1504;Enrichr Queries,555;GTEx Coexpression,1346	EFNB2,ETS1
1467	ZNF708	ARCHS4 Coexpression,1502;Enrichr Queries,1327;GTEx Coexpression,579	
1468	TERB1	ARCHS4 Coexpression,1551;GTEx Coexpression,722	
1469	ZBTB44	ARCHS4 Coexpression,1302;Enrichr Queries,816;GTEx Coexpression,1294	ETS1,VEGFA
1470	ZBTB41	ARCHS4 Coexpression,1515;Enrichr Queries,1236;GTEx Coexpression,665	
1471	ZNF181	ARCHS4 Coexpression,1377;Enrichr Queries,1355;GTEx Coexpression,686	
1472	ZNF570	ARCHS4 Coexpression,1456;Enrichr Queries,1262;GTEx Coexpression,704	
1473	ZNF517	ARCHS4 Coexpression,954;Enrichr Queries,1294;GTEx Coexpression,1176	
1474	ZNF490	ARCHS4 Coexpression,1448;Enrichr Queries,1194;GTEx Coexpression,793	
1475	ZNF567	ARCHS4 Coexpression,626;Enrichr Queries,1372;GTEx Coexpression,1438	
1476	LCOR	ARCHS4 Coexpression,1568;Enrichr Queries,786;GTEx Coexpression,1095	ETS1,VEGFA
1477	ZIM2	ARCHS4 Coexpression,707;Enrichr Queries,1142;GTEx Coexpression,1606	EBF1
1478	ZNF528	ARCHS4 Coexpression,901;Enrichr Queries,1287;GTEx Coexpression,1274	
1479	ZNF580	ARCHS4 Coexpression,1064;Enrichr Queries,965;GTEx Coexpression,1435	VEGFA
1480	MSANTD4	ARCHS4 Coexpression,746;GTEx Coexpression,1564	
1481	ZNF260	ARCHS4 Coexpression,1382;Enrichr Queries,711;GTEx Coexpression,1372	EFNB2,VEGFA
1482	ARGFX	ARCHS4 Coexpression,1103;Enrichr Queries,1231;GTEx Coexpression,1132	
1483	SOX30	ARCHS4 Coexpression,755;Enrichr Queries,1390;GTEx Coexpression,1330	
1484	ZNF492	ARCHS4 Coexpression,1573;GTEx Coexpression,754	
1485	KLF16	ARCHS4 Coexpression,1233;Enrichr Queries,1094;GTEx Coexpression,1170	VEGFA
1486	SP7	ARCHS4 Coexpression,1508;Enrichr Queries,944;GTEx Coexpression,1047	VEGFA
1487	ZNF527	ARCHS4 Coexpression,1430;Enrichr Queries,1332;GTEx Coexpression,746	
1488	ZKSCAN5	ARCHS4 Coexpression,1533;Enrichr Queries,924;GTEx Coexpression,1051	VEGFA
1489	BBX	ARCHS4 Coexpression,1587;Enrichr Queries,587;GTEx Coexpression,1334	ETS1,VEGFA
1490	ZNF680	ARCHS4 Coexpression,1441;Enrichr Queries,1036;GTEx Coexpression,1038	EFNB2
1491	ZNF526	ARCHS4 Coexpression,1376;Enrichr Queries,1180;GTEx Coexpression,961	
1492	ZNF775	ARCHS4 Coexpression,989;Enrichr Queries,1339;GTEx Coexpression,1198	
1493	NFXL1	ARCHS4 Coexpression,1185;Enrichr Queries,1107;GTEx Coexpression,1238	VEGFA
1494	ZNF808	ARCHS4 Coexpression,1329;Enrichr Queries,951;GTEx Coexpression,1253	ETS1
1495	GATA5	ARCHS4 Coexpression,1082;Enrichr Queries,889;GTEx Coexpression,1562	NR2F2
1496	ZNF155	ARCHS4 Coexpression,1459;Enrichr Queries,1387;GTEx Coexpression,694	
1497	ZNF418	ARCHS4 Coexpression,1359;Enrichr Queries,907;GTEx Coexpression,1283	EFNB2
1498	ZNF524	ARCHS4 Coexpression,1007;Enrichr Queries,1398;GTEx Coexpression,1147	
1499	ZNF280C	ARCHS4 Coexpression,1438;Enrichr Queries,750;GTEx Coexpression,1369	DDX3X,ETS1
1500	ZNF444	ARCHS4 Coexpression,1416;Enrichr Queries,1335;GTEx Coexpression,808	
1501	SLC2A4RG	ARCHS4 Coexpression,1043;Enrichr Queries,1393;GTEx Coexpression,1126	
1502	ZNF480	ARCHS4 Coexpression,1419;Enrichr Queries,1304;GTEx Coexpression,840	
1503	ZGLP1	ARCHS4 Coexpression,1522;GTEx Coexpression,855	
1504	ZNF721	ARCHS4 Coexpression,1478;Enrichr Queries,1334;GTEx Coexpression,755	
1505	ZNF484	ARCHS4 Coexpression,1608;Enrichr Queries,1298;GTEx Coexpression,666	
1506	ZNF718	ARCHS4 Coexpression,1621;Enrichr Queries,471;GTEx Coexpression,1481	NR2F2,ETS1,VEGFA
1507	ZSCAN23	ARCHS4 Coexpression,1520;Enrichr Queries,1042;GTEx Coexpression,1014	NR2F2
1508	ZNF705A	ARCHS4 Coexpression,830;Enrichr Queries,1396;GTEx Coexpression,1353	
1509	PEG3	ARCHS4 Coexpression,1100;GTEx Coexpression,1287	
1510	RXRB	ARCHS4 Coexpression,1238;Enrichr Queries,765;GTEx Coexpression,1582	PGK1,VEGFA
1511	MTERF2	ARCHS4 Coexpression,949;GTEx Coexpression,1442	
1512	ZNF345	ARCHS4 Coexpression,1104;Enrichr Queries,1282;GTEx Coexpression,1202	
1513	ZNF829	ARCHS4 Coexpression,953;Enrichr Queries,1271;GTEx Coexpression,1368	
1514	SOX8	ARCHS4 Coexpression,1595;Enrichr Queries,409;GTEx Coexpression,1588	EFNB2,NR2F2,VEGFA
1515	ZNF253	ARCHS4 Coexpression,1472;Enrichr Queries,1198;GTEx Coexpression,924	
1516	TGIF2LY	ARCHS4 Coexpression,1309;Enrichr Queries,866;GTEx Coexpression,1421	ETS1
1517	KIN	ARCHS4 Coexpression,1570;Enrichr Queries,1139;GTEx Coexpression,893	FUS
1518	NKX63	ARCHS4 Coexpression,1615;GTEx Coexpression,794	
1519	ZNF552	ARCHS4 Coexpression,1607;Enrichr Queries,1095;GTEx Coexpression,913	NR2F2
1520	ZNF684	ARCHS4 Coexpression,1088;Enrichr Queries,1307;GTEx Coexpression,1222	
1521	ZNF780B	ARCHS4 Coexpression,984;Enrichr Queries,1244;GTEx Coexpression,1393	
1522	ZNF81	ARCHS4 Coexpression,1545;Enrichr Queries,1309;GTEx Coexpression,770	
1523	THAP4	ARCHS4 Coexpression,893;Enrichr Queries,1265;GTEx Coexpression,1469	
1524	ZNF563	ARCHS4 Coexpression,772;Enrichr Queries,1260;GTEx Coexpression,1596	
1525	ZNF572	ARCHS4 Coexpression,934;Enrichr Queries,1118;GTEx Coexpression,1577	VEGFA
1526	ZNF836	ARCHS4 Coexpression,920;Enrichr Queries,1275;GTEx Coexpression,1441	
1527	KAT7	ARCHS4 Coexpression,1162;GTEx Coexpression,1268	
1528	ATF6B	ARCHS4 Coexpression,1326;GTEx Coexpression,1106	
1529	ZNF488	ARCHS4 Coexpression,1550;Enrichr Queries,883	EFNB2
1530	ZSCAN32	ARCHS4 Coexpression,1337;GTEx Coexpression,1096	
1531	NKX11	ARCHS4 Coexpression,1241;GTEx Coexpression,1192	
1532	ZNF614	ARCHS4 Coexpression,1354;GTEx Coexpression,1081	
1533	ZNF112	ARCHS4 Coexpression,1544;GTEx Coexpression,892	
1534	ZNF747	ARCHS4 Coexpression,1193;Enrichr Queries,1388;GTEx Coexpression,1074	
1535	ZNF649	ARCHS4 Coexpression,1411;Enrichr Queries,943;GTEx Coexpression,1302	EFNB2
1536	ZBTB8B	ARCHS4 Coexpression,1212;GTEx Coexpression,1229	

1537	CREBL2	ARCHS4 Coexpression,1381;Enrichr Queries,767;GTEx Coexpression,1517	ETS1,VEGFA
1538	ZNF605	ARCHS4 Coexpression,1263;Enrichr Queries,1008;GTEx Coexpression,1398	VEGFA
1539	ARHGAP35	ARCHS4 Coexpression,965;GTEx Coexpression,1487	
1540	ZNF397	ARCHS4 Coexpression,1195;GTEx Coexpression,1258	
1541	NEUROD6	ARCHS4 Coexpression,1288;Enrichr Queries,975;GTEx Coexpression,1417	NR2F2
1542	ZNF367	ARCHS4 Coexpression,1577;Enrichr Queries,1362;GTEx Coexpression,750	
1543	PROX2	ARCHS4 Coexpression,1296;Enrichr Queries,1086;GTEx Coexpression,1313	VEGFA
1544	L3MBTL4	ARCHS4 Coexpression,1546;GTEx Coexpression,921	
1545	ZNF433	ARCHS4 Coexpression,1358;Enrichr Queries,928;GTEx Coexpression,1420	EFNB2
1546	MKX	ARCHS4 Coexpression,1526;Enrichr Queries,590;GTEx Coexpression,1598	EFNB2,NR2F2
1547	ZNF845	ARCHS4 Coexpression,1586;Enrichr Queries,1364;GTEx Coexpression,772	
1548	HES6	ARCHS4 Coexpression,1115;Enrichr Queries,1367;GTEx Coexpression,1241	
1549	ZNF701	ARCHS4 Coexpression,1620;Enrichr Queries,1068;GTEx Coexpression,1039	EFNB2
1550	ZNF793	ARCHS4 Coexpression,1432;Enrichr Queries,1218;GTEx Coexpression,1082	
1551	ZNF716	ARCHS4 Coexpression,1369;Enrichr Queries,1150;GTEx Coexpression,1215	ETS1
1552	ZNF221	ARCHS4 Coexpression,1541;Enrichr Queries,1267;GTEx Coexpression,939	
1553	ZNF14	ARCHS4 Coexpression,1012;Enrichr Queries,1392;GTEx Coexpression,1352	
1554	ZNF830	ARCHS4 Coexpression,1166;Enrichr Queries,1204;GTEx Coexpression,1387	
1555	ZNF180	ARCHS4 Coexpression,1600;Enrichr Queries,1348;GTEx Coexpression,821	
1556	ZNF717	ARCHS4 Coexpression,1491;Enrichr Queries,1399;GTEx Coexpression,891	
1557	ZNF530	ARCHS4 Coexpression,1584;Enrichr Queries,1333;GTEx Coexpression,875	
1558	ZNF705D	ARCHS4 Coexpression,1181;Enrichr Queries,1299;GTEx Coexpression,1321	
1559	RBCK1	ARCHS4 Coexpression,951;GTEx Coexpression,1583	
1560	ZNF28	ARCHS4 Coexpression,1450;Enrichr Queries,813;GTEx Coexpression,1561	EFNB2,VEGFA
1561	TIGD6	ARCHS4 Coexpression,1628;Enrichr Queries,804;GTEx Coexpression,1405	ETS1,VEGFA
1562	ZNF487	ARCHS4 Coexpression,1314;Enrichr Queries,985;GTEx Coexpression,1553	VEGFA
1563	PHF20	ARCHS4 Coexpression,1408;Enrichr Queries,1189;GTEx Coexpression,1259	
1564	MYRF	ARCHS4 Coexpression,1223;GTEx Coexpression,1356	
1565	SOHLH1	ARCHS4 Coexpression,1316;Enrichr Queries,1069;GTEx Coexpression,1490	ETS1
1566	ZNF727	ARCHS4 Coexpression,1397;GTEx Coexpression,1189	
1567	YY2	ARCHS4 Coexpression,1612;Enrichr Queries,978;GTEx Coexpression,1308	EFNB2
1568	FOXR2	ARCHS4 Coexpression,1265;Enrichr Queries,1389;GTEx Coexpression,1250	
1569	ZNF835	ARCHS4 Coexpression,1540;Enrichr Queries,1261;GTEx Coexpression,1104	
1570	ZNF229	ARCHS4 Coexpression,1372;Enrichr Queries,1233	
1571	ZNF682	ARCHS4 Coexpression,1385;Enrichr Queries,1394;GTEx Coexpression,1139	
1572	ZBTB43	ARCHS4 Coexpression,1388;Enrichr Queries,998;GTEx Coexpression,1535	VEGFA
1573	ZNF154	ARCHS4 Coexpression,1194;Enrichr Queries,1209;GTEx Coexpression,1518	
1574	ZNF679	ARCHS4 Coexpression,1110;Enrichr Queries,1353;GTEx Coexpression,1460	
1575	SCRT1	ARCHS4 Coexpression,1486;Enrichr Queries,1130	EBF1
1576	ZNF700	ARCHS4 Coexpression,1462;Enrichr Queries,1221;GTEx Coexpression,1248	
1577	OLIG1	ARCHS4 Coexpression,1625;Enrichr Queries,915;GTEx Coexpression,1404	VEGFA
1578	ZNF182	ARCHS4 Coexpression,1334;Enrichr Queries,1050;GTEx Coexpression,1560	VEGFA
1579	BHLHA9	ARCHS4 Coexpression,1254;GTEx Coexpression,1381	
1580	ZNF211	ARCHS4 Coexpression,1380;Enrichr Queries,1114;GTEx Coexpression,1476	VEGFA
1581	ZNF140	ARCHS4 Coexpression,1433;Enrichr Queries,991;GTEx Coexpression,1548	VEGFA
1582	CENPT	ARCHS4 Coexpression,1229;Enrichr Queries,1365;GTEx Coexpression,1382	
1583	ZNF233	ARCHS4 Coexpression,1270;Enrichr Queries,1306;GTEx Coexpression,1401	
1584	ZNF17	ARCHS4 Coexpression,1531;Enrichr Queries,1270;GTEx Coexpression,1177	
1585	ZNF774	ARCHS4 Coexpression,1488;Enrichr Queries,1210;GTEx Coexpression,1281	
1586	ZNF107	ARCHS4 Coexpression,1513;Enrichr Queries,1263;GTEx Coexpression,1207	
1587	ZNF85	ARCHS4 Coexpression,1547;Enrichr Queries,1191;GTEx Coexpression,1254	
1588	ZNF358	ARCHS4 Coexpression,1483;Enrichr Queries,962;GTEx Coexpression,1554	VEGFA
1589	MTERF1	ARCHS4 Coexpression,1609;GTEx Coexpression,1058	
1590	ZNF569	ARCHS4 Coexpression,1403;Enrichr Queries,1323;GTEx Coexpression,1288	
1591	KCNIP3	ARCHS4 Coexpression,1351;GTEx Coexpression,1327	
1592	ZNF100	ARCHS4 Coexpression,1485;Enrichr Queries,1361;GTEx Coexpression,1171	
1593	ZNF334	ARCHS4 Coexpression,1505;Enrichr Queries,1295;GTEx Coexpression,1218	
1594	ZNF69	ARCHS4 Coexpression,1498;Enrichr Queries,1401;GTEx Coexpression,1121	
1595	FAM170A	ARCHS4 Coexpression,1344;Enrichr Queries,1293;GTEx Coexpression,1389	
1596	ZNF880	ARCHS4 Coexpression,1097;GTEx Coexpression,1590	
1597	ZNF670	ARCHS4 Coexpression,1324;Enrichr Queries,1370	
1598	ZNF428	ARCHS4 Coexpression,1383;Enrichr Queries,1318;GTEx Coexpression,1375	
1599	NKX26	ARCHS4 Coexpression,1501;GTEx Coexpression,1217	
1600	LIN54	ARCHS4 Coexpression,1461;GTEx Coexpression,1260	
1601	ZNF578	ARCHS4 Coexpression,1535;Enrichr Queries,1111;GTEx Coexpression,1436	EBF1
1602	PRRX2	ARCHS4 Coexpression,1566;Enrichr Queries,957;GTEx Coexpression,1592	EBF1
1603	OSR1	ARCHS4 Coexpression,1583;GTEx Coexpression,1166	
1604	HSF4	ARCHS4 Coexpression,1519;Enrichr Queries,1048;GTEx Coexpression,1563	VEGFA
1605	DUXA	ARCHS4 Coexpression,1260;Enrichr Queries,1284;GTEx Coexpression,1602	
1606	TOPORS	ARCHS4 Coexpression,1414;GTEx Coexpression,1358	
1607	SKI	ARCHS4 Coexpression,1468;GTEx Coexpression,1305	
1608	FLYWCH1	ARCHS4 Coexpression,1579;GTEx Coexpression,1206	
1609	THAP9	ARCHS4 Coexpression,1476;Enrichr Queries,1328;GTEx Coexpression,1400	
1610	ZNF322	ARCHS4 Coexpression,1466;GTEx Coexpression,1340	
1611	AEBP1	ARCHS4 Coexpression,1323;GTEx Coexpression,1484	
1612	MSANTD1	ARCHS4 Coexpression,1619;GTEx Coexpression,1195	

1613	ZNF479	ARCHS4 Coexpression,1392;Enrichr Queries,1321;GTEx Coexpression,1522
1614	GLI4	ARCHS4 Coexpression,1286;GTEx Coexpression,1579
1615	ZNF658	ARCHS4 Coexpression,1592;Enrichr Queries,1286
1616	DBX2	ARCHS4 Coexpression,1596;Enrichr Queries,1277;GTEx Coexpression,1444
1617	ZNF853	ARCHS4 Coexpression,1378;GTEx Coexpression,1516
1618	TIGD4	ARCHS4 Coexpression,1379;Enrichr Queries,1373;GTEx Coexpression,1599
1619	CENPB	ARCHS4 Coexpression,1474;Enrichr Queries,1356;GTEx Coexpression,1524
1620	ZNF33A	ARCHS4 Coexpression,1494;Enrichr Queries,1319;GTEx Coexpression,1580
1621	NKX25	ARCHS4 Coexpression,1580;GTEx Coexpression,1379
1622	NKX62	ARCHS4 Coexpression,1563;GTEx Coexpression,1403
1623	ZBED6	ARCHS4 Coexpression,1497
1624	TWIST2	ARCHS4 Coexpression,1412;GTEx Coexpression,1587
1625	ZNF814	ARCHS4 Coexpression,1599;GTEx Coexpression,1411
1626	MSANTD3	ARCHS4 Coexpression,1554;GTEx Coexpression,1467
1627	NACC2	ARCHS4 Coexpression,1514;GTEx Coexpression,1529
1628	MYPOP	ARCHS4 Coexpression,1532;GTEx Coexpression,1512
1629	CC2D1A	ARCHS4 Coexpression,1493;GTEx Coexpression,1565
1630	ZNF469	ARCHS4 Coexpression,1525;GTEx Coexpression,1534
1631	L3MBTL3	ARCHS4 Coexpression,1601;GTEx Coexpression,1473
1632	ZNF883	ARCHS4 Coexpression,1521;GTEx Coexpression,1581
